# Single-cell imaging analysis, therapeutic modeling and a Phase Ib trial validate BCL-2 as a target across heterogeneous castration-resistant prostate cancer

**DOI:** 10.1038/s41392-026-02700-w

**Published:** 2026-05-01

**Authors:** Anmbreen Jamroze, Xiaozhuo Liu, Surui Hou, Wen (Jess) Li, Han Yu, Amanda Tracz, Justine Jacobi, Qiuhui Li, Kent Nastiuk, Xin Chen, Jiaoti Huang, Kevin Lin, Mingyu Liu, Changmeng Cai, Yue Lu, Igor Puzanov, Jason S. Kirk, Gurkamal Chatta, Dean G. Tang

**Affiliations:** 1 Department of Pharmacology & Therapeutics, Roswell Park Comprehensive Cancer Center, Buffalo, NY USA; 2 Department of Biostatistics & Bioinformatics, Roswell Park Comprehensive Cancer Center, Buffalo, NY USA; 3 Experimental Therapeutics (ET) Graduate Program, Roswell Park Comprehensive Cancer Center and the University at Buffalo, Buffalo, NY USA; 4 Departments of Cancer Genetics and Urology, Roswell Park Comprehensive Cancer Center, Buffalo, NY USA; 5 Department of Pathology, Duke University School of Medicine, Durham, NC USA; 6 Department of Epigenetics and Molecular Carcinogenesis, the University of Texas M.D Anderson Cancer Center, Houston, TX USA; 7 Center for Personalized Cancer Therapy, University of Massachusetts Boston, Boston, MA USA; 8 Department of Medicine, Roswell Park Comprehensive Cancer Center, Buffalo, NY USA; 9 Present Address: Department of Medicine and Department of Biomedical Sciences, Cedars-Sinai Medical Center, and Samuel Oschin Comprehensive Cancer Institute, Cedars-Sinai Medical Center, Los Angeles, CA USA; 10 Present Address: State Key Laboratory Breeding Base of Basic Science of Stomatology (Hubei-MOST) and Key Laboratory for Oral Biomedicine of Ministry of Education (KLOBM), School and Hospital of Stomatology, Wuhan University, Wuhan, China; 11 Present Address: Department of Oncology, Tongji Hospital, Tongji Medical College, Huazhong University of Science and Technology, Wuhan, China

**Keywords:** Drug development, Urological cancer, Cancer stem cells, Tumour heterogeneity, Cancer models

## Abstract

BCL-2 has been implicated in prostate cancer (PCa) progression and development of castration-resistant disease (CRPC); however, it remains unclear how the BCL-2- and AR-expressing PCa cell populations evolve across the PCa continuum, how AR molecularly regulates BCL-2 and whether BCL-2 represents a common therapeutic target in heterogeneous CRPC. Here we first show the selective induction of BCL-2 by AR pathway inhibitors (ARPIs). Vectra-based quantitative multiplex immunofluorescence (qmIF) and image mass cytometry (IMC) analyses with single-cell resolution in patient PCa and xenograft models reveal markedly increased BCL-2^+^ (AR^+^ or AR^-^) PCa cells in CRPC. Mechanistically, AR represses *BCL-2* transcription through several AR binding sites and ARPIs relieve this repression. Therapeutic studies in cells, organoids and xenografts support BCL-2 as a shared vulnerability across diverse CRPC subtypes. A Phase Ib clinical trial (NCT03751436) combining enzalutamide and BCL-2 inhibitor venetoclax demonstrated reduced circulating tumor cells in responding patients. In summary, by integrating high-content single-cell level imaging analyses with mechanistic studies, extensive preclinical therapeutic experiments and a Phase Ib clinical trial, our studies herein elucidate the AR^+/-^BCL-2^+/-^ PCa cell subpopulation dynamics and credentials BCL-2 as a vital therapeutic target in heterogeneous CRPC.

## Introduction

Prostate cancer (PCa) continues to claim a high mortality with >35,000 American men estimated to die from metastatic castration-resistant PCa (mCRPC) in 2025.^1^ The incidence rates of advanced PCa have increased significantly and the proportion of men diagnosed with distant metastases has doubled from 2011 to 2019 when the second-line androgen receptor (AR) pathway inhibitors (ARPIs) such as abiraterone and enzalutamide (Enza) were introduced to the clinic. Recent studies have linked both the increased diagnosis of advanced and metastatic PCa and persistently high PCa mortality to therapy resistance driven by PCa cell heterogeneity and plasticity.^2–8^

Advanced PCa is treated with androgen deprivation therapy (ADT) with or without ARPIs. ADT/Enza are effective in targeting AR-expressing (AR^+^) PCa cells; however, PCa at all stages, from newly diagnosed primary tumor to mCRPC, harbors AR^-/lo^ PCa cells expressing little or no AR,^4,5,9–23^ which we have shown to be de novo refractory to Enza.^20^ Moreover, the AR^-/lo^(PSA^-/lo^) PCa cells pre-exist in treatment-naïve tumors and exhibit gene expression profiles and functional properties of PCa stem cells (PCSCs).^5,14–18,22,24–26^ CRPC is often characterized with increased AR^-/lo^ PCa cells, which may stem from an expansion of pre-existing AR^-/lo^ population and/or from ARPI-induced reprogramming (plasticity or de-differentiation) of AR^+^ to AR^-/lo^ cells.^5,8,14,15,17,20,21,23^ ARPIs induce PCa cell plasticity via several inter-woven mechanisms involving genetic mutations, epigenetic plasticity, and transcriptional and metabolic re-wiring.^8^ In principle, to achieve enduring clinical efficacy, both cancer cell heterogeneity and therapy-induced plasticity and both AR^+^ and AR^-/lo^ PCa cell populations should be therapeutically targeted.^5^

We have recently reported, in experimental and patient CRPC, three CRPC subtypes with distinct AR expression levels and patterns, i.e., AR^+/hi^ (high nuclear AR), AR^-/lo^ (minimal or absent AR) and AR^cyto^ (predominantly cytoplasmic AR with ~10–15% nuclear AR).^20^ These three CRPC subtypes manifest distinct responses to Enza: AR^+/hi^ LNCaP-CRPC exhibits an acute and transient sensitivity and AR^cyto^ LAPC4-CRPC and VCaP-CRPC a more drawn-out sensitivity whereas AR^-/lo^ LAPC9-CRPC shows no response to Enza.^20^ Importantly, this study nominated BCL-2 as a potential molecular driver and therapeutic target for CRPC.^20^

BCL-2 is a well-studied prosurvival and stemness factor and has been implicated in progression and therapy resistance of many blood and solid cancers including PCa.^27–50^ Early studies from our lab have shown the preferential expression of BCL-2 in AR^-/lo^ PCSCs,^14,17^ critical prosurvival functions of BCL-2 in PCa cells under nutrient deprivation and oxidative and genotoxic stresses,^29,32^ and induction of BCL-2 in PCa models by chemotherapeutic drugs, castration and castration/Enza.^20,29,32,37,43^ Consistently, castration, ARPIs, chemotherapeutic drugs, prooxidants and radiation have all been reported to induce BCL-2, which in turn promotes PCa cell survival and resistance to such agents.^20,27–29,32,35,37,40,42,43,45–50^ Therefore, BCL-2 has long been considered a therapeutic target in PCa, and several types of BCL-2-targeting therapeutics have been developed for the clinic, including antisense oligonucleotides (ASO; oblimersen sodium),^51–55^ siRNA,^56^ Gossypol (a polyphenol extract from cottonseed) and its derivative AT-101 (R-(-)-gossypol acetic acid),^57,58^ and BH3 mimetics such as HA14-1,^59^ WL-276,^60^ Venetoclax (ABT-199),^61–64^ and recently, Lisaftoclax (APG-2575)^65–67^ and Sonrotocalx (BGB-11417).^68,69^

Despite these developments, BCL-2 inhibitors have not yet reached the clinic for PCa, and significant knowledge gaps remain. For example, we lack a holistic understanding of the dynamic changes in, and interrelationship between, BCL-2- and AR-expressing PCa cells across the PCa evolutionary continuum; it is incompletely understood how AR regulates BCL-2 under different androgen conditions and how ARPIs trigger the upregulation of BCL-2; and it remains unclear whether BCL-2 represents a common therapeutic target across diverse CRPC subtypes such as AR^+/hi^, AR^cyto^ and AR^-/lo^ CRPC.^20^ Herein, we address these knowledge gaps by employing human PCa specimens and in vivo and in vitro models of CRPC subtypes, combined with high-content quantitative imaging analyses, mechanistic studies, and therapeutic evaluations using organoid and xenograft systems. These investigations validate BCL-2 as a critical therapeutic target in heterogeneous and plastic CRPC. In addition, we present correlative data from a Phase Ib clinical trial^70^ combining Enza with Venetoclax in mCRPC patients (NCT03751436), showing that this combination reduced circulating tumor cells (CTCs) in several ‘responsive’ patients, underscoring the translational relevance of this therapeutic strategy.

## Results

### BCL-2 is selectively and consistently induced by castration in patient PCa and xenograft models

**Fig. 1:**
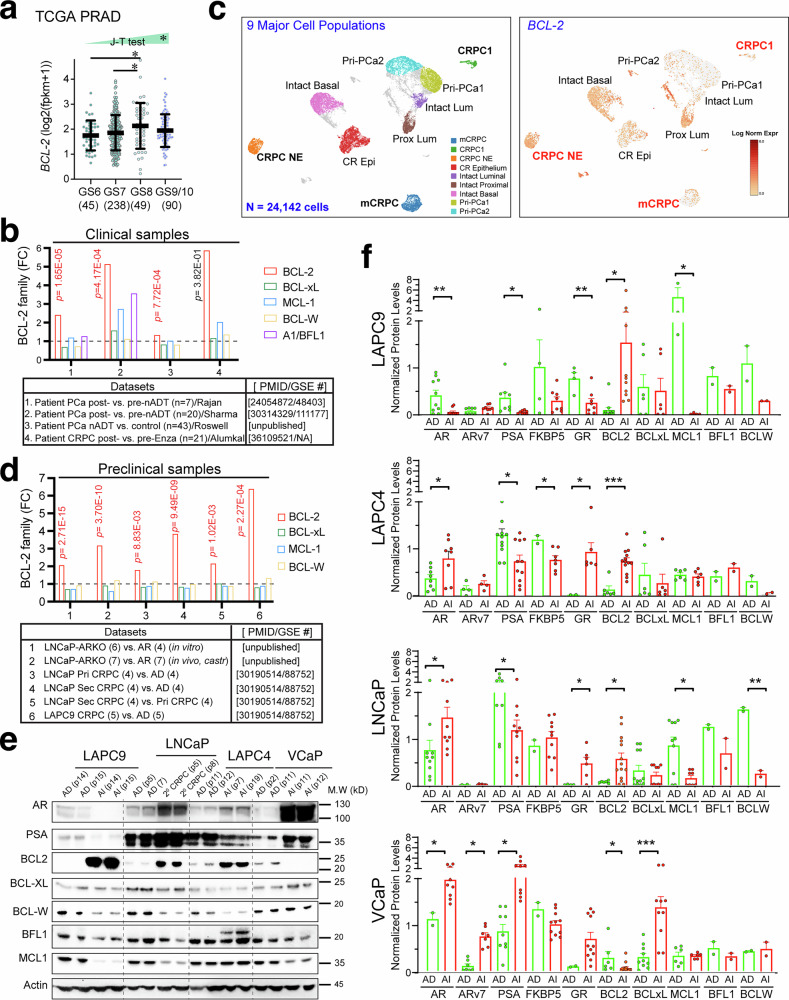
Selective upregulation of BCL-2 by castration (ADT) and antiandrogens. **a** *BCL-2* mRNA levels are increased in high-grade PCa and correlate with tumor grade. Data were extracted from TCGA_PRAD consisting of PCa with increasing grade (GS, Gleason Score; n indicated below). *p < 0.05 (Student’s *t*-test). J-T trend test showed that *BCL-2* mRNA levels correlated with increasing tumor grade (*p < 0.05). **b** *BCL-2* mRNA (among 5 members) was upregulated in patients’ PCa treated with nADT (the first 3 datasets) and showed a trend of upregulation in the Alumkal pre-/post-Enza cohort (i.e., dataset 4). Note that *A1/BFL-1* was not detected in datasets 3 and 4. **c** *BCL-2* mRNA is elevated in CRPC compared to Pri-PCa in a scRNA-seq dataset.^75^ Shown on the left are the 9 major cell populations we recently re-mapped out of the total of 24,142 cells.^76^ Shown on the right is the *BCL-2* mRNA expression in the 9 cell populations. Note the higher *BCL-2* levels in the 3 CRPC (CRPC1, mCRPC and CRPC NE) samples than in the 2 Pri-PCa (Pri-PCa1 and Pri-PCa2). **d**, **e** BCL-2 family mRNA (**d**) and protein (**e**) levels in AD/AI (CRPC) xenograft models. **d** *BCL-2* mRNA is commonly increased in 6 CRPC datasets (n indicated in parentheses). Datasets 1 and 2 were comparisons of LNCaP clones with intact AR vs. AR knockout (ARKO) either cultured in vitro (dataset 1) or propagated in castrated NSG mice (dataset 2). Datasets 3–6 were from our earlier publication (PMID and GSE# indicated). **e** AD and AI tumors serially passaged (p; indicated in parentheses) were used in WB analysis of the proteins indicated. 20 μg protein was loaded for each lane and actin was used as loading control. **f** Quantification of the normalized protein levels of AR, ARv7, PSA, FKBP5, GR, and the 5 BCL-2 members (BCL-2, BCL-xL, MCL-1, BCL-W, and BFL-1/A1) in our 4 paired AD/AI xenograft models. Each circle (data point) represents the target protein band from an independent WB experiment. Protein abundance was normalized to housekeeping proteins (GAPDH and/or actin) to enable direct comparison of absolute expression levels across models and conditions. Group differences were evaluated using unpaired two-tailed Student’s *t*-tests, with statistical parameters reported according to GraphPad Prism (*p < 0.05; **p < 0.01; ***p < 0.001)

We first examined a transcriptomic dataset^71^ (supplementary Table 1) for *BCL-2* mRNA levels in 3 epithelial cell (CD45^-^EpCAM^+^) populations of the normal human prostate, i.e., basal (B; CD49f^hi^CD38^lo^), luminal (L; CD49f^lo^CD26^+^CD38^hi^) and luminal progenitor (LP; CD49f^lo^CD26^+^CD38^lo^) cells. We found that *BCL-2* mRNA was expressed at high levels in LP and basal cells but had lower expression in mature luminal cells (supplementary Fig. 1a). We then examined *BCL-2* levels in 422 untreated primary tumors in TCGA-PRAD, which displayed an increasing trend that correlated with tumor grade (i.e., combined Gleason Score, GS) as supported by Jonckhere-Terpstra (J-T) trend test (Fig. 1a). We subsequently investigated the mRNA levels of all 5 prosurvival BCL-2 family members, i.e., BCL-2, BCL-xL, MCL-1, BCL-W and A1/BFL-1, in 4 treated patient datasets including 3 datasets of PCa patients subjected to neoadjuvant ADT (nADT; short-term (~ 2–6 months) ADT prior to prostatectomy)^72–74^ and 1 dataset of mCRPC patients who failed long-term ADT/Enza.^23^ As shown in Fig. 1b, *BCL-2* was the only member that was commonly and consistently induced in the 3 nADT datasets and showed a trend of upregulation in Enza-resistant PCa. Finally, we interrogated a recent scRNA-seq dataset^75^ consisting of FACS-purified human benign prostate epithelial cell subpopulations, 2 primary PCa (Pri-PCa), and 3 CRPC including CRPC1 and CRPC NE in the prostate and one mCRPC (Fig. 1c). We re-mapped this dataset^76^ and used *AMACR* to separate PCa cells from benign epithelial cells, which clustered the 24,142 high-quality cells to 9 major cell populations including 4 benign and 5 PCa cell populations (Fig. 1c, left; supplementary Fig. 1b, c). Interestingly, the 2 Pri-PCa was characterized by high levels of *KLK3* and *ERG* but lack of *KRT5* and *KRT14* expression whereas the 3 CRPC samples all lacked *KRT5*, *KRT14* and *ERG* expression and 2 of the 3 CRPC (i.e., CRPC1 and mCRPC) showed high *KLK3* expression (supplementary Fig. 1d–g). Importantly, *BCL-2* mRNA was enriched in proximal luminal (stem), basal and castration-resistant epithelial cells and was expressed at higher levels in 3 CRPC compared to 2 Pri-PCa (Fig. 1c). In contrast, *BFL-1* was barely detected and *BCL-xL, BCL-W* and *MCL1* showed similar levels in CRPC and Pri-PCa (supplementary Fig. 1h).

Our laboratory has developed 4 castration-resistant or androgen-independent (AI; also called primary (1°) CRPC) xenograft models, i.e., LNCaP, LAPC9, VCaP, and LAPC4, by serially passaging the parent androgen-dependent (AD) tumors in castrated mice^20^ (supplementary Fig. 2a). As in our previous findings,^20^ IHC characterization of the AR using the AR 441 monoclonal antibody (supplementary Table 2), which was raised against the aa 299-315 in the N-ter domain of AR and recognizes the full-length AR as well as all C-ter truncated AR variants (ARvs),^20^ revealed the LNCaP-AI tumors to be (nuclear) AR^+/hi^ and LAPC9-AI tumors AR^-/lo^ whereas LAPC4-AI and VCaP-AI tumors largely AR^cyto^ (with ~10% AR in the nucleus;^20^ supplementary Fig. 2b). These AI models, when compared to the corresponding AD tumors, showed model-specific changes in AR, ARv7, AR signaling activity, and glucocorticoid receptor (GR) (supplementary Figs. 2c–h, 3, see below).^20^ Consistent with our early studies,^20^ the VCaP-AI was the only model that expressed ARv7 (supplementary Figs. 2d, f, g; 3b). We systematically analyzed the mRNA and protein levels of BCL-2 family members in these AD/AI tumors and related models (Fig. 1d–f; supplementary Figs. 2c–h, 3). We observed that *BCL-2* mRNA was commonly and exclusively upregulated in castration-resistant (primary CRPC), castration/Enza-resistant (2° CRPC) and AR-knockout (ARKO) LNCaP models as well as in AR^-/lo^ LAPC9 CRPC^20^ (Fig. 1d). At the protein level, BCL-2 protein was upregulated in 3 of the 4 AI models except VCaP while the other 4 prosurvival BCL-2 family proteins showed model-dependent changes (Fig. 1e ; supplementary Figs. 2c–h, 3), as supported by quantitative analysis of western blotting (WB) results from 10 independent experiments with multiple AD/AI tumors for each model (*n* = 2–15 tumors) (Fig. 1f). Briefly, BCL-xL was upregulated in VCaP-AI (the only model that expressed ARv7), MCL-1 was downregulated in LAPC9-AI and LNCaP-AI, and BCL-W was reduced in LNCaP-AI whereas BFL1 did not change in any of the 4 AI models (Fig. 1e, f; supplementary Fig. 3).

In summary, these data from both PCa models and patient specimens indicate that *castration (ADT and/or ADT/Enza) commonly and selectively upregulates BCL-2*.

### Treatment-naive primary prostate tumors are populated mostly by AR^+^BCL-2^-^ PCa cells

**Fig. 2:**
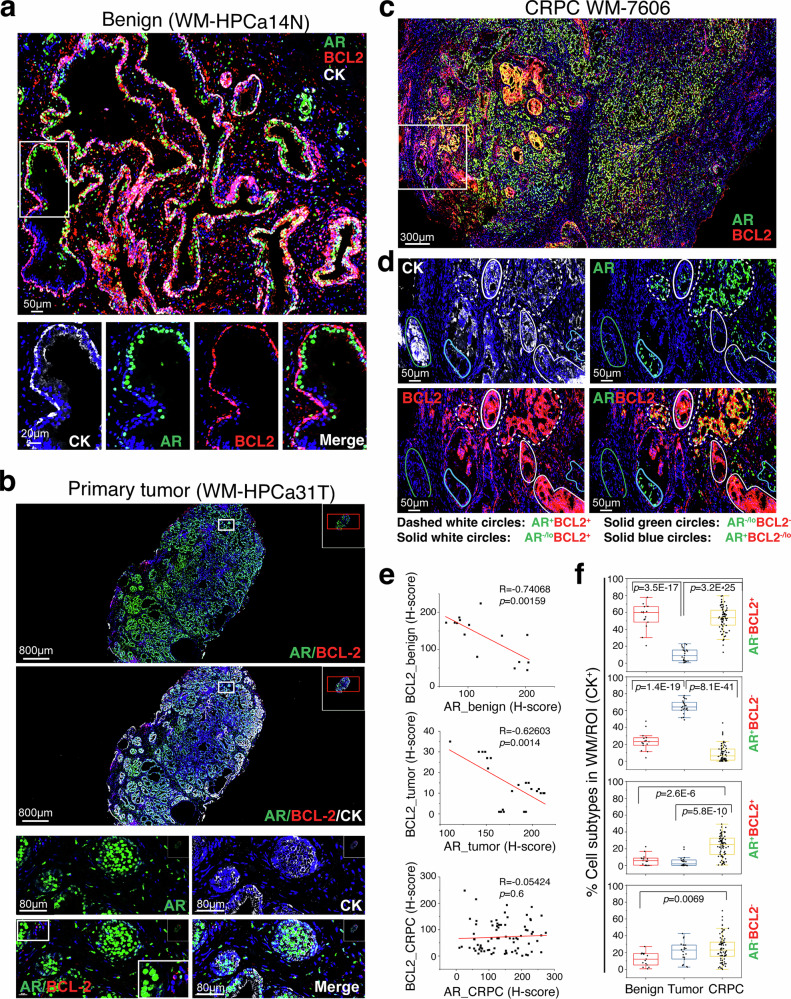
qmIF analysis reveals predominant AR^+^BCL-2^-^ cells in untreated primary PCa and markedly increased (AR^+^ or AR^-^) BCL-2^+^ cells in CRPC. **a** In benign prostatic glands (HPCa14N), BCL-2^+^ cells are mainly in the basal cell layer whereas AR^+^ cells in luminal layer. Cytokeratin (CK) staining was used to mark epithelial compartment. Note both AR and BCL-2 were also expressed in stromal cells (scale bar, 50 μm). Magnified images of individual stains from the boxed region in the whole-mount (WM) image (top) were presented below (scale bar, 20 μm for all 4 lower panels). **b** Primary PCa is characterized by significantly increased AR^+^BCL-2^-^ cells. Shown above are two WM images of HPCa31T (scale bars, 800 μm) and down below zoom-in images of individual or merged markers (scale bars, 80 μm). **c**, **d** Increased cellular heterogeneity and markedly expanded (AR^+^ or AR^-^) BCL-2^+^ cell population in CRPC. **c** WM low-magnification image showing AR/BCL-2 expression (scale bar, 300 μm). **d** Zoom-in images of the boxed area in c showing individual markers (scale bar, 50 μm). **e** Relationship between (CK^+^) AR-expressing and/or BCL-2-expressing cells in benign tissue (top), primary tumor (middle) and CRPC (bottom). Regression line, and Pearson R and *P* values are indicated. **f** Box plots summarizing the relative % of 4 PCa cell subtypes in WM images analyzed in benign tissues, primary tumors and CRPC. Each dot in the box plots represents a CK^+^ ROI. *P* values were determined by repeated measures two-way ANOVA with Bonferroni multiple comparison test

The above observations suggest a reciprocal relationship between AR signaling and BCL-2 expression. To confirm and extend these findings, we employed Vectra-based quantitative multiplex immunofluorescence (qmIF) to assess PCa cells expressing BCL-2 and/or AR in regular FFPE (formalin-fixed and paraffin-embedded) sections as well as TMA (tissue microarray) and whole-mount (WM) sections from benign prostate (*n* = 123), treatment-naïve primary PCa (*n* = 125) and treatment-failed CRPC (*n* = 25) (Fig. 2; supplementary Figs. 4–8, supplementary Table 3). We utilized cytokeratin (CK) staining to demarcate the epithelial compartment. Consistent with earlier reports^27,38,41,44^ and mRNA data (Fig. 1c; supplementary Fig. 1a), BCL-2 protein was expressed in the basal cell layer while AR was detected in luminal layer in benign glands (Fig. 2a; supplementary Figs. 4c, 5a), and BCL-2^+^ cells and AR^+^ cells displayed a strong inverse correlation in benign prostate (R = −0.74, *p* = 0.00159; Fig. 2e, top). In untreated Pri-PCa, there was a striking expansion of AR^+^ and AR^+^BCL-2^-^ cell populations compared to benign tissues (Fig. 2b, f; supplementary Figs. 4d, 5b, 8c) and the AR^+^ and BCL-2^+^ PCa cells still exhibited an inverse correlation (R = −0.626, *p* = 0.0014; Fig. 2e, middle). Consistent with decreases in BCL-2^+^ PCa cells, the *BCL-2* mRNA levels were reduced in Pri-PCa compared to benign tissues (supplementary Fig. 8a, b).

Cell subtype analysis revealed that among all CK^+^ ROIs (Region of Interest, 1 mm^2^) in benign prostate, the AR^-^BCL-2^+^ population represented the majority followed by AR^+^BCL-2^-^ cells, with low representation of AR^+^BCL-2^+^ (double positive) and AR^-^BCL-2^-^ (double-negative) cells (Fig. 2f; supplementary Fig. 8d–f). In Pri-PCa, the AR^+^BCL-2^-^ cells became the predominant cell population followed by AR^-^BCL-2^-^ cells with low representation of the double-positive and AR^-^BCL-2^+^ cell subtypes (Fig. 2f; supplementary Fig. 8d–f).

### Castration drives PCa cell heterogeneity and upregulates BCL-2^+^ PCa cells in patient CRPC

We analyzed a total of 25 CRPC including 20 in TMA and 5 WM sections (supplementary Fig. 4b) and found that the reciprocal relationship between BCL-2^+^ and AR^+^ PCa cells observed in Pri-PCa was lost in CRPC (Fig. 2e). Relative to Pri-PCa, CRPC showed reduced fraction of AR^+^ but significantly increased fraction of BCL-2^+^ PCa cells (supplementary Fig. 8c). Notably, we observed increased PCa cell heterogeneity: AR^-^BCL-2^+^ cells, typical of the LAPC9-AI cells (Fig. 1e, f; supplementary Fig. 2b–d), constituted ~50% of the 4 cell-type (AR^-^BCL-2^+^, AR^+^BCL-2^-^, AR^-^BCL-2^-^ and AR^+^BCL-2^+^) subpopulations amongst (CK^+^) CRPC cells (Fig. 2d, f; supplementary Figs. 5c–d, 6c, 7, 8d–f). We also observed significant increases in AR^+^BCL-2^+^ PCa cells in CRPC (Fig. 2d, f; supplementary Figs. 6b, 8d–f), which were characteristic of LNCaP 1^o^/2^o^ CRPC cells (Fig. 1e, f). Aggregating cell density (cell count/mm^2^) in WM sections (supplementary Fig. 8e) or in all specimens (supplementary Fig. 8f) and compared to Pri-PCa, we observed that the AR^+^BCL-2^-^ PCa cells represented the majority in primary tumors whereas in CRPC the AR^-^BCL-2^+^ cells became the predominant cell population accompanied by increased AR^+^BCL-2^+^ and AR^-^BCL-2^-^ cells (Fig. 2f; supplementary Fig. 8d). Of interest, in patient CRPC, while the majority of AR^+^ PCa cells showed nuclear AR, we also observed increased LAPC4-AI-like AR^cyto^BCL-2^+^ (supplementary Fig. 7b, d) and VCaP-AI-like AR^cyto^BCL-2^-^ (supplementary Fig. 7d, e) cells.

### Castration-induced dynamic changes in AR^+/-^BCL-2^+/-^ PCa cell subpopulations in xenograft tumors recapitulate the changes observed in patient CRPC

**Fig. 3:**
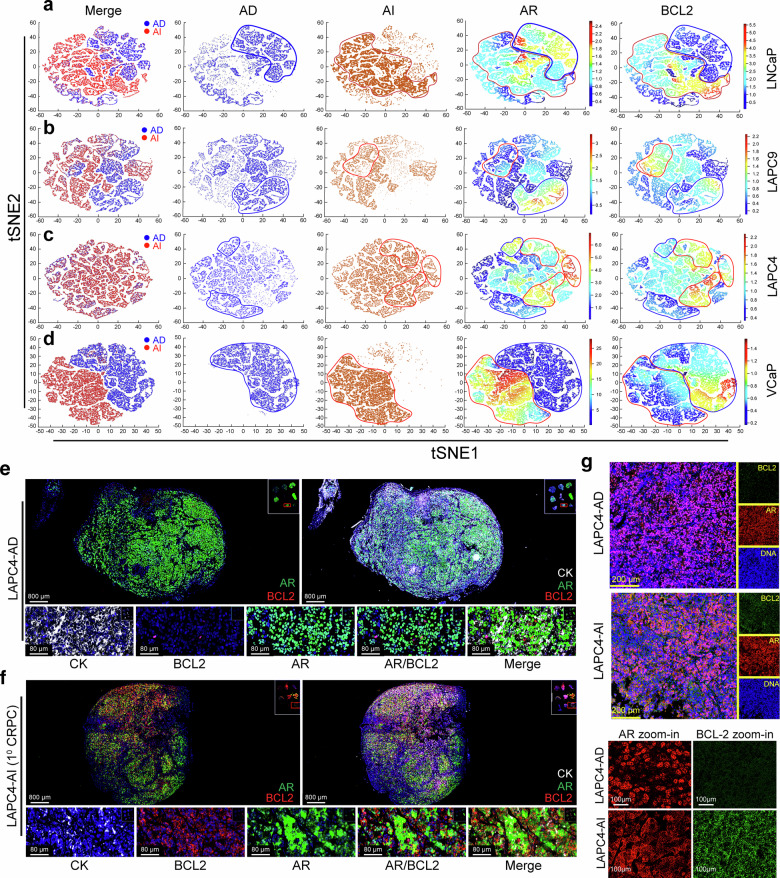
Vectra and IMC analysis reveals castration-induced dynamic changes in AR^+/-^BCL-2^+/-^ cell types in 4 xenograft CRPC models. **a**–**d** tSNE plots of IMC-derived single-cell imaging data. Shown are the individual and merged tSNE plots from LNCaP-AD/AI (**a**), LAPC9-AD/AI (**b**), LAPC4-AD/AI (**c**), and VCaP-AD/AI (**d**) xenografts. Columns represent merged AD and AI cell clusters (first column), individual AD and AI cell populations and contours (second and third columns), and AR and BCL-2 expression overlaid on tSNE maps (fourth and fifth columns). Blue and red contours indicate AD and AI cell subpopulations, respectively. **e** LAPC4-AD tumors are populated mostly by AR^+^BCL-2^-^ PCa cells. Shown on top are qmIF WM images (scale bar, 800 μm) and at the bottom zoom-in images (scale bar, 80 μm for all panels). **f** LAPC4-AI (1° CRPC) tumors are populated by AR^cyto^BCL-2^+^ PCa cells. Shown on top are qmIF WM images (scale bar, 800 μm) and at the bottom zoom-in images illustrating cytoplasmic AR^+^ LAPC4-AI cells with upregulated BCL-2 (scale bar, 80 μm for all panels). **g** IMC images of AR and BCL-2 in LAPC4-AD and LAPC4-AI tumors (scale bar, 200 μm) with representative zoom-in images of AR and BCL-2 shown below (scale bar, 100 μm for all panels)

Next, we employed both Vectra-based qmIF and Imaging Mass Cytometry (IMC) platforms to quantitatively assess the dynamics of AR^+/-^BCL-2^+/-^ cells in the 4 PCa AD/AI xenograft models developed in our lab (Fig. 3; supplementary Figs. 9–12), which showed model-related alterations in AR, ARv7, AR activity (PSA and FKBP5), and GR (Fig. 1e–f; supplementary Figs. 2 and 3). Re-analysis of individual AI tumors under therapy^20^ also revealed distinct Enza responses in 1^o^ CRPC associated with the AR heterogeneity: AR^+/hi^ LNCaP-AI responded to Enza for ~4 weeks (supplementary Fig. 9a) and AR^-/lo^ LAPC9-AI were de novo refractory to Enza (supplementary Fig. 9b) whereas AR^cyto^ LAPC4-AI and VCaP-AI responded to Enza with protracted latencies prior to emergence of castration/Enza-resistant 2° CRPC (supplementary Fig. 9c, d).

In the LNCaP-AD/AI system (Fig. 3a; supplementary Fig. 10), qmIF analysis of WM LNCaP-AD tumors revealed that both AR^-^ and AR^+^ cells expressed little BCL-2 (supplementary Fig. 10b). Most cells in LNCaP-AI (1° CRPC) tumors turned AR^+/hi^ with slightly increased BCL-2 (supplementary Fig. 10c). Sensitive IMC-generated single-cell profiling with tSNE-based cell clustering corroborated AR and BCL-2 upregulation in LNCaP-AI cells (Fig. 3a; supplementary Fig. 10e). Strikingly, most PCa cells in castration/Enza-resistant 2° LNCaP-CRPC were AR^+/hi^BCL-2^+/hi^ (supplementary Fig. 10d).

The LAPC9-AD tumors resembled LNCaP-AD tumors in containing both AR^-^BCL-2^-^ and AR^+^BCL-2^-^ cells (supplementary Fig. 11a). In contrast, LAPC9-AI tumors, unlike LNCaP-AI tumors, apparently evolved into AR^-^BCL-2^+/hi^ (supplementary Fig. 11b). IMC and tSNE-anchored cell subpopulation analyses also revealed reduced AR expression concomitant with elevated BCL-2, resulting in substantially increased AR^-^BCL-2^+^ cells in LAPC9-AI tumors (Fig. 3b; supplementary Fig. 11c).

Most LAPC4-AD tumor cells exhibited an AR^+^BCL-2^-^ phenotype on qmIF analysis (Fig. 3e) and castration reprogrammed LAPC4-AI cells to the AR^cyto^BCL-2^+^ phenotype (Fig. 3f). Single-cell analysis by IMC confirmed the qmIF-described AR^cyto^BCL-2^+^ phenotype in LAPC4-AI tumors (Fig. 3c, g). Finally, the VCaP AD/AI model was an exception, where most VCaP-AD tumor cells were AR^+^BCL-2^+^ while VCaP-AI tumor cells became AR^cyto^BCL-2^-/lo^ (supplementary Fig. 12a, b). IMC analysis validated the AR^+^BCL-2^+^ phenotype of VCaP-AD cells and demonstrated cytoplasmic AR redistribution and reduced BCL-2 in VCaP-AI tumors (Fig. 3d; supplementary Fig. 12c).

In summary, 3 of the 4 AD xenograft models (LNCaP, LAPC9 and LAPC4) recapitulated the major AR^+^BCL-2^-^ cellular phenotype observed in human Pri-PCa whereas VCaP-AD tumors, intriguingly, displayed an AR^+^BCL-2^+^ phenotype. Consistent with the diverse AR^+/-^BCL-2^+/-^ cell subtypes observed in patient CRPC, the 4 xenograft CRPC models manifested variegated cellular subpopulations: the LNCaP (1° and 2°) CRPC predominantly AR^+^BCL-2^+^, LAPC9-CRPC AR^-^BCL-2^+^, LAPC4-CRPC AR^cyto^BCL-2^+^ and VCaP-CRPC predominantly AR^cyto^BCL-2^-^ phenotype, respectively.

### Developing castration/Enza-resistant LAPC4 culture models to recapitulate castration-induced AR^cyto^BCL-2^+^ phenotype

**Fig. 4:**
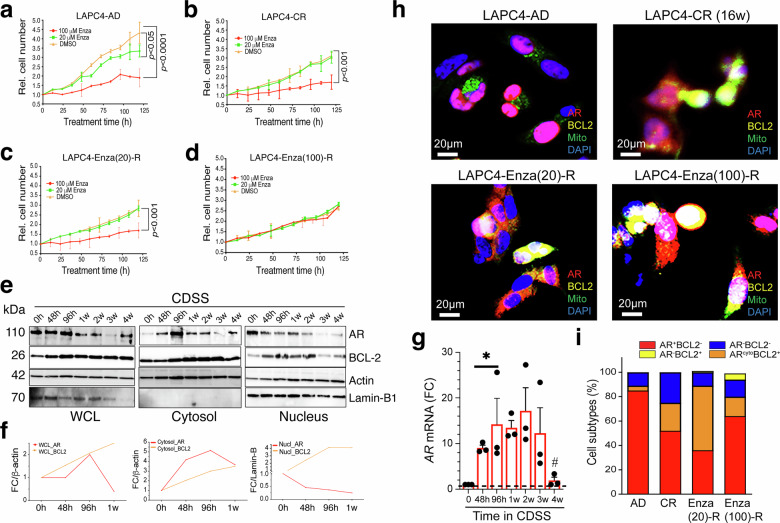
Enzalutamide resistance is associated with AR cytoplasmic relocalization and BCL-2 upregulation in the LAPC4 model. **a**–**d** Cell proliferation assays showing relative cell numbers over time in LAPC4-AD (**a**), LAPC4-CR (**b**), and two Enza-resistant sublines—LAPC4-Enza(20)-R (**c**) and LAPC4-Enza(100)-R (**d**). Shown are the mean ± SEM (*p* < 0.001, two-way ANOVA). **e**. Immunoblot analysis of LAPC4-AD cells cultured in CDSS over time shows dynamic changes in AR and BCL-2 expression using whole-cell lysates (WCL), and cytosolic and nuclear fractions. Lamin-B1 and actin serve as loading controls. **f** Densitometric quantification of immunoblots in (**e**) showing fold change (FC) of AR and BCL-2 relative to loading controls over the time course in WCL (left) and cytosolic (middle) and nuclear fractions (right). **g** Biphasic changes of *AR* mRNA levels in LAPC4 cells cultured in CDSS. *p < 0.05 when comparing 48 h and 96 h vs. 0 h; #*p* < 0.05 when comparing 4w vs. 3w. **h** IF staining of LAPC4-AD, LAPC4-CR, and Enza-resistant sublines (20 μM and 100 μM) showing AR (red), BCL-2 (yellow), mitochondria (green), and nuclei (DAPI, blue). Images reveal increased cytoplasmic AR and mitochondrial BCL-2 in resistant sublines. Scale bars, 20 μm. **i** Quantification of AR and BCL-2 expression patterns from the immunofluorescence data in (**h**), categorized into four groups: AR⁺BCL-2⁻, AR^cyto^BCL-2⁺, AR⁻BCL-2⁺, and AR⁻BCL-2⁻

Our previous analysis of 195 CRPC specimens revealed that as much as 40% of the CRPC had the mixed cytoplasmic/nuclear AR (i.e., largely AR^cyto^ phenotype with ~10–15% nuclear AR).^20^ qmIF studies herein also identified prominent AR^cyto^ CRPC cells, which often expressed high levels of BCL-2 and presented an AR^cyto^BCL-2^+^ phenotype (e.g., supplementary Fig. 7b, d), much like the AR^cyto^BCL-2^+^ LAPC4-CRPC in vivo (Fig. 3f, g). To further study the AR^cyto^BCL-2^+^ CRPC, we established the castration-resistant and dual castration- and Enza-resistant LAPC4 cell models (Fig. 4; supplementary Fig. 13). To this end, we cultured regular LAPC4-AD cells long-term (4 months) in charcoal dextran stripped serum (CDSS) medium to develop castration-resistant LAPC4 (LAPC4-CR or LAPC4-AI) cells, which were subsequently exposed to either 20 μM or 100 μM Enza-containing CDSS medium for 1 month, resulting in the LAPC4-Enza(20)-R or LAPC4-Enza(100)-R models (supplementary Fig. 13a). These CDSS and CDSS/Enza selected LAPC4 sublines displayed more elongated and mesenchymal morphology (supplementary Fig. 13b) and proliferated more slowly (supplementary Fig. 13c) than LAPC4-AD cells. Moreover, although LAPC4-AD cells were inhibited by both 20 μM and 100 μM of Enza (Fig. 4a), the LAPC4-CR and LAPC4-Enza(20)-R cells were sensitive only to 100 μM of Enza (Fig. 4b, c). LAPC4-Enza(100)-R cells were resistant to both 20 μM and 100 μM of Enza (Fig. 4d). WB analysis revealed a progressive redistribution of AR from nucleus to cytoplasm and increase in BCL-2 expression (Fig. 4e, f). Of interest, we observed a bi-phasic change in total AR protein levels in CDSS-cultured LAPC4 cells, which plateaued at 96 h then decreased afterwards (Fig. 4e, f, the WCL panels, left). RT-qPCR analysis revealed a time-dependent increase of *AR* mRNA levels within the 48–96 h of culturing LAPC4 cells in CDSS (Fig. 4g), suggesting that the early AR protein increase in CDSS-cultured LAPC4 cells (i.e., up to 96 h) was driven by castration-induced *AR* mRNA expression. However, castrated LAPC4 cells maintained steady high levels of *AR* mRNA levels from 96 h up to 3 weeks and only showed significant reduction of *AR* expression at 4 weeks (Fig. 4g), suggesting that the decreases in AR protein levels at ≥1 week in LAPC4-CDSS cells (Fig. 4e, f, left WCL panels) were due to post-transcriptional mechanisms.

Regardless, despite increased AR at both mRNA and protein levels within 48–96 h, the CDSS cultured LAPC4 cells showed immediate (i.e., within 48–96 h) AR protein redistribution from the nucleus to the cytosol (Fig. 4e, f). Multiplex IF imaging analysis also showed a shift from nuclear AR^+^ and low BCL-2 in LAPC4-AD to an AR^cyto^BCL-2^+/hi^ phenotype in resistant sublines, especially in LAPC4-Enza(100)-R cells in which BCL-2 co-localized with the Mito-tracker (Fig. 4h; supplementary Fig. 13d). The LAPC4-Enza(20)-R cells displayed an intermediate phenotype (supplementary Fig. 13d), suggesting an ongoing transition. Quantification of cell subtypes demonstrated that in LAPC4-AD cells, the AR^+^BCL-2^-^ phenotype predominated while the resistant sublines showed a prominent expansion in the AR^cyto^BCL-2^+^ subpopulation (Fig. 4i).

### *BCL-2* mRNA levels consistently anti-correlated with the AR activity

**Fig. 5:**
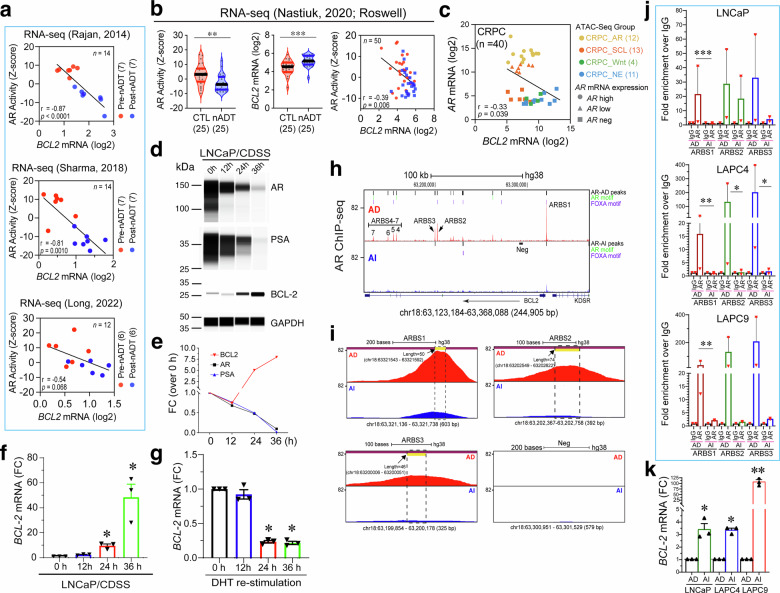
AR directly represses *BCL-2* transcription in PCa. **a** Inverse correlation between *BCL-2* mRNA levels and AR activity in 3 nADT cohorts. **b** Upregulation of *BCL-2* mRNA and inverse correlation between *BCL-2* mRNA levels and AR activity in the matched Roswell Park nADT cohort. **c** Inverse correlation between *BCL-2* and *AR* mRNA levels in the 40 CRPC specimens in the Tang F et al. dataset.^78^ **d**–**f** LNCaP cells castrated in vitro time-dependently downregulate AR and AR signaling but upregulate BCL-2. Shown are WB of AR, PSA and BCL-2 protein levels in LNCaP cells cultured in CDSS-containing medium for the time intervals indicated (**d**), densitometric quantification normalized to GAPDH (**e**), and changes in *BCL-2* mRNA levels measured by RT-qPCR (**f**; *n* = 3, mean ± SD; *p < 0.05). **g** LNCaP cells cultured in CDSS were exposed to DHT (1 nM) for the time intervals indicated, and *BCL-2* and *TBP* (TATA-binding protein; housekeeping gene) transcripts were quantified by qPCR. *BCL-2* expression was normalized to TBP and reported as fold change (FC) relative to 0 h (set to 1). Data are presented as mean ± SEM (*n* = 3). *p < 0.05 (Mann–Whitney test). **h** AR binds to multiple ARBS in *BCL-2* genomic locus. Shown are the 7 potential ARBS in LNCaP-AD (top) that are lost in LNCaP-AI (bottom) cells (Neg: genomic region used as negative control in ChIP-qPCR). Shown on right are AR peaks and canonical AR and FOXA motifs. **i** Zoom-in representation of ARBS1-3 in LNCaP-AD (top) vs. LNCaP-AI (bottom) cells. The sizes of the respective qPCR amplicons for ARBS1-3 are indicated by a yellow horizontal line. **j** ChIP–qPCR analysis of AR binding at the ARBS1–3 in the indicated 3 AD/AI xenograft models. Shown are the mean ± S.D of 3 independent experiments (*p < 0.05; **p < 0.01; ***p < 0.001; Student’s *t*-test). **k** *BCL-2* mRNA levels were elevated in the 3 AI models accompanying the loss of AR binding to ARBS. Total RNA extracted from the same AD/AI tumors as in j were employed in RT-qPCR analysis of *BCL-2* mRNA levels. Shown are the results from 3 independent AD/AI tumors for each model. *p < 0.05 and **p < 0.01 (Mann–Whitney test)

The preceding qmIF and IMC studies of BCL-2 and AR protein expression at the single-cell levels in patient CRPC and xenograft models revealed a reciprocal relationship between the two, suggesting that AR may transcriptionally represses *BCL-2*. In support, the mRNA levels of *BCL-2*, but not other BCL-2 family members, were *consistently* correlated, *inversely*, with the canonical AR signaling activity or AR-A^74^ in multiple clinical datasets of PCa patients who received nADT or failed long-term Enza treatment as well as in our AD/AI models (Fig. 5a–c; supplementary Figs. 14 and 15). Briefly, in 3 cohorts of PCa patients who underwent nADT,^72,73,77^ *BCL-2* mRNA levels exhibited a perfect inverse correlation with the AR-A: AR-A was high and *BCL-2* was low in pre-nADT PCa whereas AR-A was reduced but *BCL-2* levels were upregulated in patient-matched post-nADT samples (Fig. 5a; supplementary Fig. 14a–c). We also observed an inverse correlation between *BCL-2* mRNA levels and AR-A in an in-house RNA-seq dataset of 25 PCa patients treated for greater than 2 months with nADT compared with 25 stage/grade matched control cases (i.e., no nADT) (Fig. 5b; supplementary Fig. 14d). Among the other 4 BCL-2 family members, *BCL-2L1* (encoding BCL-xL) and *BCL-2L2* (encoding BCL-W) mRNA levels were each positively correlated with AR-A in 2 of the 4 nADT cohorts (supplementary Fig. 14a–d). In contrast, *MCL1* mRNA levels anti-correlated with AR-A in 3 (i.e., Rajan, Sharma, and Long) of the 4 nADT datasets and *BCL2A1* (encoding A1/BFL1) showed an inverse correlation with AR-A only in the Long cohort (supplementary Fig. 14a–d). Analysis of the Westbrook dataset consisting of 25 matched pre-/post-Enza mCRPC^23^ similarly revealed an anti-correlation between *BCL-2* mRNA and AR-A but a positive correlation between *BCL-2L2* and AR-A (supplementary Fig. 14e).

We further analyzed a dataset^78^ of 40 patient and organoid CRPC samples classified by the ATAC-seq (Assay for Transposase-Accessible Chromatin using sequencing) and RNA-seq analyses as CRPC_AR (*AR* high), CRPC_SCL (stem-cell like; *AR* low), CRPC_Wnt (*AR* negative), and CRPC_NE (*AR* negative) subtypes (Fig. 5c; supplementary Fig. 15a). We observed a moderate anti-correlation between *BCL-2* mRNA levels and AR-A and between *AR* vs. *BCL-2* mRNA levels, with the CRPC_AR subtype having the highest *AR* and lowest *BCL-2* while CRPC_Wnt and CRPC_NE the lowest *AR* but highest *BCL-2* (Fig. 5c; supplementary Fig. 15a). Interestingly, among the 13 CRPC_SCL samples, 7 co-segregated with the CRPC_AR subtype and 6 with the CRPC_Wnt/CRPC_NE (Fig. 5c), suggesting that the chromatin accessibility-stratified CRPC_SCL subtype is heterogeneous with ‘bifurcated’ *AR*^hi^*BCL*-2^lo^ and *AR*^lo^*BCL-2*^hi^ profiles. In this dataset, *BCL-2L1* (*BCL-xL*) mRNA levels positively correlated with AR-A (but not *AR* mRNA levels) whereas *BCL-2L2* (*BCL-W*) levels positively correlated with both *AR* mRNA levels and AR-A (supplementary Fig. 15a).

Finally, we observed a similar striking anti-correlation between *BCL-2* mRNA levels and AR-A in both AR^+/hi^ LNCaP-AI and AR^-/lo^ LAPC9-AI models, in which *BCL-2* mRNA was upregulated when AR-A became suppressed and *BCL-2* levels strongly anti-correlated with the AR-A (supplementary Fig. 15b–e).

### AR directly binds to the *BCL-2* genomic locus to repress its transcription

We subsequently conducted molecular studies to demonstrate that AR directly represses *BCL-2* transcription and ARPI relieves this inhibition leading to BCL-2 upregulation (Fig. 5d–k; supplementary Fig. 16). Experimental castration of LNCaP cells in CDSS time-dependently downregulated AR protein and inhibited AR activity (i.e., reducing PSA) over time (Fig. 5d, e) but induced BCL-2 mRNA and protein (Fig. 5e, f). In contrast, re-stimulation of CDSS-cultured LNCaP cells with DHT reduced *BCL-2* mRNA levels (Fig. 5g). We analyzed the AR chromatin immunoprecipitation sequencing (ChIP-seq) data^79^ from LNCaP cells under regular (AD; GSM699631) and castrated (AI; GSM699630) conditions (Fig. 5h). We observed, in LNCaP-AD cells, multiple AR-binding sites (ARBS) across the *BCL-2* locus (Chr18: 63.12–63.32 Mb), some of which were enriched for AR and/or FOXA motifs (Fig. 5h, top). We identified seven prominent ARBS peaks (ARBS1–7), with the ARBS1 representing the main peak located at the *BCL-2* promoter region (Fig. 5h). Strikingly, in LNCaP-AI cells, AR binding was lost at all sites except for a faint residual ARBS1 peak (Fig. 5h). Consistently, most ARBS, especially ARBS1, were reduced or lost in multiple castration-resistant LNCaP sublines (supplementary Fig. 16a). We also observed loss of ARBS1 and other ARBSs in castration-resistant 22Rv1 cells in comparison to parent CWR22 (data not shown).

We performed ChIP-qPCR experiments using primers specific for ARBS1–ARBS3 (Fig. 5i; see Methods and supplementary Table 4) in our paired LNCaP-AD/AI, LAPC4-AD/AI and LAPC9-AD/AI xenograft tumors in which all 3 AI models upregulated BCL-2 (Fig. 1e, f). We found that strikingly, AR binding to ARBS1 in *BCL-2* locus was lost in all 3 distinct AI (i.e., AR^+/hi^ LNCaP-AI, AR^cyto^ LAPC4-AI and AR^-/lo^ LAPC9-AI) models whereas AR binding to ARBS2 and ARBS3 was either lost or reduced (Fig. 5j). These results also corroborated ARBS1 as the major *cis*-regulatory region of BCL-2 by AR. Accompanying the loss of AR binding to ARBS1 (and other ARBSs), RT-qPCR analysis using the same paired AD/AI tumors revealed concurrent *BCL-2* mRNA upregulation, especially in AR^-/lo^ LAPC9-AI tumors (Fig. 5k).

Together, these data suggest that in most CRPC subtypes, AR directly binds and represses the *BCL-2* locus in a ligand-dependent manner and inhibition of AR signaling would block *AR* occupancy at key regulatory regions such as ARBS1 allowing for transcriptional activation of *BCL-2*. To provide further support for this mechanism in patient tumors, we interrogated an integrated dataset (GSE130408)^80^ of AR ChIP-seq in patient Pri-PCa and CRPC and observed globally reduced AR enrichment across the *BCL-2* genomic region in CRPC compared to Pri-PCa (supplementary Fig. 16b–g). We identified a total of 15 AR-binding peaks (except for ARBS6; supplementary Fig. 16c–e) in patient Pri-PCa and CRPC samples all of which except ARBS11 and 12 were reduced in CRPC (supplementary Fig. 16c, g) as confirmed by side-by-side Pri-PCa vs. CRPC comparisons (supplementary Fig. 16f).

### Increased chromatin accessibility in the *BCL-2* genomic region in AR^-/lo^BCL-2^+^ CRPC

We leveraged the aforementioned (Fig. 5c) ATAC-/RNA-seq dataset of 40 patient and organoid CRPC samples^78^ to determine the potential differences in chromatin accessibility in the *BCL-2* locus in AR^+^ vs. AR^-/lo^ CRPC (supplementary Fig. 17). We found that in CRPC_AR tumors, chromatin accessibility across the *BCL-2* genomic region remained low (supplementary Fig. 17a), which coincided with low *BCL-2* transcript levels (supplementary Fig. 17b). In contrast, the AR^-/lo^ CRPC_Wnt and CRPC_NE exhibited increased chromatin accessibility (supplementary Fig. 17a), accompanied by elevated *BCL-2* mRNA expression (supplementary Fig. 17b). Quantitative analysis of ATAC-seq peak intensities across CRPC subtypes revealed a strong positive correlation between *BCL-2* mRNA levels and chromatin accessibility, with the AR^-/lo^ CRPC_NE and CRPC_Wnt exhibiting the highest values for both (supplementary Fig. 17c). Furthermore, *BCL-2* mRNA levels were found to inversely correlate with AR activity (supplementary Fig. 17d). These findings reveal increased chromatin accessibility surrounding the *BCL-2* genomic locus in AR^-/lo^BCL-2^+^ subtype of CRPC and suggest that loss of AR expression and signaling might also promote BCL-2 expression via modulating the chromatin landscape.

### Involvement of AR, ARv7 and GR signaling in driving the BCL-2^-/lo^ phenotype in VCaP-AI

Among the 4 AD/AI models we studied, the VCaP-AD model had the highest ‘baseline’ BCL-2 protein levels (supplementary Fig. 18a) and VCaP-AI was the only model that expressed ARv7 (supplementary Fig. 2 d,f,g, 3b)^20^ and displayed the opposite BCL-2^-/lo^ phenotype (e.g., Fig. 1e, f; supplementary Figs. 2b, h, 3b, c, 12). Like LNCaP-AI and LAPC4-AI, the VCaP-AI tumors showed elevated GR expression (Fig. 1f (note the evident increase in GR protein levels in most samples despite not reaching statistical significance as a group); supplementary Fig. 2c, f–h). Of clinical relevance, VCaP-AI-like cells can be observed in patient CRPC (supplementary Fig. 7d, e). The VCaP model, which constitutively has *AR* genomic amplifications and *TMPRSS2*-ERG fusion and induces ARv7, de novo steroidogenesis and reactivation of AR signaling when progressing to castration resistance, has been used extensively in studying AR signaling and transcriptomic changes during CRPC development.^81–99^

We interrogated and re-analyzed multiple VCaP datasets^81–85,89,93–96^ and performed new studies aiming to shed lights on the BCL-2^-/lo^ phenotype in VCaP-AI (supplementary Fig. 18b–o). Analysis of AR ChIP-seq datasets from several groups revealed, surprisingly, minimal AR occupancy in the *BCL-2* genomic region including ARBS1 in regularly cultured VCaP (VCaP-AD) cells, and this lack of AR binding was not affected by *ERG* knockdown (supplementary Fig. 18c, dataset c1). On the other hand, in VCaP cells cultured in CDSS for 48 h or 4 days, AR binding to several ARBSs, especially ARBS1, was enhanced by DHT or R1881 (supplementary Fig. 18c, datasets c1-c3) but the DHT effect was blunted by Enza pre-treatment (supplementary Fig. 18c, dataset c3). Similarly, DHT stimulated AR binding to ARBS1 in VCaP cells cultured in CDSS for 48 h but not in VCaP cells pretreated with Enza for 3 weeks (VCaP_Enza-3w; supplementary Fig. 18c, dataset c4). Finally, DHT induced prominent AR binding to the *BCL-2* ARBS1 in VCaP cells treated with Enza either short-term (72 h) or long-term (8 weeks in 16 μM Enza; i.e., VCaP16) but not in the same cells with the presence of Enza (supplementary Fig. 18c, dataset c5). These AR ChIP-seq data suggest that the AR protein in VCaP-AD cells was not binding to the *BCL-2* ARBSs, but AR was ligand-responsive and functional. In support, re-analysis of the RNA-seq data in Helminen dataset^94^ (supplementary Fig. 18d–g) revealed DHT-stimulated AR-A in VCaP cells, which was dampened in VCaP_Enza-3w cells (supplementary Fig. 18d). Strikingly, consistent with our data showing reduced BCL-2 protein in VCaP-AI, *BCL-2* mRNA was decreased in VCaP_Enza-3w cells (supplementary Fig. 17e).

We investigated potential involvement of ARv7 in mediating the BCL-2^-/lo^ phenotype in VCaP-AI (supplementary Fig. 18h–m). In mCRPC_SU2C dataset, the *ARv7* mRNA levels positively correlated with *AR* mRNA levels and the (total) AR-A, like in other datasets (supplementary Figs. 14 and 15), also inversely correlated with *BCL-2* mRNA levels (supplementary Fig. 18h, top right panel). Of interest, although *AR* mRNA levels, as expected, strongly correlated with the AR-A, the *ARv7* mRNA levels also exhibited moderate but statistically significant positive correlations with AR-A and negative correlations with *BCL-2* mRNA levels (supplementary Fig. 18h). These results suggest that in ARPI-failed mCRPC, signaling from both full-length AR (AR-FL) and ARv7 contributes to the total AR-A and *BCL-2* repression. In VCaP-CRPC, ARv7 may interact with AR-FL to activate canonical AR target genes and ARv7 may also possess autonomous chromatin-binding and transcriptional capabilities that can sustain AR functional output when AR-FL is lost or its activity inhibited.^86–93,96–99^ Moreover, although ARv7 and AR-FL genomic binding is inter-dependent and co-localized, ARv7 may interact with FOXA1 to preferentially mediate transcriptional repression.^89^ We found that in long-term Enza-treated VCaP16 cells, ARv7 ChIP-seq revealed a subtle but notable increase in ARBS1 binding upon DHT stimulation (supplementary Fig. 18j). Re-analyzing GSE252841 by Poluben et al.,^96^ we observed that siRNAs targeting both *AR* Exon 1 (siEX1, targeting both AR-FL and ARv7) and Exon 7 (siEX7, targeting only AR-FL), but not siRNAs targeting ARv7 (siV7), reduced the *AR* transcript levels (supplementary Fig. 18i, left). However, *KLK3*, the functional readout of AR activity, was reduced by both siV7 and siEX1 but not by AR-FL-specific siEX7 (supplementary Fig. 18i, middle). Strikingly, siV7 induced *BCL-2* in VCaP16 cells (supplementary 18i, right), implicating ARv7 in repressing *BCL-2* in VCaP-AI cells. In support, we exposed our own VCaP-AI cells (derived by culturing VCaP-AD cells in CDSS for 1 week) to an ARv7-selective PROTAC degrader,^100^ which decreased *ARv7* but increased *BCL-2* mRNA levels (supplementary Fig. 18k). Notably, the ARv7 PROTAC completely degraded the ARv7 and dramatically upregulated the BCL-2 protein in our VCaP-AI cells (supplementary Fig. 18l). Interestingly, the siAR^20^ only slightly reduced ARv7 but also significantly upregulated BCL-2 (supplementary Fig. 18l). Finally, we found that in VCaP16 cells where AR-FL was degraded by an AR PROTAC degrader (ARD),^96^ *BCL-2* was repressed rather than induced (supplementary Fig. 18m). These results (supplementary Fig. 18h–m) together indicate that in VCaP-AI cells, ARv7 is a major driver of AR activity and the primary regulator to repress BCL-2 gene expression (especially when AR-FL is lost).

Finally, we analyzed potential roles of GR in regulating *BCL-2* in VCaP-AD/AI systems. We observed that the GR agonist dexamethasone (DEX) increased AR-A in both VCaP and VCaP_Enza-3w cells (supplementary Fig. 18f), consistent with the knowledge that GR, as one of the nuclear steroid hormone receptors sharing DNA-binding sequences with AR, can drive AR transcriptional programs and mediate the so-called ‘bypass’ resistance mechanisms in some PCa settings.^101^ In VCaP-AD cells, DEX induced lower AR-A than DHT (supplementary Fig. 18d, compare condition 6 vs. 2). However, in castration-resistant VCaP_Enza-3w cells, DEX induced a more pronounced increase in AR-A than DHT (supplementary Fig. 18d, compare conditions 8 vs. 4). Interestingly, DEX upregulated *BCL-2* mRNA in VCaP but not VCaP_Enza-3w cells (supplementary Fig. 18g). Unexpectedly, GR ChIP-seq data revealed little GR binding to the *BCL-2* locus (supplementary Fig. 18n). As GR might gain access to many of its genomic targets via interacting with FOXA1,^84^ we examined FOXA1 ChIP-seq data and found that FOXA1 bound to several ARBSs (around ARBS4-7) of the *BCL-2* region in VCaP cells cultured in CDSS for 24 h or 48 h but not in VCaP_Enza-3w cells (supplementary Fig. 18o). Notably, in CDSS-cultured (but not Enza-3w) VCaP cells, DHT, but not DEX, significantly increased FOXA1 binding to the *BCL-2* ARBS1 (supplementary Fig. 18o).

Our results, taken together, suggest the following potential mechanisms for BCL-2 regulation in the VCaP-AD/AI models (supplementary Fig. 18p, q). In the majority of Pri-PCa cells, which have the (AR^+^)BCL-2^-^ phenotype (Fig. 2b, f; supplementary Figs. 4d, 5b), FOXA1 is in equilibrium with AR and ‘pioneers’ the chromatin to facilitate AR co-occupancy at FKHD (Forkhead Domain) and ARE (Androgen Response Element) sites^102^ to repress *BCL-2* transcription (supplementary Fig. 18q, scenario q1). In VCaP-AD cells, which have an AR^+^BCL-2^+^ phenotype, the major ARBS1 of the *BCL-2* locus has FOXA motif (Fig. 5h) but lacks ERG motif (data not shown; see “Methods”). Therefore, there lacks appreciable AR binding to ARBS1 (which is not impacted by ERG knockdown; supplementary Fig. 18c) leading to a lack of AR-mediated *BCL-2* repression. Meanwhile, FOXA1 binds to FKHD at the *BCL-2* ARBS1 and promotes GR loading and GR-mediated *BCL-2* transcription (supplementary Fig. 18g, left; supplementary Fig. 18p, left; supplementary Fig. 18q, scenario q2) leading to high baseline BCL-2 expression (supplementary Fig. 18a). In VCaP-AI, characterized by intracrine androgen production, ARv7 induction, and reactivation of AR signaling (supplementary Fig. 18p, right), several intertwined mechanisms may lead to *BCL-2* repression and the BCL-2^-/lo^ phenotype. Primarily, ARv7, induced de novo and being largely nuclear (due to its ligand-independent nuclear import),^91,97^ may interact with FOXA1 and co-occupy the ARBS1 (e.g., supplementary Fig. 18j, VCaP16_DHT-4 h; supplementary Fig. 18o) where ARv7 functions as a corepressor (CoR)^89^ to suppress *BCL-2* transcription (supplementary Fig. 18q, scenario q3, left). Meanwhile, AR-FL in VCaP-AI cells becomes mostly cytosolic, but as ARv7 levels rise, ARv7 forms heterodimers with AR-FL, pulling AR-FL back into the nucleus and stabilizing it on chromatin.^89,96–99^ Together, the ARv7/AR-FL complex, promoted by intracrine androgens, co-occupies the ARBS1 (and other ARBSs) of the *BCL-2* locus (e.g., supplementary Fig. 18c, DHT conditions) leading to *BCL-2* transcriptional repression (supplementary Fig. 18q, scenario q3). Concurrently, ARv7 may also cooperate with AR-FL as a co-activator to promote canonical AR signaling leading to increased AR and PSA expression (Fig. 1f) and the AR^hi^PSA^hi^ phenotype of VCaP-AI tumors (supplementary Fig. 18q, scenario q3, right).

### The AR^cyto^BCL2^+^ (LAPC4-AI) subtype of CRPC is susceptible to BCL-2 inhibition

**Fig. 6:**
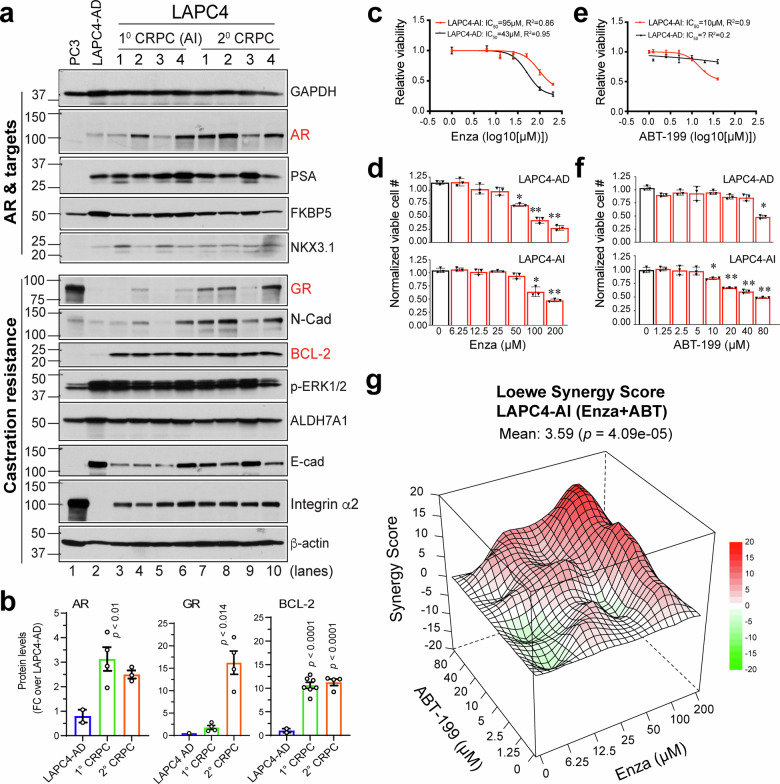
BCL-2 induced in the AR^cyto^ LAPC4-CRPC represents a therapeutic vulnerability. **a** WB analysis of representative regulators in AR signaling and castration resistance in LAPC4-AD and its derived CRPC xenograft tumors, including first-generation (1° CRPC; lanes 3–6) and second-generation (2° CRPC; lanes 7–10) tumors. PC3 and parental LAPC4-AD cells (lanes 1–2) serve as controls. AR, GR and BCL-2 are highlighted in red. (Note part of this panel was re-organized and presented in supplementary Fig. 2e to provide an integrated view of all 4 AD/AI models). **b** Quantification of AR, GR, and BCL-2 protein levels from panel a, normalized to β-actin and shown as fold-change relative to LAPC4-AD (mean ± SD, *n* = 4 tumors/group; *p*-values were calculated using a two-tailed Student's *t*-test). **c**–**f** Dose–response curves showing reduced sensitivity to Enza but increased response to ABT-199 in LAPC4-AI compared to LAPC4-AD in organoids assays, with corresponding IC₅₀ values indicated (**c**, **e**). Cell viability was measured by Resazurin. Data represent the mean ± SD (*n* = 3; *p < 0.05, **p < 0.01; Student’s *t-*test). **g** Loewe synergy analysis evaluating the combined effects of Enza and ABT-199 on LAPC4-AI cells. A 3D synergy surface plot depicts the interaction across increasing concentrations of both agents

**Fig. 7:**
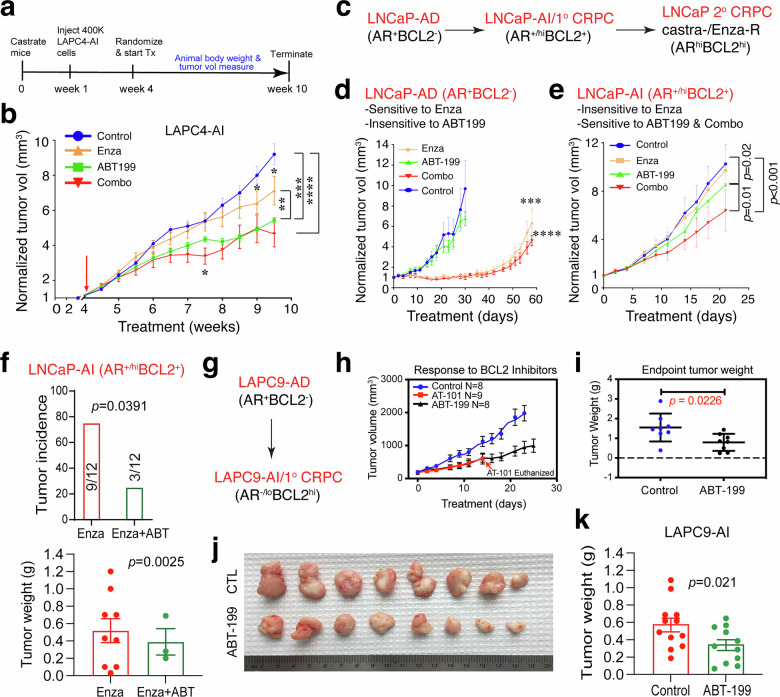
BCL-2 represents a functional therapeutic target across distinct CRPC subtypes. **a**, **b** Experimental schema (**a**) and tumor growth curves of LAPC4-AI xenografts under indicated treatments (*n* = 8 mice/group) (**b**). Presented are the tumor volumes normalized to the mean tumor volumes at the beginning of treatment, i.e., week 4 (mean ± SEM; **p < 0.001, ***p < 0.0001, and ****p < 0.00001; two-way ANOVA). **c**–**f** Therapeutic studies in the progressive LNCaP-AD/LNCaP-AI models (**c**). In vivo tumor growth of LNCaP-AD (**d**) and LNCaP-AI (**e**) xenografts treated with vehicle (control), Enza, ABT-199, or Enza + ABT-199 (Combo). The results showed that LNCaP-AD tumors were sensitive to Enza but not ABT-199 (**d**) while LNCaP-AI tumors are resistant to Enza but sensitive to ABT-199 and the combination (**e**). Tumor volume was normalized to baseline (*n* = 10 per group; mean ± SEM; *p*-values determined by two-way ANOVA). Shown in (**f**) is an independent therapeutic study showing significant inhibition of tumor incidence (top) and weight (bottom) in LNCaP-AI tumors treated with Enza and ABT-199 (ABT) combination compared to Enza monotherapy (tumor incidence and endpoint weight were determined by Fisher’s exact test and unpaired Student’s *t*-test, respectively). **g**–**k** Therapeutic studies in the LAPC9-AI model. Shown are tumor growth curves of LAPC9-AI xenografts treated with ABT-199 (**h**; note the systemic toxicities of AT-101), endpoint tumor weight (**i**) and images (**j**), and an independent ABT-199 monotherapy study in LAPC9-AI xenografts (*n* = 8 mice/group; p-value determined by Student’s *t-*test)

Hereafter, we extended our study to address whether BCL-2 plays a functional role in CRPC progression and may represent a therapeutic vulnerability in diverse subtypes of CRPC (Figs. 6 and 7; supplementary Figs. 19 and 20). We started with the LAPC4-AI model that represented the AR^cyto^BCL-2^+^ CRPC subtype, as shown by castration-induced AR^cyto^ phenotype in vivo (Fig. 3f, g; supplementary Fig. 2b) and in vitro (Fig. 4; supplementary Fig. 13) and whose clinical relevance is supported by presence of AR^cyto^BCL-2^+^ cells in patient CRPC (e.g., supplementary Fig. 7b, d). WB revealed that the LAPC4 1° CRPC and, particularly, the 2° CRPC upregulated AR and GR as well as BCL-2 (Figs. 1e, f and 6a, b; supplementary Fig. 2c, e). We first conducted drug sensitivity assays in organoids derived from LAPC4-AD and LAPC4-AI tumors (Fig. 6c–g; supplementary Fig. 19), which manifested the AR^+^BCL-2^-^ and AR^cyto^BCL-2^+^ phenotypes, respectively (Fig. 3e–g). Under optimized assay conditions (supplementary Fig. 19a–c), the LAPC4-AD organoids were more sensitive to Enza than LAPC4-AI organoids (Fig. 6c, d; IC_50_ 43 μM vs. 95 μM, respectively; *p* = 0.0286, Mann–Whitney U test). In contrast, the BCL-2 inhibitor (BCL-2i) ABT-199 elicited selective toxicity to LAPC4-AI organoids (Fig. 6e, f; IC_50_ ~ 10 μM) but barely showed any inhibitory effect on LAPC4-AD (Fig. 6e, f; IC_50_ not reached) that lacked BCL-2 expression. Also, combination of Enza and ABT-199 synergistically inhibited the LAPC4-AI (Fig. 6g) but not LAPC4-AD (supplementary Fig. 19d) tumor organoids. Interestingly, RU486, a GR antagonist, although showing overall similar toxicities against LAPC4-AD and LAPC4-AI organoids when used alone (supplementary Fig. 19e, f), exhibited synergistic inhibitory effects on LAPC4-AI organoids when combined with Enza (supplementary Fig. 19g).

We then performed in vivo therapeutic studies in castrated male NOD/SCID mice bearing LAPC4-AI tumors (Fig. 7a; supplementary Fig. 20a, b). The results revealed that although Enza modestly inhibited LAPC4-AI, ABT-199 exhibited strong single-agent tumor-inhibitory effects on AR^cyto^BCL-2^+^ LAPC4-AI tumors whereas the ABT-199/Enza combination demonstrated slightly better tumor-controlling effects than ABT-199 alone (Fig. 7b; supplementary Fig. 20c, d).

### The AR^+/hi^BCL-2^+^ (LNCaP-AI) subtype of CRPC is similarly sensitive to BCL-2 inhibition

Next, we examined the sensitivity of AR^+/hi^BCL-2^+^ subtype of CRPC to ABT-199 using the progressive LNCaP-AD → 1° CRPC (AI) → 2° CRPC (castration/Enza-resistant) models (Fig. 7c; supplementary Figs. 2f, g, 10b–e). We first established castration- and castration/Enza-resistant LNCaP cell models that showed similar dynamic changes in AR and BCL-2 to those in the in vivo models (supplementary Fig. 20e–g). Briefly, cells were cultured in CDSS-containing media for 8 weeks to generate castration-resistant LNCaP-CR cells, which were then cultured in CDSS medium containing Enza (20 µM) for 2 weeks to derive LNCaP-C/Enza-R cells (supplementary Fig. 20e). Immunoblot analysis showed a reduction in AR protein in LNCaP-CR cells, followed by AR re-expression in LNCaP-C/Enza-R cells; in contrast, BCL-2 expression progressively increased from the LNCaP-CR to LNCaP-C/Enza-R state (supplementary Fig. 20f, g). LNCaP-C/Enza-R cells were treated with increasing doses of ABT-199 (supplementary Fig. 20h) and viability assays demonstrated higher sensitivity in LNCaP-C/Enz-R cells to BCL-2 inhibition across all doses compared to LNCaP-AD cells (supplementary Fig. 20i).

In vivo therapeutic studies also revealed strikingly different responses based on androgen dependency: the AR^+^BCL-2^-^ LNCaP-AD tumors responded to Enza but not ABT-199 and the combination treatment resulted in similar tumor-controlling effects to Enza alone (Fig. 7d) while the AR^+/hi^BCL-2^+^ LNCaP-AI tumors responded well to ABT-199 and the combination treatment resulted in more pronounced tumor-inhibitory effects than ABT-199 alone (Fig. 7e). In an independent therapeutic study, the ABT-199/Enza combination more prominently inhibited both the incidence and endpoint weights of the LNCaP-AI tumors compared to Enza alone (Fig. 7f).

### The AR^-/lo^BCL-2^+^ (LAPC9-AI) subtype of CRPC is also vulnerable to BCL-2 inhibition

Finally, we asked whether the AR^-/lo^BCL-2^+^ LAPC9-AI (Fig. 7g; supplementary Fig. 11), which was refractory to Enza^20^ (supplementary Fig. 9b), may also be sensitive to BCL-2 inhibition. To this end, we treated castrated male NOD/SCID mice bearing LAPC9-AI tumors (supplementary Fig. 20j, k) with ABT-199 or as a control, AT-101, an early-generation BCL-2/BCL-xL inhibitor derived from gossypol.^57,58^ We observed that ABT-199 significantly inhibited the growth of LAPC9-AI tumors (Fig. 7h–j) without apparent toxicity (supplementary Fig. 20l). An independent therapeutic study verified the inhibitory effect of ABT-199 on LAPC9-AI (Fig. 7k). AT-101 also inhibited LAPC9-AI growth (Fig. 7h) but it manifested significant systemic toxicity in mice such that the treatment had to be terminated early (supplementary Fig. 20l).

### Correlative studies in a phase Ib clinical trial link BCL-2 inhibition to treatment response

**Fig. 8:**
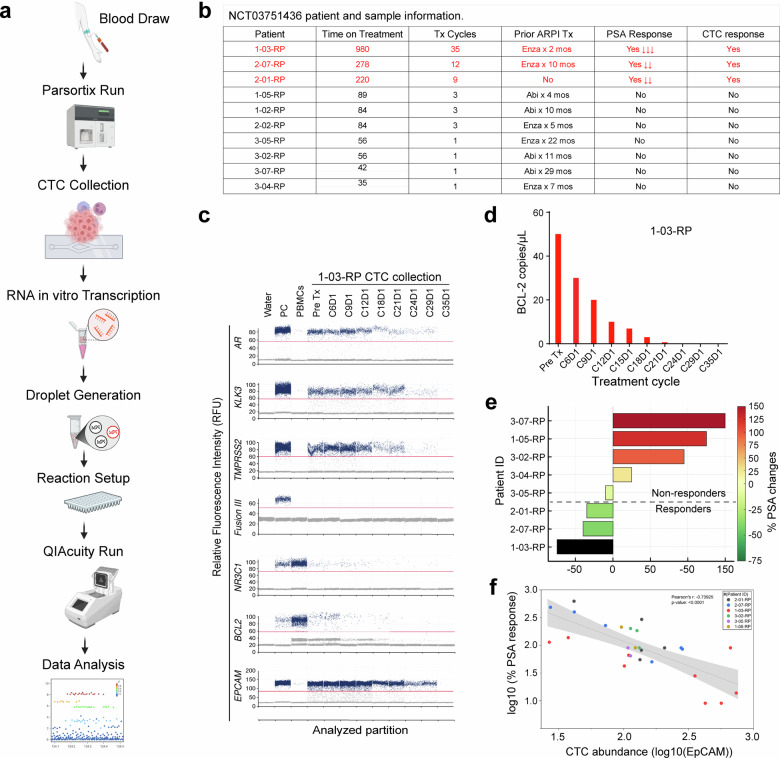
Correlative studies of a Phase Ib trial validates BCL-2 as a target in PCa patients. **a** Schematic workflow. **b** Tabulated summary of the trial patients’ information. **c** One-dimensional ddPCR fluorescence plots demonstrating expression profiles of the indicated target genes across treatment timepoints in the responder 1-03. **d** Quantitative longitudinal analysis of *BCL-2* transcript levels (total copies) in patient 01-03. **e** PSA response stratified by molecular responder vs. non-responder groups. **f** Log-transformed correlation of baseline CTC burden (Log10 EpCAM expression) with PSA response

These preclinical studies revealed the efficacy of BCL-2i ABT-199, either alone or together with Enza, in inhibiting the 3 subtypes (i.e., AR^+/hi^, AR^-/lo^, and AR^cyto^) of BCL-2^+^ CRPC. These results, coupled with our early observations,^20^ led us to conduct a Phase Ib clinical trial^70^ evaluating Enza plus venetoclax (ABT-199) in 10 patients with mCRPC (NCT03751436), and our correlative studies suggested potential therapeutic benefit of this combination in a subset of mCRPC patients (Fig. 8; supplementary Fig. 21).

Longitudinal peripheral blood samples were collected from patients for isolation and transcriptomic profiling of circulating tumor cells (CTCs) using a standardized workflow incorporating microfluidic (Parsortix) enrichment, RNA amplification, and digital droplet PCR (ddPCR) (Fig. 8a, b). This approach enabled high-sensitivity quantification of dynamic changes in *BCL-2* mRNA levels, *AR* expression, AR pathway activity (*KLK3, TMPRSS2*), and potential compensatory or ‘bypass’ survival signaling (e.g., *NR3C1*, i.e., GR) (Fig. 8c; supplementary Fig. 21a, b). CTC collections were also characterized by ddPCR for *TMPRSS2*-ERG fusion (type III) and for relative CTC abundance using the epithelial marker *EPCAM* (Fig. 8c; supplementary Fig. 21a, b). These CTC molecular profiles were then integrated with serum PSA changes, CTC burden, and prior ARPI exposure to identify potential biomarkers of clinical benefit and resistance (Fig. 8b–f; supplementary Fig. 21).

Amongst the 10 trial patients, we observed 3 potential responders, i.e., 1-03, 2-07 and 2-01, all of whom underwent multiple cycles of combination treatment (Fig. 8b, marked in red).^70^ For example, CTC collections from patient 1-03, who was on treatment the longest (35 cycles and 980 days; Fig. 8b), were characterized as *TMPRSS2*-ERG fusion negative and showed treatment-related reductions in *BCL-2* and *AR*, AR activity (*KLK3* and *TMPRASS2*) and overall CTC burden (*EPCAM*) (Fig. 8c). Correspondingly, this patient displayed treatment-related decreases in *BCL-2* copy numbers in CTCs (Fig. 8d) and the most significant drop in serum PSA levels (Fig. 8e; supplementary Fig. 21c). Two additional patients, 02-01 and 02-07, whose CTCs were characterized as *TMPRSS2*-ERG positive (supplementary Fig. 21b), were treated for 9 and 12 cycles, respectively (Fig. 8b) and exhibited some molecular and clinical responses. Late in treatment cycles and towards end of treatment (EOT), the CTCs in both patients showed decreases in *BCL-2* and *AR* expression, AR activity (*KLK3* and *TMPRSS2*), and overall abundance as evidenced by reduced *EPCAM* and the *TMPRSS2*-ERG fusion (supplementary Fig. 21b). Correspondingly, these two patients also had PSA response (Fig. 8e). Nevertheless, earlier treatment discontinuation in both patients was followed by faster PSA rebound (supplementary Fig. 21c) compared to patient 1-03.

In contrast to the above 3 responders, PSA dynamics categorized most other patients as non-responders^70^ (Fig. 8b, e; supplementary Fig. 21c). These non-responders (e.g., 1-05, 3-07, 3-02, 3-04) were generally treated with the Enza/venetoclax combination for only 1–3 cycles (Fig. 8b) and exhibited persistent PSA rise (Fig. 8e; supplementary Fig. 21c). At the molecular level, although the CTCs from the non-responders did exhibit reduced *AR*, *KLK3* and *TMPRSS2-ERG* mRNA levels at EOT, their CTCs showed minimal baseline (pre-Tx) expression of *BCL-2* that did not appreciably change on therapy (supplementary Fig. 21a). Intriguingly, unlike in the 3 responders, the *NR3C1* expression levels in the CTCs of non-responders increased at EOT (compare supplementary Fig. 21a vs. supplementary Fig. 21b and Fig. 8c).

Finally, to assess how CTC levels might be related to treatment response, we performed a correlation analysis between *EPCAM*-based CTC levels and PSA values across all available timepoints from all patients. This longitudinal patient-integrated analysis allowed us to track how CTC abundance evolved in relation to PSA dynamics (i.e., treatment response) over time. The results revealed a strong negative correlation (r = –0.76, *p* = 0.001) between the two (Fig. 8f), suggesting that decreased CTC abundance is closely associated with PSA response during treatment.

## Discussion

PCa is inherently heterogeneous, with treatment-naïve tumors already harboring both ARPI-sensitive AR^+^ cells and ARPI-refractory AR^-/lo^ cell populations.^5,8–18,20^ The widespread clinical use of potent second-generation ARPIs has further expanded AR^-/lo^ foci, which now comprise up to 20–30% of metastatic CRPC cases.^5–8,19,21^ Yet, how these phenotypically distinct cell and mCRPC subtypes adapt to survive AR-targeted therapies—and whether they share common, targetable resistance programs—remains poorly understood. Building on our prior classification^20^ of CRPC into three phenotypically distinct subtypes—AR^+/hi^, AR^-/lo^, and AR^cyto^ (i.e., with AR primarily localized in the cytoplasm and 10–15% AR in the nucleus;^20^ also see supplementary Fig. 2b)—and the emerging recognition of BCL-2 as a survival factor driving castration resistance, here we sought to define how AR^+/-^BCL-2^+/-^ PCa cell subtypes dynamically evolve in response to castration and during progression to CRPC, understand how AR signaling controls BCL-2 expression in different androgen signaling environments, and, critically, determine whether BCL-2 represents a shared, therapeutically actionable resistance mechanism across distinct CRPC subtypes.

Our high-dimensional single-cell imaging analysis across benign prostate (*n* = 123), primary tumors (*n* = 125), CRPC specimens (*n* = 25), and matched xenograft models reveals that the BCL-2⁺ cell subpopulations dynamically evolve and are substantially induced by castration and ARPIs across the PCa continuum. In benign prostate, AR and BCL-2 occupy distinct epithelial compartments—luminal and basal, respectively, reinforcing lineage segregation. Primary tumors remain largely dominated by AR⁺BCL-2⁻ luminal cells, with minimal detection of BCL-2^+^ PCa cells. This is consistent with the known loss of basal cells during prostate tumorigenesis and with the concept that the cell(s) of origin for prostatic adenocarcinomas are likely (AR^+^) luminal progenitor cells rather than (AR^-^) basal cells.^5,103^ However, this balance is disrupted by castration, which induces the evolution and selective expansion of BCL-2⁺ clones across AR^-/lo^, AR^cyto^, and even AR^+/hi^ PCa cell populations. Our qmIF analysis reveals that remarkably, the AR^-^BCL-2^+^ cells could constitute as much as 50% of the 4 cell subpopulations (AR^-^BCL-2^+^, AR^+^BCL-2^-^, AR^-^BCL-2^-^ and AR^+^BCL-2^+^) among the CK^+^ CRPC cells followed by AR^+^BCL-2^+^ cells (Fig. 2d, f). The observations that CRPC is greatly enriched in cells with the AR^-^BCL-2^+^ phenotype, which characterizes normal prostatic basal cells, are fully consistent with our recent findings that ARPIs induce PCa plasticity by concurrently upregulating PCa cells with basal, mesenchymal, neural and stem cell gene expression signatures.^8^ Thus, we speculate that most AR^-^BCL-2^+^ CRPC cells are derived from ARPI-induced reprogramming^2–8^ of AR^+^BCL-2^-^ cells that dominate the primary tumors, although pre-existing ‘basal-like’ PCSCs^14,15^ may also preferentially survive ARPIs and other therapies and therefore become selected for and expanded contributing to increased AR^-^BCL-2^+^ cell population in CRPC.^5,8^

Our data demonstrate that of the 5 prosurvival BCL-2 family members, only *BCL-2* mRNA levels *consistently* anti-correlate with the AR-A in all clinical datasets we have interrogated and only BCL-2 is *consistently* induced by castration (e.g., in 3 of the 4 xenograft AI models) (Fig. 1, Fig. 5a–c; supplementary Figs. 14 and 15). These results suggest that BCL-2, unlike other family members, plays a more general and pivotal role in promoting castration resistance and survival of castrated PCa cells as reported in many previous studies (see Introduction). On the other hand, other prosurvival BCL-2 family members may also be involved in promoting ARPI resistance in different contexts.^104–107^ For example, of the 4 (subtypes of) CRPC models, BCL-xL is upregulated in VCaP-AI (Fig. 1e, f; supplementary Fig. 3), thus implicating BCL-xL in castration resistance in VCaP-AI-like PCa cells/clones. This would be consistent with the reported BCL-xL upregulation in some hormone-refractory models,^104^ the therapeutic efficacy of BCL-xL-targeting BH3 mimetics in RB1-deficient cancers,^106^ and the synergistic effects of co-targeting BCL-xL and MCL1 in inhibiting ARv7-positive CRPC.^107^ Interestingly, unlike the *BCL-2* mRNA levels that consistently correlate, *negatively*, with the AR-A, *BCL-xL* mRNA levels *positively* correlate with the AR-A in 3 (i.e., Rajan, Sharma and Tang F) datasets (supplementary Figs. 14 and 15). Also, there were no apparent *BCL-xL* mRNA changes in the scRNA-seq study (supplementary Fig. 1e) and no apparent BCL-xL protein changes in 3 of the 4 CRPC models except VCaP-AI (Fig. 1e, f). These results, altogether, indicate that BCL-xL likely promotes CRPC in a very context-restricted fashion, i.e., in CRPC with ARv7 induction and/or with RB1 deficiency.^106,107^ This context-related involvement in CRPC may also apply to MCL1, whose mRNA levels, like BCL-2, anti-correlated with the AR-A in 3 (Rajan, Sharma and Long) datasets (supplementary Fig. 14) and which has also been reported as therapeutic target in ARv7-expressing CRPC.^107^ However, unlike BCL-2, MCL1 protein levels were downregulated in our LAPC9-AI, LNCaP-AI and perhaps VCaP-AI models (Fig. 1e, f). Similarly, BCL-W protein levels were decreased in 3 of the 4 xenograft AI models (except VCaP-AI; Fig. 1e, f) but *BCL-W* mRNA levels are positively correlated with AR-A in 3 (Rajan, Nastiuk, Westbrook and Tang F) datasets (supplementary Figs. 14 and 15). Future work will employ our unique AD/AI models to investigate the model-specific and context-dependent roles of these BCL-2 family members during AD → AI transition and in CRPC maintenance and progression.

Regardless, our high-content analysis of AR^+/-^BCL-2^+/-^ cell subpopulation dynamics allowed us to pinpoint three BCL-2-expressing, i.e., AR^-^BCL-2^+^, AR^+^BCL-2^+^ and AR^cyto^BCL-2^+^, cell populations in patient CRPC that, remarkably, are respectively modeled by our unique LAPC9-AI, LNCaP-AI and LAPC4-AI xenograft systems. Consequently, upon establishing a *consistent* inverse relationship between AR activity and *BCL-2* mRNA levels in clinical cohorts and our xenograft AI models, we demonstrate that AR-mediated transcriptional repression of *BCL-2* represents the major mechanism mediating BCL-2 upregulation in these subtypes of CRPC. Specifically, we show that AR directly represses *BCL-2* transcription via binding to multiple ARBS across the *BCL-2* genomic region, especially the ARBS1 around the promoter, and castration and ARPI treatment block AR engagement to most ARBS leading to de-repression of *BCL-2* transcription. Thus, in AR^-/lo^BCL-2^+/hi^ LAPC9-AI subtype of CRPC, loss of AR expression leads to loss of AR activity (supplementary Fig. 15d), loss of AR genomic binding to the *BCL-2* ARBS1-3 (Fig. 5j) and substantially increased *BCL-2* mRNA levels (Fig. 5k; supplementary Fig. 15d). In AR^+^BCL-2^+^ LNCaP-AI subtype of CRPC, although AR is expressed, the AR activity is downregulated^20^ (Fig. 1f; supplementary Fig. 15b) suggesting that AR in such double-positive CRPC cells is not engaging its ‘canonical’ targets like PSA and BCL-2. Indeed, ChIP-qPCR reveals complete loss of AR binding to the *BCL-2* ARBS1 and reduced AR binding to ARBS3 (Fig. 5j) accompanied by concurrent upregulation of *BCL-2* mRNA (Fig. 5k) in LNCaP-AI tumors. In AR^cyto^BCL-2^+^ LAPC4-AI subtype of CRPC, which can be modeled in cultured cells (Fig. 4; supplementary Fig. 13), with the majority of AR sequestered in the cytoplasm^20^ (supplementary Fig. 2b), it’s not surprising that LAPC4-AI tumors exhibit barely detectable AR binding to the *BCL-2* ARBS1-3 (Fig. 5j) and significantly increased *BCL-2* mRNA expression (Fig. 5k). *Finally*, it should be noted that CRPC-NE (i.e., CRPC with an NE phenotype caused by ARPI-induced lineage plasticity) may express the highest levels of BCL-2, which could be associated with loss of AR expression and AR activity (Fig. 5c; supplementary Fig. 15a) as well as direct upregulation by master NE regulators such as ASCL1.^49^

In addition to AR-mediated *BCL-2* repression as the primary driver, other mechanisms may also be involved in upregulating BCL-2 in CRPC. For example, compared to CRPC_AR subtype, the AR^-/lo^ subtypes of CRPC, i.e., CRPC_Wnt and CRPC_NE, manifest notably more open chromatin regions (i.e., ATAC peaks) across the *BCL-2* genomic region in association with higher *BCL-2* transcription (supplementary Fig. 17). This integrated analysis of the chromatin landscape and *BCL-2* transcription in well-defined subtypes of CRPC with contrasting AR signaling activity^78^ suggests that the increased BCL-2 expression in AR^-/lo^ CRPC may result from not only a loss of AR-mediated *BCL-2* repression but also active *BCL-2* transcription mediated by other transcription factors such as NF-κB, STAT3 and E2F1^33,36,42^ bound to these newly available chromatin loci. Moreover, our thorough analysis (supplementary Fig. 18) suggests that the AR^+^BCL-2^+^ phenotype in VCaP-AD is likely driven by GR signaling whereas the AR^cyto^BCL-2^-/lo^ phenotype in VCaP-AI is most plausibly engendered by ARv7 with contributions from the AR-FL (supplementary Fig. 18q). Our results, in aggregate, establish, for the first time, a direct transcriptional link between AR signaling and *BCL-2* repression leading to upregulation of BCL-2 in most CRPC subtypes.

Subsequent therapeutic studies demonstrate that the BCL-2i ABT-199 exhibits single-agent efficacy in all 3 BCL-2 overexpressing AI models whereas Enza and ABT-199 combination manifests synergistic potency in inhibiting the AR^+^BCL-2^+^ (i.e., double-positive) LNCaP-AI. Therefore, in AR^-/lo^BCL-2^+/hi^ LAPC9-AI subtype of CRPC that does not respond to Enza,^20^ ABT-199 alone inhibited tumor growth in several independent experiments with superior toxicity profiles than early-generation BCL-2i AT-101 (Fig. 7g–k; supplementary Fig. 20j–l). In AR^+/hi^BCL-2^+^ LNCaP-AI subtype of CRPC, ABT-199 elicited monotherapeutic efficacy while Enza and ABT-199 combination demonstrated stronger inhibitory effects than Enza or BCL-2i alone (Fig. 7d–f). Finally, in AR^cyto^ LAPC4-AI subtype of CRPC where most AR is ‘sequestered’ in the cytosol with only 10–15% of AR in the nucleus, ABT-199 alone displayed tumor-inhibitory effects whereas the combination of ABT-199 and Enza demonstrated synergistic inhibitory efficacy in tumor organoids (Fig. 6g) and slightly better in vivo tumor-controlling effects than ABT-199 alone (Fig. 7b; supplementary Fig. 20c, d). We reason that the partial Enza response in AR^cyto^ LAPC4-AI organoids/tumors is likely mediated by both residual nuclear AR activity and non-genomic cytosolic AR functions. Regardless, these preclinical results functionally validate BCL-2 as a shared therapeutic target in diverse subtypes of CRPC.

Building on these mechanistic insights and therapeutic outcomes in defined CRPC models, we conducted a Phase Ib trial evaluating Enza combined with venetoclax in mCRPC.^70^ Although the regimen was well tolerated, clinical activity was overall modest, likely due to Enza-induced CYP3A4 that accelerated venetoclax clearance and limited its therapeutic exposure.^70^ Nevertheless, PSA responses together with molecular changes in CTCs identified 3 responders (1-03, 2-01, and 2-07), who were all on the combination treatment for multiple cycles and shared several common characteristics. *First*, all 3 patients appeared to have higher baseline (i.e., pre-treatment) *BCL-2* transcript levels in their CTCs than the non-responders, suggesting that the response to BCL-2i in PCa patients, understandably, is dictated by the tumor expression of BCL-2 and future BCL-2i clinical trials should stratify PCa patients on BCL-2 expression status and levels as has been done in breast cancer patients.^108–110^ *Second*, all 3 responders showed concurrent suppression of *AR* and *BCL-2* expression and AR activity with evidence of CTC clearance. *Last*, unlike the non-responders, the CTCs in the 3 responders did not show increased expression of *NR3C1*, which encodes the GR protein. GR has been well reported to drive castration resistance^20,101^ and indeed, our studies here indicate a synergistic effect of GR antagonist and Enza in suppressing the AR^cyto^BCL-2^+^ LAPC4-AI organoids (supplementary Fig. 19g).

By integrating high-dimensional imaging analysis at single-cell resolution, in-depth mechanistic dissection, extensive therapeutic studies in defined preclinical models, and CTC-based correlative studies associated with a Phase Ib clinical trial, this investigation highlights therapy-induced cellular diversification, exposes the 3 BCL-2-upreulated CRPC subtypes with varying AR expression and signaling intensity (i.e., AR^+^BCL-2^+^, AR^-/lo^BCL-2^+^, and AR^cyto^BCL-2^+^), advances our mechanistic understanding of AR-mediated repression and ARPI-triggered activation of BCL-2 transcription, and, critically, validates BCL-2 as a therapeutic target across distinct CRPC subtypes. On the other hand, our study has several limitations and raises some important questions. *First*, lack of serial samples at progressive stages of castration resistance in our imaging analysis precluded us from obtaining a holistic picture of which subpopulation(s) of AR^+/-^BCL-2^+/-^ PCa cells emerge first and how these subpopulations inter-convert and transition during full CRPC development. *Second*, we provided evidence that the AR^-/lo^ subtype of CRPC (such as LAPC9-AI and CRPC_Wnt and CRPC_NE) may upregulate BCL-2 not only through the loss of AR-mediated repression but also through increased chromatin accessibility, but little is known about what TFs might be actively transcribing the *BCL-2* via these open chromatin loci in AR^-/lo^ CRPC cells. *Third*, the AR^cyto^BCL-2^+^ CRPC cells, which can be recapitulated in the LAPC4-AI model both in vivo and in vitro, clearly exist in patient CRPC. It will be interesting to investigate whether persistent BCL-2 expression in AR^cyto^ CRPC cells is caused by, in addition to the loss of AR-mediated BCL-2 repression, signaling functions of cytoplasmic AR and/or by other nuclear TFs such as NF-κB, STAT33 and E2F1.^33,36,42^ *Fourth*, the VCaP-AI model presents an AR^cyto^BCL-2^-^ phenotype driven by ARv7 (supplementary Fig. 18q). It remains to be determined whether the combined inhibition of AR/ARv7 and BCL-2 could delay or prevent VCaP-AD progression to the castration-resistant stage. As BCL-xL is uniquely upregulated in VCaP-AI, would co-targeting AR/ARv7 and BCL-xL elicit synergistic therapeutic efficacy in VCaP-AI subtype of CRPC? *Finally*, future correlative studies of clinical trials should strive to have paired tumor biopsies—ideally collected longitudinally during ARPI treatment and progression, which will help define how ETS fusion status, AR activity, and BCL-2 expression interact in patients.

Ultimately, these findings should facilitate the translation of BCL-2i to the clinic to treat PCa patients. Our CTC-based correlative studies demonstrate that effective therapeutic responses to the ARPI/BCL-2i combination in mCRPC patients may require concurrent suppression of AR and BCL-2 and clearance of CTCs whereas treatment resistance is marked by persistent CTC burden and associated with activation of alternative survival pathways such as GR. Looking forward, precision stratification of PCa patients based on AR and BCL-2 status could optimize therapeutic efficacy. Integration of liquid biopsy transcriptomics can identify patients with BCL2-driven disease. Additionally, substituting Enza with agents such as abiraterone—an AR inhibitor with strong CYP3A4 inhibition instead of CYP3A4 induction—may enhance BCL-2i exposure and drive more complete clinical benefit in PCa patients receiving the ARPI/BCL-2i treatment.

## Methods

### Cell lines, cell culture, xenograft lines, animals, and animal protocols

LNCaP, LAPC4 and VCaP cells were purchased from the American Type Culture Collection (ATCC) and cultured in RPMI-1640 medium (for LNCaP) or DMEM (for LAPC4 and VCaP) plus 10% heat-inactivated fetal bovine serum (FBS) and antibiotics. LAPC4 and VCaP cells were cultivated in poly-L-lysine-coated plates. LAPC4 and LAPC9 xenograft lines were initially provided by Dr. Robert Reiter (UCLA) and have been used extensively in our previous studies. These PCa cell and xenograft lines were authenticated regularly in our institutional CCSG Cell Line Characterization Core and examined to be free of mycoplasma contamination. Immunodeficient NOD/SCID (non-obese diabetic severe combined immunodeficiency) and NOD/SCID-IL2Rγ^-/-^ (NSG) mice were obtained from the Jackson Laboratory, and the breeding colonies were maintained under standard conditions in our animal core. All animal-related studies in this study have been approved by our Institutional Animal Care and Use Committee (IACUC) at Roswell Park Comprehensive Cancer Center (animal protocol# 1328M, 1331M).

### Establishment of AD and AI (CRPC) xenografts

Briefly, AD (i.e., androgen-dependent) xenograft tumors, LNCaP, VCaP, LAPC4, and LAPC9, were routinely maintained in intact immunodeficient NOD/SCID or NSG mice. To establish the castration-resistant or androgen-independent (AI) xenograft lines, parental AD tumor cells were purified, mixed with Matrigel, injected subcutaneously and serially passaged in surgically castrated male immunodeficient mice. The LAPC4 and LAPC9 AD/AI xenograft lines were maintained in intact and castrated male NOD/SCID mice whereas LNCaP and VCaP AD/AI xenograft lines were passaged in intact and castrated male NSG mice, respectively. Xenograft tumors that became castration-resistant were termed primary (1°) CRPC (or AI) and the AI tumors that became Enza-resistant were termed secondary (2°) CRPC.^20^

To purify human PCa cells, xenograft tumors were harvested, chopped into small pieces (~ 1 mm^3^) and digested with Accutase (Sigma, A6964) for 30 min at room temperature (RT) under constant rotations. Single cells were collected via a pre-wetted 40-μm strainer and further purified on Histopaque-1077 (Sigma) gradient to deplete debris and dead cells.

### Antibodies

Multiple antibodies were used in Vectra-based qmIF (quantitative multiplex immunofluorescence), imaging mass cytometry (IMC), regular Western blotting (WB), quantitative WB (WES), immunofluorescence (IF) and immunohistochemistry (IHC) studies. The basic information for these antibodies is summarized in supplementary Table 2. Main antibodies used in Vectra qmIF analysis included AR, BCL-2 and pan-Cytokeratin. The antibodies used in IMC studies were conjugated to metals using the MaxPar X8 Multimetal Labeling Kit (Fluidigm) according to the manufacturer’s instructions. Before testing antibodies, manufacturer’s website and antibody databases were used for choosing antibodies for targets of interest. Antibodies were initially tested using unconjugated antibodies in regular IF staining of lymph node, spleen, and breast cancer sections. Antibodies that revealed expression patterns consistent with the literature were chosen for metal conjugation. After the conjugation, another round of testing was undertaken using IMC with breast cancer sections and antibodies that showed staining patterns consistent with the literature and with sufficient signal intensity were utilized.

### Primary human PCa (HPCa) and CRPC whole-mount (WM) tissue sections and CRPC tissue microarrays (TMAs) used in the study

In our imaging analyses, we utilized a total of 123 benign prostate tissues, 125 primary HPCa, and 25 CRPC specimens (supplementary Fig. 4a, b). The benign tissues comprised 120 specimens in TMA-1 and TMA-2 as well as 3 WM benign tissues adjacent to the corresponding HPCa; the 125 treatment-naïve HPCa specimens consisted of 121 tumor sections in TMA-1 and TMA-2 and 4 WM HPCa sections; and the 25 CRPC specimens included 20 in TMA-1 and 5 WM CRPC sections (supplementary Fig. 4b). Both TMA-1 and TMA-2 were made in Dr. J. Huang’s laboratory, with TMA-1 consisting of 5 benign prostate tissues, 6 primary tumors, and 20 CRPC (2 cores per sample) and TMA-2 consisting of 115 matched benign and tumor tissues (3 cores/sample). The 3 benign and 4 primary tumor WM sections were made in our lab while the 5 CRPC WM slides were provided by Dr. J. Huang (supplementary Fig. 4b). Formalin-fixed paraffin-embedded (FFPE) sections were cut from these TMAs and WM samples and used for Vectra and IMC studies, and relevant information on patient samples was summarized in supplementary Table 3.

### Chemicals, drugs, key assay kits and reagents, primers, and probes

All relevant information is summarized in supplementary Table 4, along with their sources, catalog numbers, and uses. Compounds were chosen based on their relevance to pathway inhibition, therapeutic targeting, or the experimental design and were reconstituted and stored according to the manufacturers’ instructions. PROTAC AR-V7 degrader-1 (MCE, catalog# HY-145479) is an orally active and selective AR-V7 PROTAC degrader with a DC_50_ of 0.32 μM in 22Rv1 cells (company product sheet).

### Regular western blotting (WB) and automated quantitative WB (WES)

For regular WB, total protein extracts were obtained from cell or tissue samples using Pierce RIPA buffer (ThermoFisher Scientific, USA) according to manufacturer’s protocol. Protein concentrations were determined using Pierce BCA Protein Assay Kit (ThermoFisher Scientific, USA). 40 μg of total protein were separated in Bolt 4-12% Bis-Tris Plus Gels (ThermoFisher Scientific, USA) and transferred to Hybond P 0.45 PVDF membranes. Membranes were blocked with 5% BSA in TBST (FisherSci, USA) and probed overnight at 4 °C with primary antibodies (supplementary Table 2). Secondary antibodies were HRP-conjugated anti-rabbit IgG or anti-mouse IgG (CST 7074, 7076, 1:10,000). Protein bands were revealed using Luminata Classico or Luminata Forte HRP substrate (MilliporeSigma, USA) and detected using Chemidoc Imaging system (Bio-Rad, USA).

In some studies, we also employed the automated the Simple Western WES^TM^ immunoassays^70^ (https://www.bio-techne.com/brands/proteinsimple), which take place in capillaries and separate proteins by size as they migrate through stacking and separation matrix. The separated proteins are immobilized to the capillary wall via a proprietary, photoactivated capture chemistry. The target protein was identified using a primary antibody and immunoprobed using an HRP-conjugated secondary antibody and chemiluminescent substrate. The resulting chemiluminescent signal is displayed as traditional virtual blot-like image and electropherogram. Quantitative results such as M.W, signal intensity (area), % area, and signal-to-noise for each immunodetected protein are presented in the results table automatically.

### Immunofluorescence (IF) in cultured cells

Cells were seeded at 40,000 cells per well in an ibidi 8-well chamber slide and allowed to adhere overnight. The following day, cells were incubated with MitoTracker™ (e.g., 100 nM in complete medium) at 37 °C for 30 min to label mitochondria, followed by gentle washing with warm PBS. Cells were then fixed with 4% paraformaldehyde in PBS for 15 min at RT, permeabilized with 0.1% Triton X-100 in PBS for 10 min and blocked with 5% BSA in PBS for 30 min. For single IF staining, cells were incubated with Alexa Fluor 647-conjugated anti-AR antibody for 1 h at RT or with anti-BCL-2 antibody (supplementary Table 2) for 4 h at RT, followed by appropriate Alexa Fluor-conjugated secondary antibody incubation. For co-staining, cells were sequentially incubated with anti-BCL-2 antibody and secondary antibody, followed by Alexa Fluor 647-conjugated anti-AR antibody. Control wells included a no-antibody condition as isotype controls, where rabbit IgG replaced the AR antibody and normal mouse IgG replaced the BCL-2 antibody. After final PBS washes, nuclei were optionally counterstained with DAPI, and slides were mounted using antifade medium and imaged using Keyence BZX-700 microscope.

### IHC in FFPE sections

The slides were de-paraffinized and rehydrated according to common protocols. Antigen retrieval was performed by boiling the slides in citrate buffer (pH=6.0) for 10 min using microwave. Sections were blocked using solution of 10% FBS (Gibco, USA) and 1% BSA in TBS for 2 h at RT and probed with AR antibody (CST D6F11, 1:100 in TBS with 1% BSA) overnight at 4 °C. Slides were rinsed with 0.0025% Triton-X-100 (Fisher Scientific, USA) in TBS, probed with secondary HRP-conjugated anti-rabbit IgG (CST 7074, 1:1000 in TBS with 1% BSA) for 1 h at RT and developed with 3,3-diaminobenzidine (DAB) kit (Vector Laboratories, USA). Separate slides were counterstained with hematoxylin (Fisher Scientific, USA). After staining, slides were rinsed with DI water, dehydrated, and mounted using toluene (Fisher Scientific, USA). The slides were scanned using Aperio ScanScope imaging system (Aperio Technologies, Vista, CA, USA) and a 40x objective. Images were analyzed using the ScanScope software.

### Vectra-based quantitative multiplex IF (qmIF) staining and data analysis using inForm

qmIF analysis with tyramide signal amplification was performed on FFPE slides of TMAs, WM CRPC patient samples and PCa AD/AI xenografts. The qmIF antibody panel (supplementary Table 2) consisted of AR, BCL-2 and pancytokeratin AE1/AE3 and the FFPE sections (5 μm) stained with the automated Vectra platform. Antibody staining in the qmIF panel was compared to that in single stained slides on control tonsil and prostate tissue. Staining patterns did not differ between the single and multiplex scanned slides. Each marker was also validated with conventional IHC. Slides were scanned and analyzed with the Vectra Polaris (Akoya Biosciences).

After staining, slides were imaged using the Vectra 3.0 automated imaging system (Akoya Biosciences). First, whole-slide scans were made at 10x magnification. Subsequently, multispectral image scan was processed to single images using Phenochart slide viewer (Akoya Biosciences). Multispectral library slides were created by staining a representative sample with each of the specific dyes. The multispectral library slides were unmixed into eight channels using inForm software (version 2.4): DAPI, CK OPAL 480, AR OPAL520, BCL2 OPAL570 and Auto Fluorescence and exported to a multilayered qTIFF file. The multilayered TIFFs were fused with Phenoimager software (version 3.0) to create one file for each sample. Cells were phenotyped as pancytokeratin AE1/AE3 positive (CK^+^ ) or negative (CK^-^) cells. The cell density for each cell subset was further assessed for CK^+^ epithelial compartment and expressed as number of AR^+/-^BCL-2^+/-^ cells/mm^2^.

For Vectra data analysis, we employed the inForm Cell Analysis program, which enabled us to define the biology of interest within a tissue section. It phenotypes cells based on their biomarker expression within cells, nuclei and membranes. *inForm* Tissue Finder added exceptional functionality to inForm Cell Analysis. It automated the detection and segmentation of specific tissue compartments through powerful algorithms. Automation provided consistent reproducible results and enabled comparative studies of multiple markers and multiple specimens.

### Imaging mass cytometry (IMC)

Data acquisition was performed on a Helios time-of-flight mass cytometer (CyTOF) coupled to a Hyperion Imaging System (Fluidigm). Selected areas for ablation were larger than the actual area of interest to account for loss of overlapping areas among sections due to cumulative rotation. The selected area ablated per section was around 1 mm^2^. Laser ablation was performed at a resolution of approximately 1 µm with a frequency of 200 Hz and an estimated acquisition time of 1 mm^2^ h^−1^. To ensure performance stability, the machine was calibrated daily with a tuning slide spiked with five metal elements (Fluidigm). All data were collected using the commercial Fluidigm CyTOF software v.01.

For antibody validation, the AR, CK antibodies were purchased from Fluidigm R (https://www.fluidigm.com). The carrier-free BCL-2 antibody was conjugated Nd146 using the Maxpar X8 metal conjugation kit following manufacturer’s protocol (Fluidigm 201300). Post-conjugation, antibody specificities were again tested using immunofluorescent staining, followed by titration in the IMC platform.

For antibody staining and image acquisition, 2–4 μm FFPE sections were stained with an antibody cocktail (supplementary Table 2) containing all (AR, BCL-2, Pan CK) antibodies. Briefly, tissue sections were de-paraffinized with xylene and subjected to sequential rehydration from 100% ethanol to 70% ethanol before being transferred to PBS. Heat-induced antigen retrieval was performed at 95 °C for 30 min in Tris/EDTA buffer (10 mM Tris, 1 mM EDTA, pH 9.2). Slides were cooled to RT and subsequently blocked with PBS + 3%BSA for 1 h at RT. Meanwhile, the antibody cocktail was prepared in PBS + 1%BSA buffer, with appropriate dilutions for each of the antibodies (supplementary Table 2). Each slide was incubated with 100 μl of the antibody cocktail overnight at 4°C. The next day, slides were washed 3 times with PBS and labeled with 1:400 dilution of Intercalator-Ir (Fluidigm 201192B) in PBS for 30 min at RT. Slides were briefly washed with H2O three times and air dried for at least 30 min before IMC acquisition. All IMC operation was performed following Fluidigm’s operation procedure. Briefly, following daily tuning of IMC, image acquisition was carried out following manufacturer’s instruction at a laser frequency of 200 Hz. 1000 μm × 1000 μm regions around islets were selected based on bright field images.

For IMC data analysis, the CellProfiler (version 3.0.0) analysis pipeline was used to run complex image on batches of several hundreds of images. The software requires the use of analysis modules which run image processing algorithms, threshold-based segmentation, calculations, and file processing (https://star-protocols.cell.com/protocols/1361). To quantify each marker, we developed an IMC segmentation and analysis pipeline based on the ‘IMC Segmentation Pipeline’ repository created by the Bodenmiller group.^111^ Cell Profiler was used to prepare the images for segmentation based on an overlay of all markers to identify cytoplasm/membrane, nuclei (iridium), and non-cellular space. This step is independent of signal intensity for markers, as it captures overall cellular morphometry. These data were subsequently loaded into HistoCAT for quantification and downstream analysis of single-cell populations. Dimensionality reduction using the tSNE algorithm of HistoCat allowed for visualization of the multiplexed, single-cell dataset in two dimensions. tSNE was selected as it has been most widely used for clustering of IMC data. Comparison of AD and AI tumors via tSNE highlighted global differences in the distribution and grouping of single-cell data. A heatmap plot visualizing relative signal intensity per cell by individual markers enabled phenotypic classification of the four AR^+/-^BCL-2^+/-^ cell meta-clusters. If a panel of slides is stained using IMC for a discrete marker, the intensity of that marker across cells in an individual section and across different ROIs obtained from the same type of tissue, stained using the same method, can be measured and compared.

### Establishment of castration-resistant or castration/Enza-resistant LAPC4, LNCaP, VCaP cells

We established the castration-resistant and dual castration/Enza-resistant LAPC4 cell models (Fig. 4; supplementary Fig. 13) to further study the AR^cyto^ subtype of CRPC and its therapeutic responses. To this end, we cultured regular LAPC4-AD cells long-term (4 months) in charcoal dextran stripped serum (CDSS) medium to develop castration-resistant LAPC4 (LAPC4-CR or LAPC4-AI) cells, which were subsequently exposed to either 20 μM or 100 μM Enza-containing CDSS medium for 1 month resulting in the LAPC4-Enza(20)-R or LAPC4-Enza(20)-R models (supplementary Fig. 13a).

Analogously, LNCaP cells were cultured in CDSS-containing medium for 8 weeks to generate castration-resistant LNCaP-CR cells, followed by Enza exposure (50 µM, 2 weeks) to derive LNCaP-C/Enza-R cells which were then used, together with LNCaP-AD cells in ABT-199 sensitivity studies (supplementary Fig. 20e–i). Finally, we generated castration-resistant VCaP cells by switching regularly cultured VCaP cells (DMEM + 20% FBS) to CDSS-containing medium for 1 week followed by treatment with the PROTAC ARv7 degrader-1 (0.5 μM, 24 h; supplementary Fig. 18k, l).

### Drug treatment of 2D cell cultures and 3D organoids

Drugs such as enzalutamide (Enza; Apex Bio, A3003) and ABT-199 (Apex Bio, A8194) were dissolved in DMSO. Control samples in all experiments were treated with vehicle (DMSO) only and vehicle concentration in growth medium did not exceed 0.2%. For drug treatment in 2D cultures, cells were seeded in 24-well ultra-low attachment plates (Corning, USA) at 2000 cells/well. After 7 days cell clusters were harvested and disrupted by mild trypsinization, pelleted, and resuspended in PBS. Cell viability was assessed by counting using a Countess automated cell counter (ThermoFisher Scientific, USA) in presence of 0.4% Trypan Blue.

For organoids-based drug assays, xenograft tumor cells were isolated and seeded into 384-well microtiter plates at 10,000 cells per well in F12 advanced DMEM media supplemented with 10% Matrigel. Drugs in the same culture medium were added in half-log dilutions starting at 0.1 μM. Cells were incubated at 37 °C (5% CO_2_) for 6 days and the effects of drugs on organoids viability were determined using Resazurin cell viability assays. Data were normalized to “Control” samples and presented as mean ± SD. Kruskall–Wallis H-test was applied against corresponding concentrations of drugs (e.g., Enza) that yielded the fraction affected (FA) when acted alone and in combination with BCL-2 and AR inhibitors. The drug–drug interaction studies were assessed using Compusyn software https://compusyn.software.informer.com/1.0/ and synergy finder www.synergyfinderplus.org.

### ChIP-qPCR analyses of the AR-binding sites (ARBS) on the *BCL-2* genomic locus

ChIP was performed in LNCaP, LAPC4, and LAPC9 AD/AI xenograft tissue lysates using the ChIP-IT High Sensitivity Kit (Active Motif, #53040), following the manufacturer’s protocol. Chromatin fragments containing the *BCL-2* regulatory regions were immunoprecipitated using ChIP-grade anti-AR antibody (EPR179 # ab108341). An anti–RNA Polymerase II monoclonal antibody and normal IgG, both included in the kit, were used as positive and negative ChIP controls, respectively. Input samples—representing 2% of the total chromatin prior to immunoprecipitation—were processed in parallel and used for normalization. After reverse cross-linking, immunoprecipitated DNA and input samples were analyzed by qPCR using ARBS-specific primers targeting the *BCL-2* locus (supplementary Table 4). IgG pulldown samples served as negative controls to confirm the specificity of enrichment.

To validate AR occupancy at the *BCL-2* locus, we designed primers targeting three high-confidence ARBS (i.e., ARBS1–3) and one negative (Neg) control region based on AR ChIP-seq profiles in androgen-dependent (AD; GSM699631) versus androgen-independent (AI; GSM699630) LNCaP cells (Fig. 5h, i). Candidate peaks were selected using MACS (v1.4.2) peak calling with a stringent p-value cutoff (1e-5) and were visualized on the UCSC Genome Browser (hg38) for cross-comparison with histone marks and enhancer annotations. The ‘Neg’ control region lacking AR binding (chr18:63,300,951–63,301,529) was selected from a neighboring genomic region. The following genomic coordinates (hg38) were used as the qPCR amplicon targets: ARBS1: chr18:63,321,543–63,321,592 (50 bp); ARBS2: chr18:63,202,549–63,202,622 (74 bp); ARBS3: chr18:63,200,006–63,200,051 (46 bp). Primers and probes were designed by Bio-Rad (supplementary Table 4) to specifically amplify ARBS1–3. Probes were labeled with FAM for ARBS1–3 and HEX for Neg control to enable clear discrimination of specific AR-binding events. Primer-probe sets were optimized to amplify peaks corresponding to AR occupancy identified by ChIP-seq.

In brief, 10 × 10⁶ single cells isolated from the AD/AI PCa xenograft tissues were fixed in formaldehyde for 15 min, and chromatin was sheared using the Covaris E210 focused-ultrasonicator for a total of 16 min. Shearing was performed in 12 × 12 mm, 1 mL glass tubes containing a fiber insert (Covaris, cat. no. 520080) placed in a compatible tube holder (Covaris, cat. no. 500276), using the following instrument settings: 20% duty cycle, intensity 8, and 200 cycles per burst, applied in sixteen 1-min treatment cycles. After shearing, 10 μL of the 2 mL sonicated chromatin was reserved as input, and the remaining chromatin was incubated overnight at 4 °C with 4.0 μg of anti-AR antibody or normal rabbit IgG (Cell Signaling Technology, Cat. #2729). For qPCR analysis, 5 μL of immunoprecipitated DNA was used as a template in a 10 μL total reaction volume using TaqMan™ Advanced PCR Master Mix (Thermo Fisher Scientific) and target-specific TaqMan probes, prepared in 384-well plates. Quantitative PCR was performed on a QuantStudio™ 6 Flex Real-Time PCR System (Applied Biosystems) under standard cycling conditions: initial activation at 95 °C for 10 min, followed by 40 cycles of 95 °C for 15 s and 60 °C for 1 min. Data were analyzed by calculating fold enrichment over IgG control.

### Therapeutic experiments in LNCaP-AD/AI, LAPC4-AI and LAPC9-AI xenografts

Basic experimental protocols for in vivo therapeutic studies were described previously.^20^ Briefly, for all AI xenograft studies, NOD/SCID or NSG male mice (8–10 weeks old) were castrated on day 0. After 7–10 days, 250,000 CRPC cells from various AI xenograft models^20^ (i.e., LNCaP-AI, LAPC4-AI and LAPC9-AI) were injected subcutaneously in Matrigel. Once tumors became palpable, mice were randomized into treatment groups, and drugs were administered by oral gavage: ABT-199 at 50–100 mg/kg five times per week and Enza at 30 mg/kg three times per week, for a total treatment of ~3–6 weeks (depending on models). All drugs were dissolved in DMSO and formulated in 10% DMSO, 50% PEG300, and 10% Tween-80. Tumor dimensions were measured weekly using calipers, and volumes were calculated as ½ × (length × width²). Animal body weights and health were monitored throughout the treatment period. At the study endpoint, tumors were harvested for analysis of incidence, weight, and gross morphology.

Specifically, we evaluated the therapeutic efficacy of ABT-199, alone or in combination with Enza in models of distinct CRPC subtypes (Fig. 7, supplementary Fig. 20). In the AR^Cyto^ LAPC4-type CRPC model (Fig. 7b), mice were assigned to four groups: (1) vehicle control (*n* = 12), (2) Enza (30 mg/kg; *n* = 10), (3) ABT-199 (50 mg/kg; *n* = 12), and (4) combination (Enza 30 mg/kg + ABT-199 50 mg/kg; *n* = 10). In the AR^-/lo^ LAPC9-type CRPC (Fig. 7g–j; supplementary Fig. 20j–l), mice were treated with either vehicle control, ABT-199 or AT-101 for up to ~4 weeks. In an independent LAPC9-AI therapeutic study (Fig. 7k), castrated male NOD/SCID mice bearing LAPC9-AI tumors were treated with vehicle control (*n* = 10) or ABT-199 (100 mg/kg; *n* = 12) for 3 weeks. Finally, we conducted several independent therapeutic studies in AR^+^BCL-2^-^ LNCaP-AD and AR^+/hi^BCL-2^+^ LNCaP-AI tumor models (Fig. 7c–f) as previously described.^20^

### RNA isolation and qRT-PCR analysis

Total RNA was extracted from cultured cells or the AD/AI xenograft tumors using RNeasy Mini Kit (Qiagen) according to the manufacturer’s instructions. qRT-PCR of *AR* (e.g., Fig. 4g) and *BCL-2* (e.g., Fig. 5f, g, k) was performed using a CFX Connect Real-Time PCR Detection System (Bio-Rad) and probes presented in supplementary Table 4. qPCR data were normalized to housekeeping gene TBP and/or HPRT1 expression levels.

### Integrated analysis of ATAC-seq, AR (and ARv7, GR, FOXA1) ChIP-seq, and transcriptomic (RNA-seq, scRNA-seq and microarray) data

#### Data acquisition

We sourced ATAC-seq, AR/ARv7/GR/FOXA1 ChIP-seq, RNA-seq and microarray datasets from publicly available repositories, with a focus on benign prostate tissue, Pri-PCa, CRPC, and related PCa cell lines. Dataset sources, accession identifiers, and metadata such as sample type, sample size, and platform are detailed in supplementary Table 1.

#### Transcriptomic data (RNA-seq and microarray)

Normalized gene expression datasets were obtained from the NCBI Gene Expression Omnibus (GEO; RRID:SCR_005012; https://www.ncbi.nlm.nih.gov/geo/), cBioPortal for Cancer Genomics (RRID:SCR_014555; https://www.cbioportal.org/), and UCSC Xena Functional Genomics Portal (RRID:SCR_018938; https://xenabrowser.net/). Cohorts included benign prostate tissues, untreated and treated primary PCa (e.g., pre-/post-nADT, pre-/post-Enza), CRPC, and PCa cell lines.

#### Microarray data processing

Two microarray datasets were included. For GSE89050,^71^ normalized data for FACS-purified basal, luminal, and luminal progenitor cells from benign prostate tissues were directly downloaded from GEO. For GSE21034,^112^ normalized expression values for benign prostate tissues, primary PCa and metastatic PCa tissues were retrieved from cBioPortal. For both datasets, expression values were log₂-transformed for downstream visualization and analysis. No additional normalization steps were applied beyond the original authors’ processing.

#### Bulk RNA-seq data processing

RNA-seq expression values (FPKM, TPM, or DESeq2-normalized counts) were obtained directly from the original data sources. When applicable, fold-change (FC), 95% confidence intervals (CIs), *p*-values, and false discovery rates (FDR) were either extracted from the original publications or computed using DESeq2^113^ (version 1.28.1; RRID:SCR_015687) for datasets with raw counts—including the Sharma pre-/post-nADT cohort (GSE111177), LNCaP-ARKO/AR datasets, and LNCaP-derived secondary CRPC/primary CRPC/AD samples (GSE88752). For dataset without raw counts (i.e., the Alumkal pre-/post-Enzalutamide cohort), fold-changes were calculated from TPM values, and paired comparisons were assessed using the one-sided Wilcoxon signed-rank tests. No additional normalization or batch correction was applied beyond the processing inherent to DESeq2 or the source datasets, unless otherwise specified.

#### scRNA-seq data processing

Reprocessing of publicly available scRNA-seq datasets was performed as previously described.^76^ In brief, BAM files from Cheng et al.^75^ (SRA: PRJNA699369) were converted to FASTQ using bamtofastq and processed with Cell Ranger. Cells were filtered using Loupe Browser v6.1.0 based on UMI counts (512–65,536), detected genes per bar code (1024–8192), and mitochondrial UMIs (< 7.5%), yielding 24,142 QC-passed high-quality cells for downstream analysis. Dimensionality reduction (PCA/UMAP) and K-means clustering (k = 10) were applied, and clusters were annotated into nine epithelial subtypes using sample origin, global gene signatures, and *AMACR* expression.

#### ChIP-Seq data acquisition and analysis

Reads were mapped to the human genome (hg38) using Bowtie (version 1.1.2)^114^ with the following parameters: “-v 2 -m 1 --best --strata”. To avoid PCR bias, only one copy of multiple reads mapped to the same genomic position was retained for further analysis. *Peak Calling:* For each ChIP-Seq sample, peaks were identified using MACS (version 1.4.2)^115^ by comparing against the corresponding input sample. A window size of 300 bp was used, and a *p*-value cutoff of 1e-5 was applied. The peaks that overlapped with ENCODE blacklisted regions^116^ were removed. *Differential peak analysis:* For each differential comparison, peaks from all the involved samples were merged, and the number of reads within these merged peaks was counted for each sample. Merged peaks with less than 10 reads in all samples were removed. The resulting count table was used to identify differential peaks using the R/Bioconductor package edgeR.^117^ The numbers of reads within the common peaks of all samples were used as the library sizes in edgeR. Peaks with a false discovery rate (FDR) ≤ 0.05 and a fold change ≥1.5 or 2 were identified as differential peaks and presented in heatmap plots. *Signal Track:* Each read was extended by 150 bp to its 3’ end. The count of reads covering each genomic position was multiplied by 1 × 10^7^ divided by the library size used in edgeR. These values were then averaged over a 10 bp resolution. The resulting averaged values were displayed using the Integrative Genomics Viewer (IGV).^118^

#### ATAC-Seq data acquisition and analysis

Adapter sequences were removed from the 3’ ends of reads using Trim Galore! (version 0.6.5; Babraham Bioinformatics. https://www.bioinformatics.babraham.ac.uk/projects/trim_galore/) and cutadapt (version 2.8).^119^ The reads were then mapped to the human genome (hg38) using Bowtie (version 1.1.2) with the following parameters: “--allow-contain --maxins 2000 -v 2 -m 1 --best --strata”. To avoid PCR bias, only one copy of multiple fragments mapped to the same genomic position was retained for further analysis. After the removal of fragments from chrM, for each fragment, the 5’ end was offset by +4 bp and the 3’ end was offset by −5 bp to adjust both ends to represent the center of a transposon binding event. *Peak Calling:* For each sample, peaks were identified using MACS2 (version 2.2.7.1) without using any control. Each binding event (i.e., the 5’ or 3’ end of a fragment) was smoothened by 73 bp (i.e., extended 36 bp upstream and 36 bp downstream from the event center). MACS2 was configured to call peaks from the pile-up of smoothened binding events, and a q-value cutoff of 0.05 was applied. The peaks that overlapped with ENCODE blacklisted regions were removed. *Signal Track:* each transposon binding event was smoothened to a length of 73 bp, spanning from −36 bp to +36 bp around the center. The count of binding events covering each genomic position was multiplied by 1 × 10^7^ divided by the total number of binding events. These values were then averaged over a 10 bp resolution. The resulting averaged values were displayed using the Integrative Genomics Viewer (IGV). Pearson’s correlation was used to assess the relationships between chromatin accessibility, AR binding, and *BCL-2* expression.

### Search for potential ETS motifs within the ARBS1 region of the *BCL-2* genomic locus

The central ARBS1 peak of the *BCL-2* locus spans 328 bp (Fig. 5h), with the following sequence: 5’-ATAGCCCCTAGCAAAAAAGGACAAGAGGACAAACAAGTTGCACGTGTGTATTTTTATCTCCAAGAGTCTTTGACAAAGCACCAAGAAAAGAAAAAGGTCTTCTAGAAGCACAGCGGCTTACTTAATAGGGCTCGAGTGCAAATATATAGGGACACTGGATTATTGGGGTCAAGAAAGATGACAAATGAGTACTACAATGTGTACAAATATGCTATGACTTTTTTTTTTTTTTTGAGACAGAGTCTTGCTCTGTTGTATCTTGGAGGCTGGTGTGCAGTGGTGCGATCTCAGCTCACTGCAACCTCTGCCTCCCAGGCTCAAGCAATTC-3’. Motif scanning of the ARBS1 region was performed using FIMO (MEME Suite) with position weight matrices (PWMs) for seven representative ETS family transcription factors (ETS1, ETS2, ERG, ETV1, ETV2, ELK1, and ELK4), obtained from JASPAR 2024 motif database. FIMO was run with default parameters, and motif occurrences were evaluated using the program’s standard significance threshold. No ETS motif occurrences with *p*-value < 1 × 10⁻⁴ were detected, indicating that the ARBS1 region lacks statistically significant canonical ETS-binding motifs under the conditions tested.

### Phase Ib clinical trial of treating mCRPC patients with enzalutamide and venetoclax combination

The NCT03751436 trial was a phase Ib open label single-arm, single-center study of Enza with the BCL-2i venetoclax in patients with mCRPC.^70^ A total of 10 patients were enrolled, starting with a standard dose of Enza (160 mg/d) and 3 dose levels (DL) of venetoclax at 400 mg/d (DL1), 600 mg/d (DL2) and 800 mg/d (DL3). The primary objectives of this phase Ib study were to: (1) characterize the safety and tolerability profile and (2) determine the dose-limiting toxicity (DLT), maximum tolerated dose (MTD) and recommended phase II dose (RP2D) of the Enza and venetoclax combination in patients with mCRPC. We also analyzed the pharmacokinetic (PK) profiles of Enza and venetoclax when given in combination to confirm whether drug levels were in the therapeutic range. For patient accrual, treatment and adverse events, clinical outcomes, and major PK parameters, refer to ref. ^70^.

### Circulating tumor cell (CTC) enrichment and characterization

CTCs were isolated from patient blood samples using the Parsortix system (ANGLE plc’s Parsortix™). Blood was processed according to the manufacturer’s protocol, enabling size- and deformability-based enrichment of CTCs while minimizing contamination by RBCs and WBCs. Following enrichment, the captured CTCs were harvested directly from the Parsortix cassette using ANGLE’s cell harvest protocol. Cell pellets were immediately stored in the extraction buffer provided in the NanoPure RNA extraction kit or processed directly for downstream analysis.

To molecularly characterize the enriched CTC collections, total RNA was extracted using the NanoPure RNA extraction kit, ensuring efficient recovery from low cell numbers. The Arcturus™ RiboAmp™ PLUS RNA Amplification Kit (Thermo Fisher Scientific) was used for linear amplification of low-input RNA from as little as 1–10 cells, enabling molecular analysis from previously challenging quantities. This system amplified RNA from as little as 1 pg of total RNA, producing antisense RNA (aRNA) suitable for digital droplet PCR. To ensure specificity and accuracy of the amplification process, both positive and negative controls were included: positive controls (PC) consisted of RNA from PCa cell lines (LNCaP, PC3 and VCaP) known to express target genes such as AR and negative controls included RNA from healthy donor PBMCs that do not express tumor-specific genes, along with no-reverse transcription (no RT) and water controls. The amplified RNA samples were processed by iCura Diagnostics using the QIAcuity digital PCR system. This approach enabled precise quantification of target transcripts, ensuring accurate representation of the original low-abundance RNA derived from CTCs. The use of digital PCR enhanced both sensitivity and specificity, validating the amplification process and confirming the presence of tumor-specific targets.

The information for ddPCR primers and probes was summarized in supplementary Table 4. The type III TMPRSS2-ERG fusion was detected using the published information.^120^

### Ethics statement

The current study did not involve a clinical trial per se but did present our correlative data associated with the clinical trial NCT03751436, which was recently published.^70^ Correlative studies involving human samples were conducted in accordance with the Declaration of Helsinki and were approved by the Roswell Park IRB.^70^

### Statistics and reproducibility

Statistical analyses were conducted using GraphPad Prism v10, R, and OriginPro. All results are based on at least two to three independent experiments with biological replicates. For each replication experiment, a new aliquot of cells from the original stock was thawed and cultured. Data normality was assessed using the Shapiro–Wilk test (α = 0.05). If data were normally distributed, parametric tests were used, including unpaired *t*-tests, one-sample *t*-tests (to compare a sample mean against a defined value), and one-way or repeated-measures ANOVA (for multiple group comparisons depending on experimental design). For non-normally distributed data, non-parametric tests such as the Mann–Whitney U test or Kruskal–Wallis test were applied. Multiple group comparisons were performed using one-way ANOVA followed by Tukey’s or Bonferroni’s post hoc test, as appropriate, to adjust for multiple hypothesis testing. Specific statistical tests and sample sizes (*n*) are detailed in the figures and/or figure legends, where *n* refers to the number of animals or samples for in vivo and in vitro experiments, respectively. Data are presented as mean ± SEM or SD, along with individual data points, as specified. Statistically significant differences (*p* < 0.05) are indicated in the graphs; non-significant results are labeled “ns” or left unmarked.

*For correlation analysis*, Pearson’s correlation coefficient (r) was used to assess the linear relationship between AR and BCL-2 expression levels. To evaluate the significance of this correlation, a two-tailed *t-*test was performed using OriginPro software.

*For quantification data plots*, box plots summarizing the relative % of 4 PCa cell subtypes in WM images analyzed in benign tissues, primary tumors and CRPC. Each dot in the box plots represents a CK^+^ ROI (Region of Interest). *P* values were calculated via repeated measure two-way ANOVA with Bonferroni multiple comparison test. *p* < 0.1; *p < 0.05; **p < 0.01; ***p < 0.001; ****p < 0.0001. All data analysis was done using OriginPro software.

*For analysis of* in vitro *and ex-vivo drug treatment data*, at least 3 independent repeats were performed in each experiment. Differences between sample groups were evaluated using Mann–Whitney U-test or Kruskal–Wallis H-test. *P*-value of less than 0.05 was considered statistically significant. Significance levels are indicated as follows: *p < 0.05, **p < 0.01, and ***p < 0.001, respectively. Half-maximal inhibitory concentration (IC_50_) values were calculated using nonlinear regression algorithm in Prism 7 software (GraphPad Software, USA). IC₅₀ values were estimated from relative viable cell counts following treatment with varying drug concentrations. Dose–response curves were generated in GraphPad Prism using a four-parameter logistic model, with response values normalized to the vehicle-treated control (set to 1). IC₅₀ values were calculated from the fitted curves. To compare IC₅₀ values between single-agent and combination treatments across independent experiments, a two-tailed Mann–Whitney U test was performed.

*To determine potential synergy between two treatment conditions*, experiments were performed using incremental dosing of each drug for four weeks. For combination studies, the tumor volume data was annotated as viability data and were loaded into the free software Compusyn and Synergy Finder Plus. Compusyn software implanted Chou-Talalay method to generate dose-effect curves. Combination index below 1 was considered synergistic effect. The SynergyFinder software generated surface response plots by comparing the tumor volume inhibition data to a drug combination reference model obtained from the effect of each drug alone. SynergyFinder implemented a bootstrapping method to compare viability data. We considered a P-value of less than 0.05 to establish statistical differences between drugs combinations.

*For statistical re-analysis of the therapeutic data*^20^ *at the individual tumor levels* (supplementary Fig. 9), tumor volumes were measured weekly using calipers, and volume was calculated using the formula: (length × width²)/2. Individual tumor growth kinetics were plotted over time, and mean tumor volumes ± SEM were computed at each time point. The magenta arrow denotes treatment start point. To assess differences in tumor growth kinetics between groups, log-transformed tumor volumes (log₁₀ mm³) were plotted. A linear mixed model (LMM) was employed to analyze the log10-transformed growth curves, accounting for within-subject correlations. Model parameters were estimated using restricted maximum likelihood (REML). Group differences were assessed by testing the main effect of treatment, as no significant time-by-group interaction was detected following transformation. Statistical significance was determined using two-sided *t*-tests with Satterthwaite’s approximation for degrees of freedom. A p-value less than 0.05 was considered statistically significant.

*For statistical analyses of Omics data*, gene-level comparisons (e.g., *BCL2, AR*) were performed using two-tailed Student’s *t*-test. When raw counts were available, differential expression analysis was performed using DESeq2, with fold change (FC), *p*-values, adjusted *p*-values (FDR), and 95% confidence intervals (CIs) reported (e.g., Fig. 1c, e; supplementary Table 5). For datasets without raw counts, gene expression values (TPM) were analyzed using paired Wilcoxon signed-rank tests for group paired comparison. Pearson’s correlation was used to assess linear associations between gene expression and chromatin features. The Jonckheere–Terpstra (J-T) trend tests were used to assess monotonic relationships between ordinal variables (e.g., Gleason score) and continuous values (e.g., *BCL-2* mRNA levels), using the DescTools R package (v0.99.60). All statistical analyses were conducted using R (v4.3.3) and GraphPad Prism (v10.4.1). Related figures were generated using GraphPad Prism or R as appropriate.

## Supplementary information

Supplementary Figures 1 to 21 and Supplementary Tables 1 to 5

## Data Availability

All experimental data are available upon request from the (co-)corresponding authors. New experimental models (e.g., castration-resistant PCa cell sublines and xenograft models) will be made available upon the publication of the manuscript. All datasets analyzed in this study were obtained from publicly accessible repositories, with accession IDs and download portals provided in supplementary Table 1. The present project does not involve proprietary custom codes.
