# Supplementary material for: Single-cell imaging analysis, therapeutic modeling and a Phase Ib trial validate BCL-2 as a target across heterogeneous castration-resistant prostate cancer
Source: Signal Transduct Target Ther. 2026 May 1;11:161. doi: 10.1038/s41392-026-02700-w (PMC13134962; doi:10.1038/s41392-026-02700-w)
Supplement: Supplementary file 1 — Supplementary Figures 1 to 21 and Supplementary Tables 1 to 5 [file 41392_2026_2700_MOESM1_ESM.docx]

Supplementary Materials for

Single-cell imaging analysis, therapeutic modeling and a Phase Ib trial validate BCL-2 as a target across heterogeneous castration-resistant prostate cancer

Anmbreen Jamroze, Xiaozhuo Liu, Surui Hou, Wen (Jess) Li, Han Yu, Amanda Tracz, Justine Jacobi. Qiuhui Li, Kent Nastiuk, Xin Chen, Jiaoti Huang, Kevin Lin, Mingyu Liu, Changmeng Cai, Yue Lu, Igor Puzanov, Jason S. Kirk, Gurkamal Chatta, Dean G. Tang

Correspondence to: [Anmbreen.Jamroze@Roswellpark.org](mailto:Anmbreen.Jamroze@Roswellpark.org) or [Dean.Tang@Roswellpark.org](mailto:Dean.Tang@Roswellpark.org)

**This file includes:**

Supplementary Figures 1 to 21

Supplementary Tables 1 to 5

**Other Supplementary Materials for this manuscript include the following:**

N/A

1

**Supplementary Figure 1. Analysis of *BCL-2* mRNA expression in normal human prostate**

**(a) and of BCL-2 family members in a scRNA-seq dataset (b-h).**

(**a**) *BCL-2* mRNA is expressed at higher levels in normal human prostate basal (B) and CD38lo luminal progenitor (LP) cells than mature differentiated luminal (L) cells. The original publication (dataset) is indicated on top (ref. 71), and the two bar graphs were derived from RNA-seq (left) and microarray (right), respectively. n indicated in parentheses. **p*<0.05; ***p*<0.01 (paired Student’s *t*-test).

(**b-g**) Our remapping (ref. 76) of Cheng/Huang human prostate/PCa scRNA-seq dataset (ref. 75) and characterization of the cell clusters (or cell types). (**b, c**) Remapping of the 24,142 high-quality cells coupled with *AMACR* expression led to identification of 9 major cell clusters, including 5 *AMACR*-positive PCa cell clusters (i.e., Pri-PCa1, Pri-PCa2, CRPC1 and CRPC NE in the prostate, and mCRPC) and 4 *AMACR*-negative benign cell populations (i.e., intact luminal [Intact Lum] cells, intact basal cells, proximal luminal [Prox Lum] progenitor cells, and castration-resistant epithelial [CR Epi] cells). (**d**) The AR target gene and luminal cell and differentiation marker *KLK3* was expressed at the highest levels in PCa1, PCa2, CRPC1, mCRPC and Intact Lum cells with much lower expression in Intact Basal and Prox Lum cells and no expression in CRPC NE and CR Epi cell populations. (**e, f**) Expression of basal cell markers *KRT5* and *KRT14* in the re-mapped cell populations. Note that both genes were highly expressed in the Intact Basal populations but significant *KRT5* expression was also detected in CR Epi cells. (**g**) *ERG* expression in the remapped cell clusters. Note that *ERG* was highly expressed in the two Pri-PCa and Intact Lum cell populations with very low expression in Intact Basal and Prox Lum clusters but no expression in the rest.

(**h**) mRNA levels of the 4 indicated BCL-2 family members in the remapped cell clusters. The *BCL-xL* and *MCL1* were expressed at high levels, *BCL-W* at low levels and *BFL-1* at barely detectable levels in the 9 cell clusters. Unlike *BCL-2*, these 4 BCL-2 family genes were expressed rather homogeneously and at similar levels across all cell types including the 3 CRPC specimens.

**Supplementary Figure 2. Changes in AR (signaling), GR and BCL-2 in the 4 AD/AI xenograft models used in this study.**

(**a**) Schema for generating the matched AD/AI xenograft models.

(**b**) IHC staining of AR showing predominantly nuclear AR in all 4 androgen-dependent (AD) models (top). In contrast, the 4 corresponding androgen-independent (AI) xenografts displayed model-related alterations in AR, i.e., LNCaP-AI becoming nuclear AR^+/hi^, LAPC9-AI AR^-/lo^, and LAPC4-AI and VCaP-AI largely AR^cyto^ (bottom). Passage (P) numbers of tumors were indicated.

(**c-e**) Alterations of AR, AR targets (PSA and FKBP5), GR, and BCL-2 in the LAPC9 and LAPC4 models. In (**c**), the LAPC9-AI tumors (i.e., 1^o^ CRPC) showed passage-dependent decreases

in AR and AR targets (PSA and FKBP5) with no changes in GR while LAPC4-AI tumors showed increased AR and GR but decreased AR targets. In both models, BCL-2 was upregulated in

AI tumors. In (**d**), castration/Enza resistant LAPC9 2^o^ CRPC continued to show lack of AR and PSA and reduced FKBP5 but increased BCL-2 with no major changes in GR. Note both LAPC9

1^o^ and 2^o^ CRPC lacked ARv7. In (**e**), the LAPC4 2^o^ CRPC continued to express increased AR, GR and BCL-2.

(**f-h**) Alterations of AR, AR targets (PSA and FKBP5), GR, and BCL-2 in VCaP and LNCaP models.

In (**f**), VCaP-AI tumors (i.e., 1^o^ CRPC) showed increased AR, ARv7 and GR but reduced BCL-2 while LNCaP-AI tumors showed increased AR and BCL-2 and lack of ARv7 without consistent changes in GR. *Non-specific band. In (**g**), castration/Enza resistant LNCaP 2^o^ CRPC continued to show lack of ARv7 but increased AR, GR and BCL-2 with slightly reduced PSA and FKBP5 levels. *A GR splice variant or non-specific band. In (**h**), the VCaP 2^o^ CRPC continued to show increased AR but reduced PSA and BCL-2 with slightly increased GR (compared to 1^o^ CRPC).

Note that the anti-AR antibody used in panels (**b-h**) was the mouse mAb AR 441 that was raised against the human N-ter AR (aa 299-315; Supplementary Table 2) and recognizes full-length AR as well as the C-ter truncated AR variants. This Supplementary Figure was put together to demonstrate, in one place, the expression and changes of AR, ARv7, AR activity (PSA and FKBP5), GR, and BCL-2 in our 4 AD/AI xenograft models. Part of panels **c**, **d**, **f** and **g** were

adapted from ref. 20 while panel **e** was part of Fig. 6a (this study). The WB panels (**c-h**) were used towards quantifications in Fig. 1f.

**Supplementary Figure 3.**

**
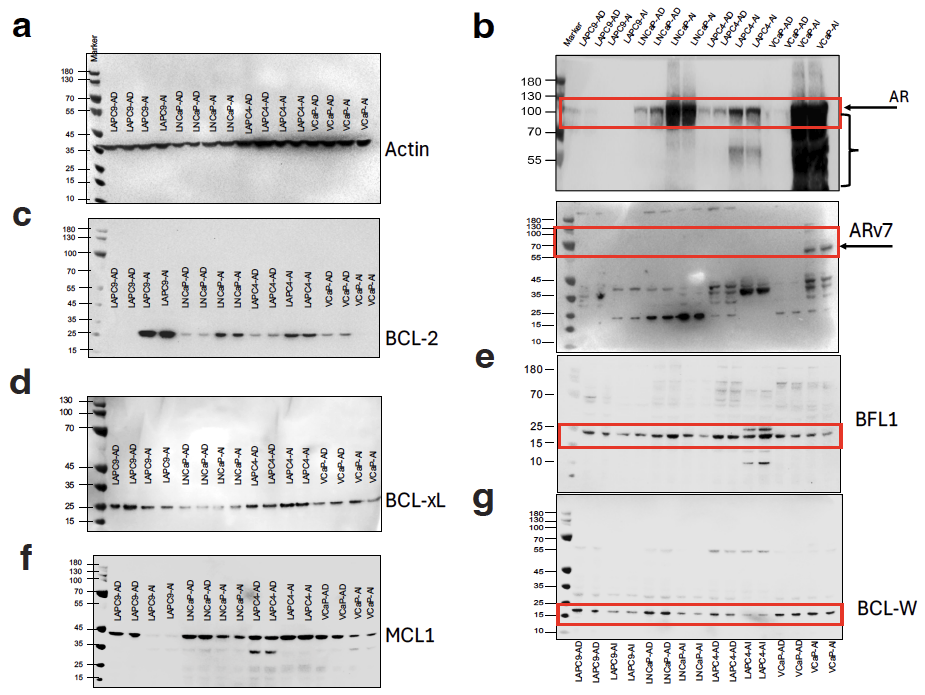
**

**Supplementary Figure 3. Full WB gel images of AR, ARv7, and BCL-2 family members in our 4 xenograft AD/AI models.**

**(a)** Loading control β-actin.

**(b)** Top, AR WB using the rabbit anti-AR mAb D6F11 (raised against the N-ter of human AR; Supplementary Table 2) which detects both full-length AR (110 kDa, arrow) and the C-ter truncated AR splice variants (bracket on the right). Bottom, WB of ARv7 protein using an ARv7-specific antibody. Note that VCaP-AI is the only model that expressed the ~75 kDa ARv7 protein. The anti-ARv7 antibody also detected many lower M.W bands in most samples, which might be degradation products or non-specific.

**(c)** WB showing the 26 kDa BCL-2 upregulated in LAPC9-AI, LNCaP-AI and LAPC4-AI but reduced in VCaP-AI.

**(d-g)** WB showing specific detection of BCL-xL (26 kDa; **d**), BFL1 (~20 kDa; **e**), MCL-1 (~40 kDa; **f**), and BCL-W (22 kDa; **g**) in the 4 AD/AI models.

Whole cell lysates from two individual AD and AI tumors for each model were used in WB analysis using antibodies presented in Supplementary Table 2. These gels were used, together with others, in quantifying the relative protein levels shown in the bar graphs in Fig. 1f.


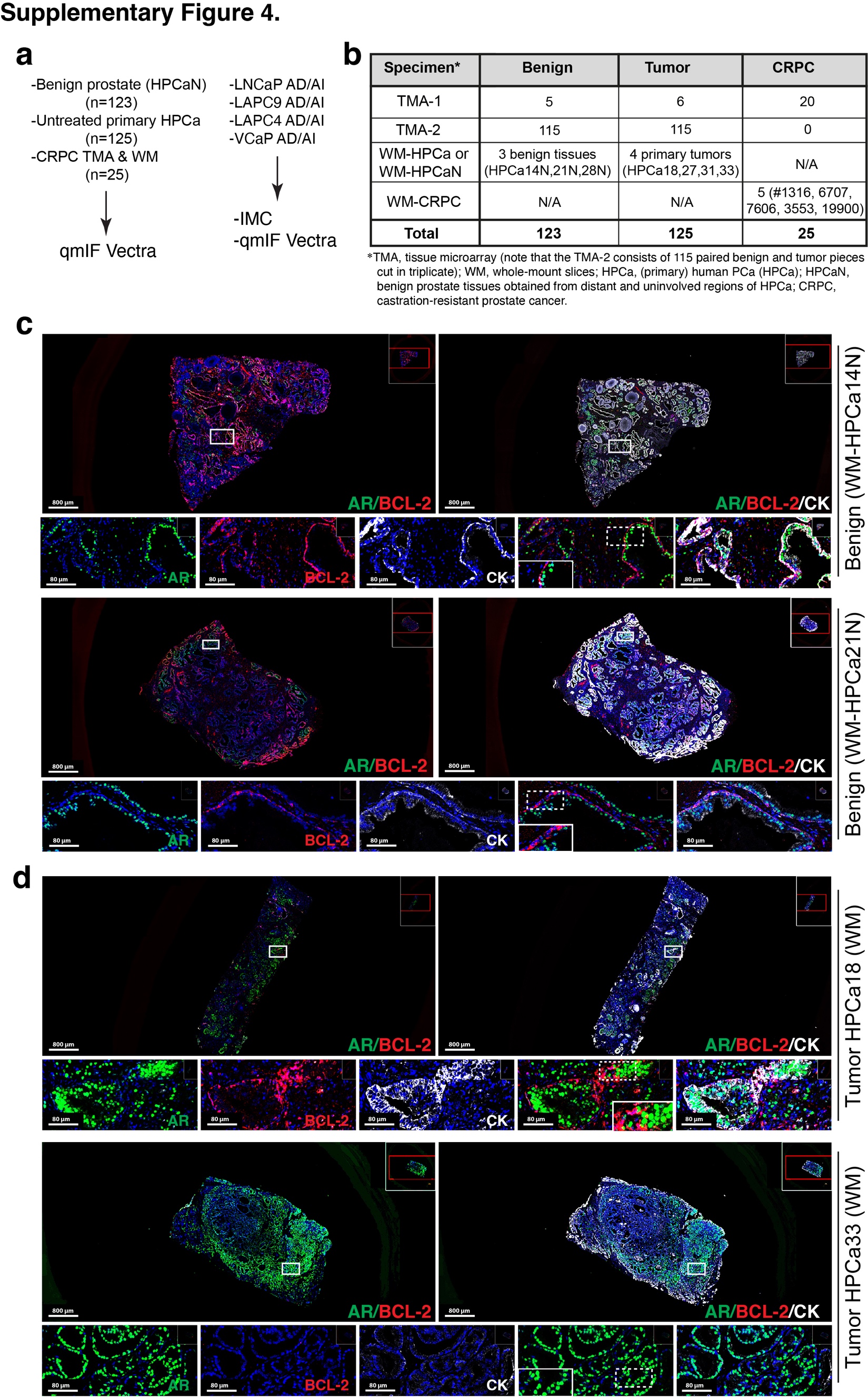


**Supplementary Figure 4. Experimental scheme of Vectra-based qmIF analysis and IMC and increased AR^+^BCL-2^-^ cells in treatment-naïve primary PCa.**

**(a)** Patient samples and experimental models used in qmIF and IMC analyses.

**(b)** Tabulated summary of Specimen types and numbers used in the current study.

**(c)** Representative qmIF images from whole-mount (WM) benign prostate (HPCa14N and HPCa21N) sections stained for AR, BCL-2, and cytokeratin (CK) using the Vectra Polaris platform. Top panels show low-magnification overviews (1.5x) for full tissue context. Bottom panels show 40x magnification, including individual AR and BCL-2 images and composite views illustrating their spatial relationship. Dotted squares in 40x overview indicate regions further magnified at 80x. Note the reciprocal expression patterns of AR (localized to luminal epithelial cells) and BCL-2 (localized to basal cells) proteins.

**(d)** Representative qmIF images from WM primary PCa (HPCa18 and HPCa33) sections stained for AR, BCL-2, and CK using the Vectra Polaris platform. Top panels show low-magnification (1.5x) overviews of the entire tissue sections. Bottom panels display higher magnification views, including individual and composite images of AR and BCL-2. Note that primary tumors exhibit a marked increase in AR⁺BCL-2⁻ cells when compared to benign prostate tissue.


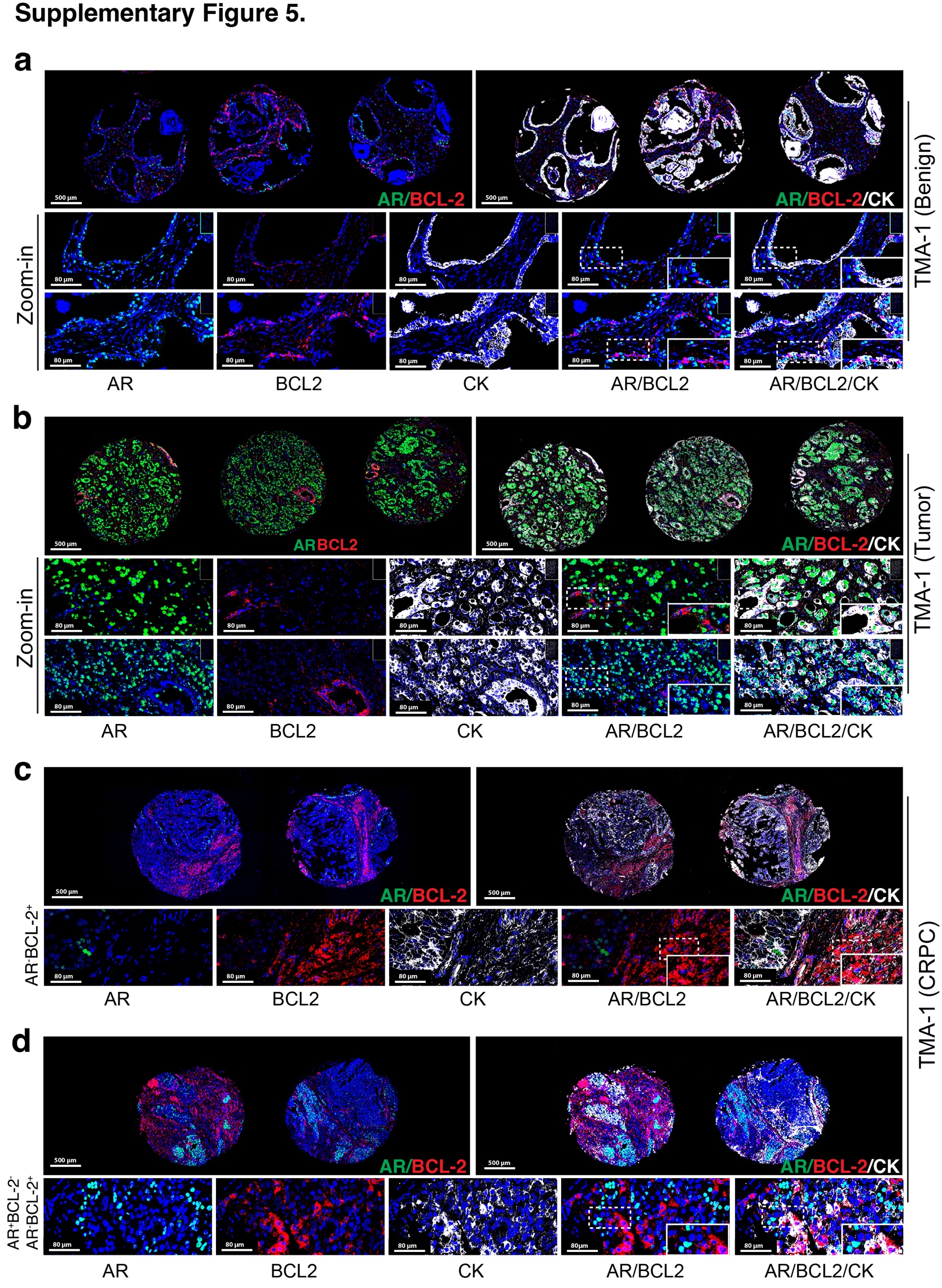


**Supplementary Figure 5. qmIF analysis of dynamic changes in AR^+/-^ and/or BCL-2^+/-^cells in TMA-1.**

**(a)** Benign prostatic glands have AR^+^ and BCL-2^+^ cells in the luminal and basal layers, respectively. Shown on top are low-magnification (5.9x) WM images of 3 benign tissues and below are zoom-in (40x) images of 2 representative areas stained for individual markers. Dotted boxes indicate areas further magnified in the in-sets (80x) demonstrating AR⁺ luminal cell and BCL-2⁺ basal cell localization.

**(b)** Primary tumors are characterized by a dramatically expanded AR^+^BCL-2^-^ PCa cell population. Shown on top are WM images of 3 tumors and at the bottom are zoom-in (40X) images of 2 representative areas stained for individual markers. Dotted boxes denote areas further magnified (80x). Note most PCa cells are AR^+^BCL-2^-^.

**(c-d)** CRPC are characterized by significantly increased (AR^+/-^)BCL-2^+^ PCa cells. Shown on top are WM images of 2 CRPC (5.9x) each and at the bottom are zoom-in (40x) images of representative areas stained for individual markers. Dotted boxes indicate areas shown in 80x in-sets. Note markedly increased BCL-2^+^ PCa cells.


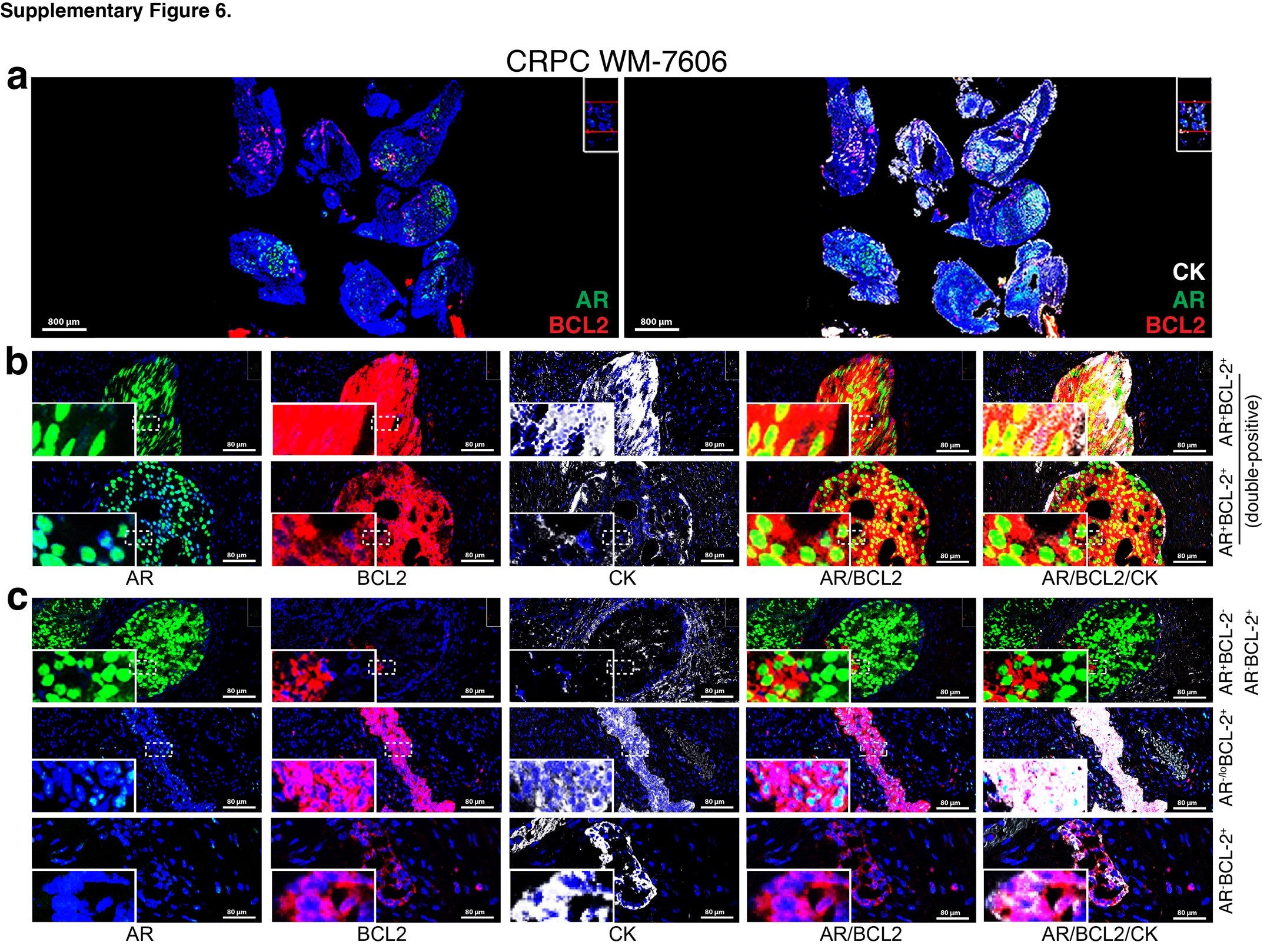


**Supplementary Figure 6. Increased diversity in AR^+/-^BCL-2^+/-^ cells and increased BCL-2^+^ PCa cells in CRPC.**

(**a**) Representative qmIF images from a WM CRPC specimen (CRPC-7606). Shown are low-magnification (0.4x) composite images of the entire tissue section, displaying AR/BCL-2 (left) and AR/BCL-2/CK (right).

(**b**) Two distinct regions of interest (ROIs) imaged at 40x, highlighting the double-positive AR+BCL-2+ CRPC cells. Dotted boxes indicate areas selected for further high-resolution imaging at 160x, shown in the insets.

(**c**) Three spatially distinct ROIs from CRPC-7606 imaged at 40x, each highlighting heterogeneous AR and BCL-2 expression. Insets show further magnified views at 160x, demonstrating distinct cell subtypes including AR⁺BCL-2⁻, AR⁻BCL-2⁺, AR⁺BCL-2⁺ (double-positive), and ARloBCL-2⁺ cells.


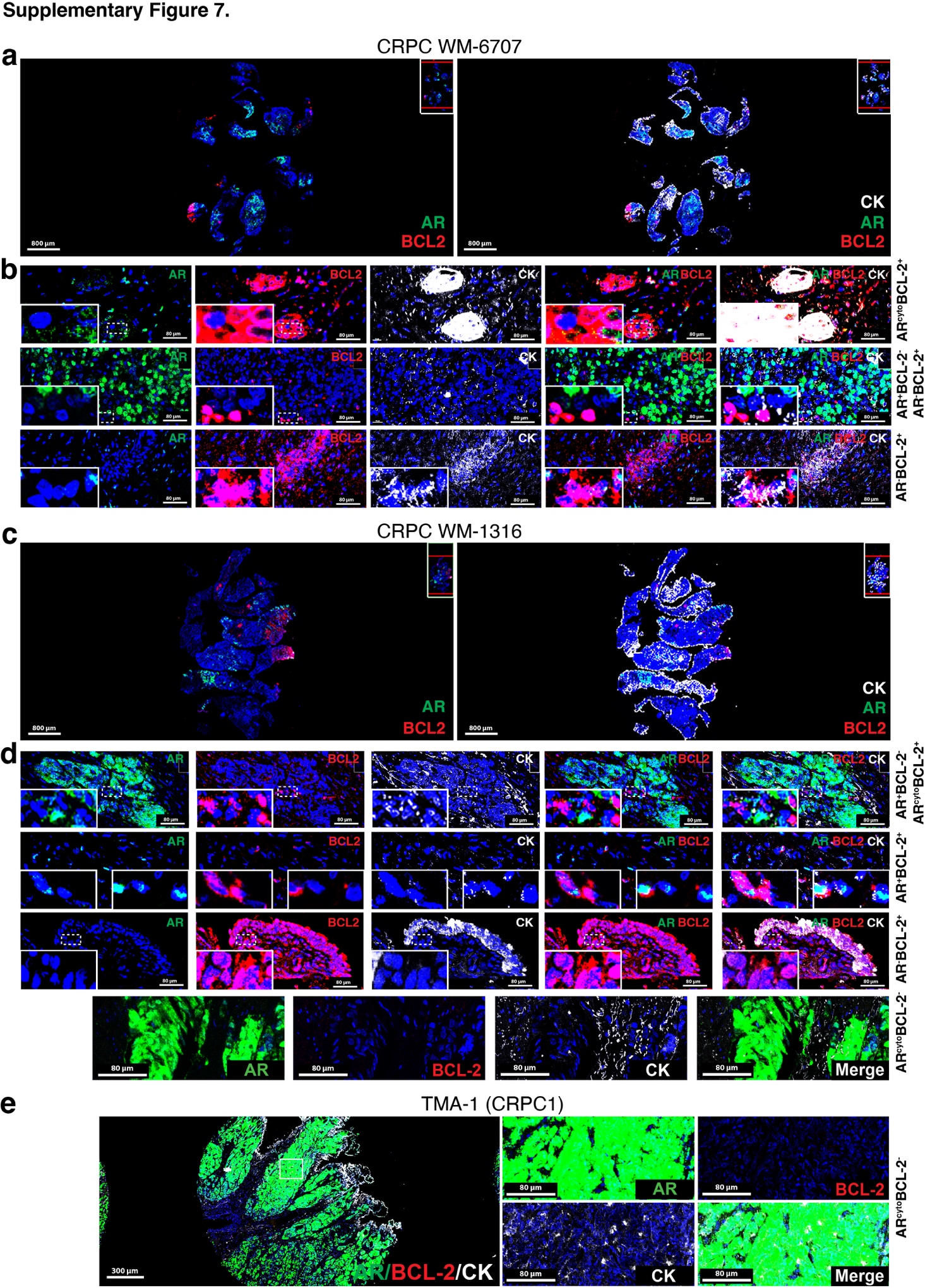


**Supplementary Figure 7. Increased diversity in AR^+/-^BCL-2^+/-^ cell subpopulations and increased BCL-2^+^ PCa cells in CRPC.** Analysis of AR+/-BCL-2+/- PCa cell subtypes in CRPC WM-6707 (**a-b**) and CRPC WM-1316 (**c-d**). For each CRPC WM, shown on top are AR/BCL-2 (left) and AR/BCL-2/CK (right) WM images and below are high-magnification images of 3 ROIs illustrating the indicated subtypes of CRPC cells (right). **(e)** Further demonstration of ARcytoBCL-2- CRPC cells in the CRPC1 of TMA-1.


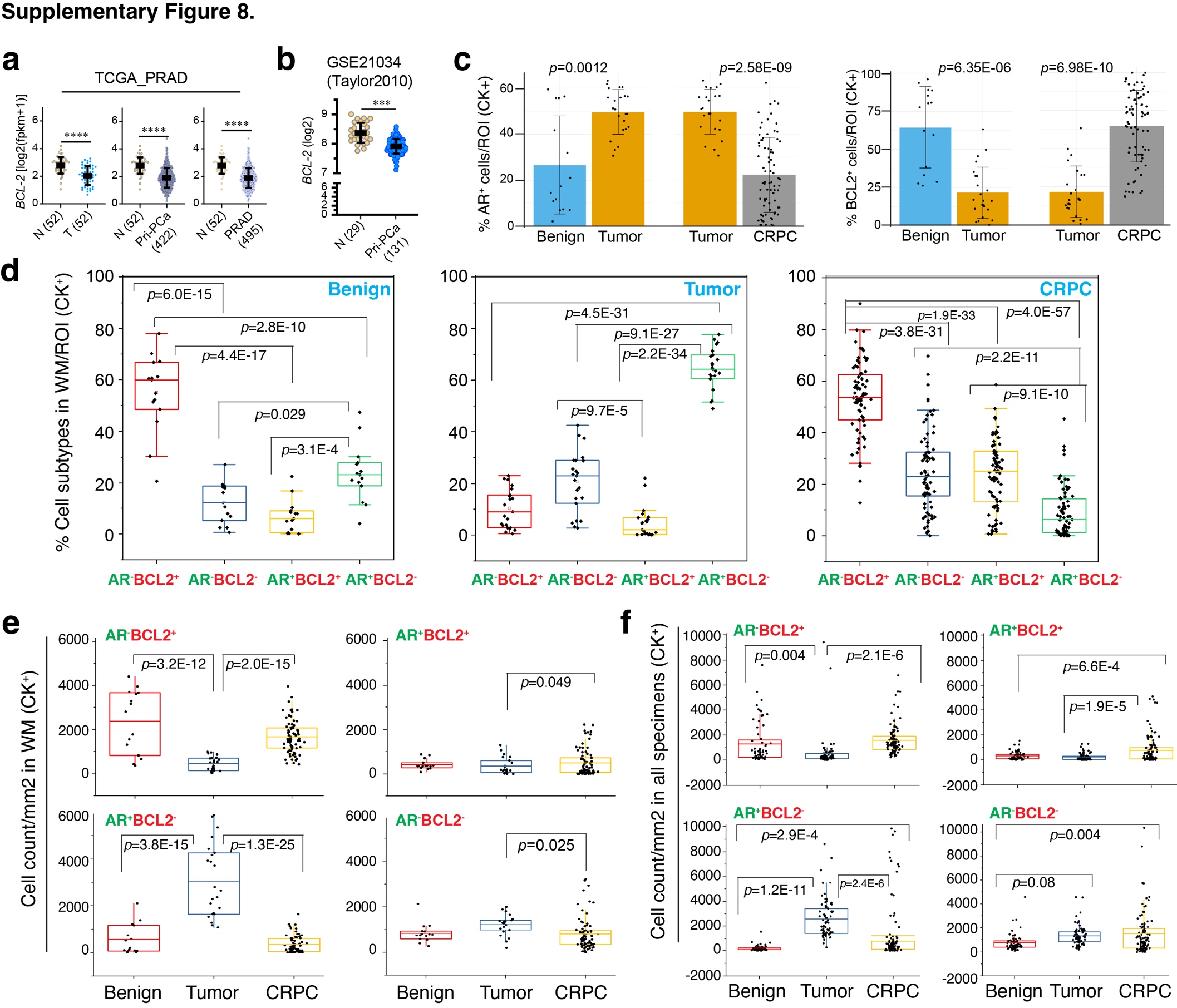


**Supplementary Figure 8. Quantitative summary of AR^+/-^BCL-2^+/-^ cell subtypes in primary PCa and CRPC.**

1. *BCL-2* mRNA levels were reduced in primary PCa (Pri-PCa) in the TCGA_PRAD database. Patient numbers (n) are indicated in parentheses. Three comparisons are shown: matched pairs of prostate tumor (T) and normal (N; tumor-adjacent benign) tissues (n = 52 pairs; *****p*<0.0001, Paired Student’s *t*-test); N vs. Pri-PCa (treatment-naïve tumors in TCGA-PRAD, excluding samples from patients who received adjuvant [post-surgery] hormone therapy, n = 52 vs. 422; *****p*<0.0001, Student’s *t*-test); and N vs. all PRAD tumors (n = 52 vs. 495; *****p*<0.0001, Student’s *t*-test). Expression is shown as log₂ (FPKM + 1).
2. *BCL-2* mRNA levels were reduced in Pri-PCa in the Taylor dataset. Patient numbers (n) are indicated in parentheses. *** *p*<0.001 (Student’s *t*-test).
3. Quantification of the percentage of AR⁺ (left) and BCL-2⁺ (right) cells among epithelial cells (CK⁺) in benign prostate tissue, untreated primary tumors, and CRPC specimens. Each dot represents an individual region of interest (ROI). Bars represent mean ± SD. *P* values were determined using the Wilcoxon rank-sum test and are shown above each comparison.
4. Proportion of PCa cell subtypes (AR⁺BCL-2⁻, AR⁻BCL-2⁺, AR⁺BCL-2⁺, AR⁻BCL-2⁻) within CK⁺ regions across benign, primary tumor, and WM CRPC tissues. Group means were compared using one-way ANOVA with Bonferroni correction.
5. Mean cell density (cells/mm²) of each AR/BCL-2-defined cell subtype within CK⁺ compartments from whole-mount tissues. Statistical comparisons were performed using one-way ANOVA with Bonferroni adjustment. Each point represents a single ROI.
6. Cell density analysis of AR^+/-^BCL-2^+/-^ PCa cell subtypes pooled across all CK⁺ specimens, including both whole-mount and TMA samples. Data are shown as box plots with means compared by one-way ANOVA and Bonferroni post hoc testing. *p* values reflect significant pairwise differences.

For **d-f**, each dot in the box plots represents one CK^+^ ROI, and *p* values for individual comparisons are indicated (repeated measures two-way ANOVA with Bonferroni multiple comparison test)

**Supplementary Figure 9. AR heterogeneity in primary CRPC linked to distinct Enza response**.

Distinct Enza responses in the four primary (1^o^) CRPC (AI) models, i.e., the nuclear AR^+/hi^ LNCaP-AI (**a**), AR^-/lo^ LAPC9-AI (**b**), AR^cyto^ LAPC4-AI (**c**) and AR^cyto^ VCaP-AI (**d**). Shown in left panels are individual tumor volume measurement (red, control (CTL) mice; blue, Enza-treated mice. Animal numbers for each group are indicated in parentheses on the right). The downward arrows indicate the starting time of Enza treatment (i.e., at 1, 2.5, 4 and 5 weeks, respectively, for LNCaP, LAPC9, LAPC4 and VCaP 1o CRPC). Shown in middle panels are the mean tumor volumes of the CTL (red) and Enza treatment (blue) groups. **p*<0.05 at the time points compared between the two groups (paired Student’s *t*-test). Shown in right panels are differences in tumor growth kinetics between groups using log-transformed tumor volumes. Group differences were assessed by testing the main effect of treatment, and statistical significance was determined using two-sided t-tests with Satterthwaite’s approximation for degrees of freedom (Methods).

**
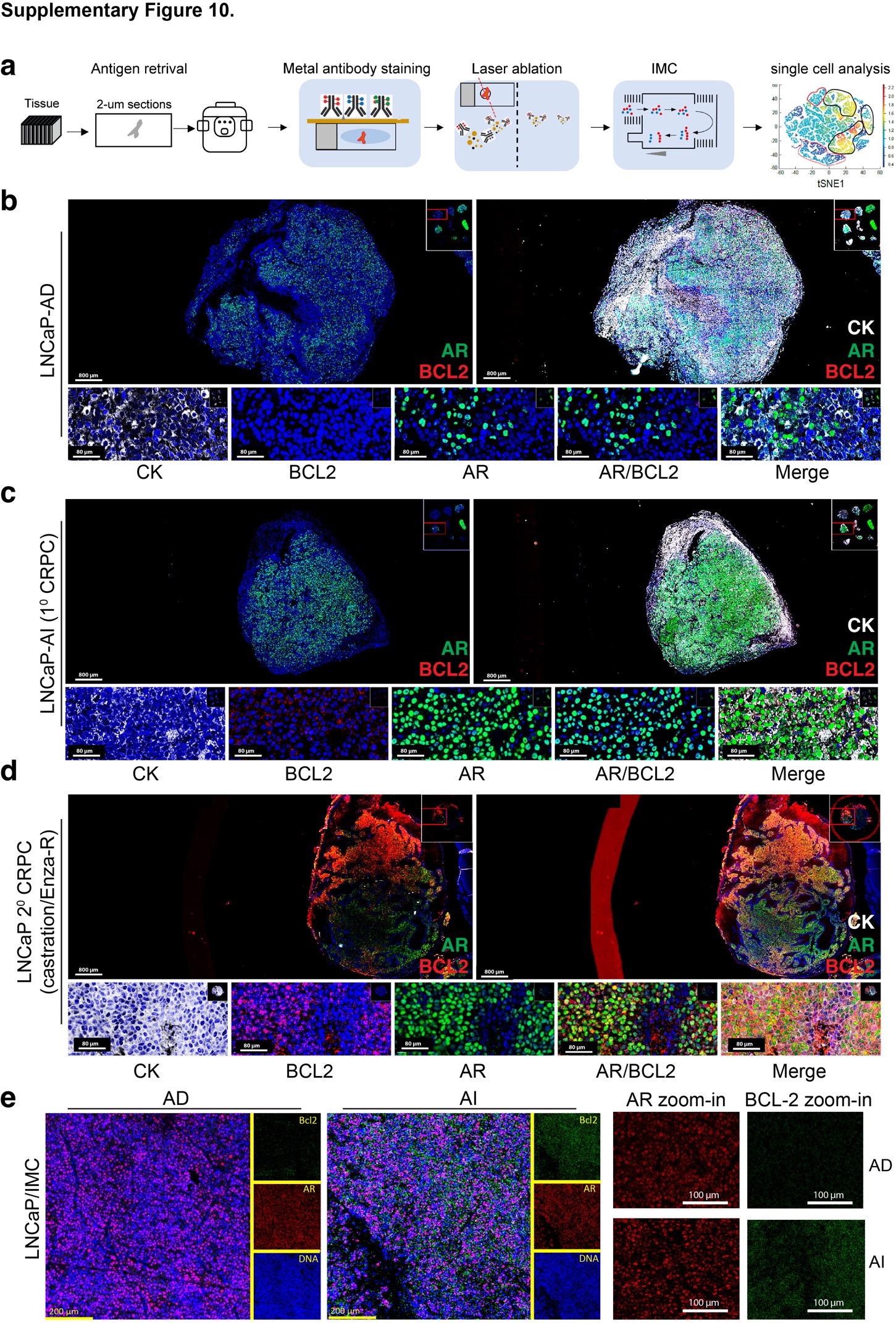
**

**Supplementary Figure 10. Dynamic changes in AR^+/-^BCL-2^+/-^ PCa cell types across the LNCaP-AD, and 1^º^ and 2 ^º^ LNCaP-CRPC models.**

1. Schematic workflow for IMC analysis.
2. qmIF images of LNCaP-AD tumors stained for AR, BCL-2, and CK, with top panels showing full-tissue views (1.5x) and bottom panels showing enlarged (40x) images highlighting AR⁺BCL2^-^ cells.
3. Primary LNCaP-AI (1^º^ CRPC) tumors showed markedly increased AR^+/hi^ cells and slightly increased BCL-2^+^ PCa cells. (top: 1.5x, bottom: 40x).
4. Secondary castration- and Enza-resistant LNCaP-AI (2^º^ CRPC) tumors displayed an AR⁺BCL-2⁺ phenotype in most tumor cells. (top: 1.5x, bottom: 40x).
5. Representative IMC images of LNCaP-AD and LNCaP-AI (1^º^ CRPC) tumors stained for AR, BCL-2, and DNA. Shown on the right are representative zoom-in images of AR and BCL-2 in the AD/AI tumors.

For **b-d**: the top panels show merged, zoomed-out views along with corresponding individual marker channels (AR, BCL-2, and DNA shown in distinct colors). Bottom panels display higher-resolution images highlighting spatial reorganization of marker expression patterns.

**Supplementary Figure 11. Dynamic changes in AR^+/-^BCL-2^+/-^ PCa cell subtypes in LAPC9-AD/AI xenograft models.**

1. qmIF images of LAPC9-AD tumors stained for AR, BCL-2, and CK. Top panels show full-tissue views at 1.5x; bottom panels show 40x magnified ROIs highlighting AR⁺BCL-2^-^ cells.
2. LAP C9-AI (1^º^ CRPC) tumors are populated by AR^-^BCL-2^+^ cells. Top panels show 1.5x full-tissue views; bottom panels present 40x magnified ROIs highlighting the AR^-^BCL-2^+^ cells.
3. Representative IMC images of LAPC9-AD and LAPC9-AI tumors stained for AR, BCL-2, and DNA. Shown below are images acquired at 100 µm resolution (bottom) to validate AR/BCL-2 expression dynamics under androgen deprivation.


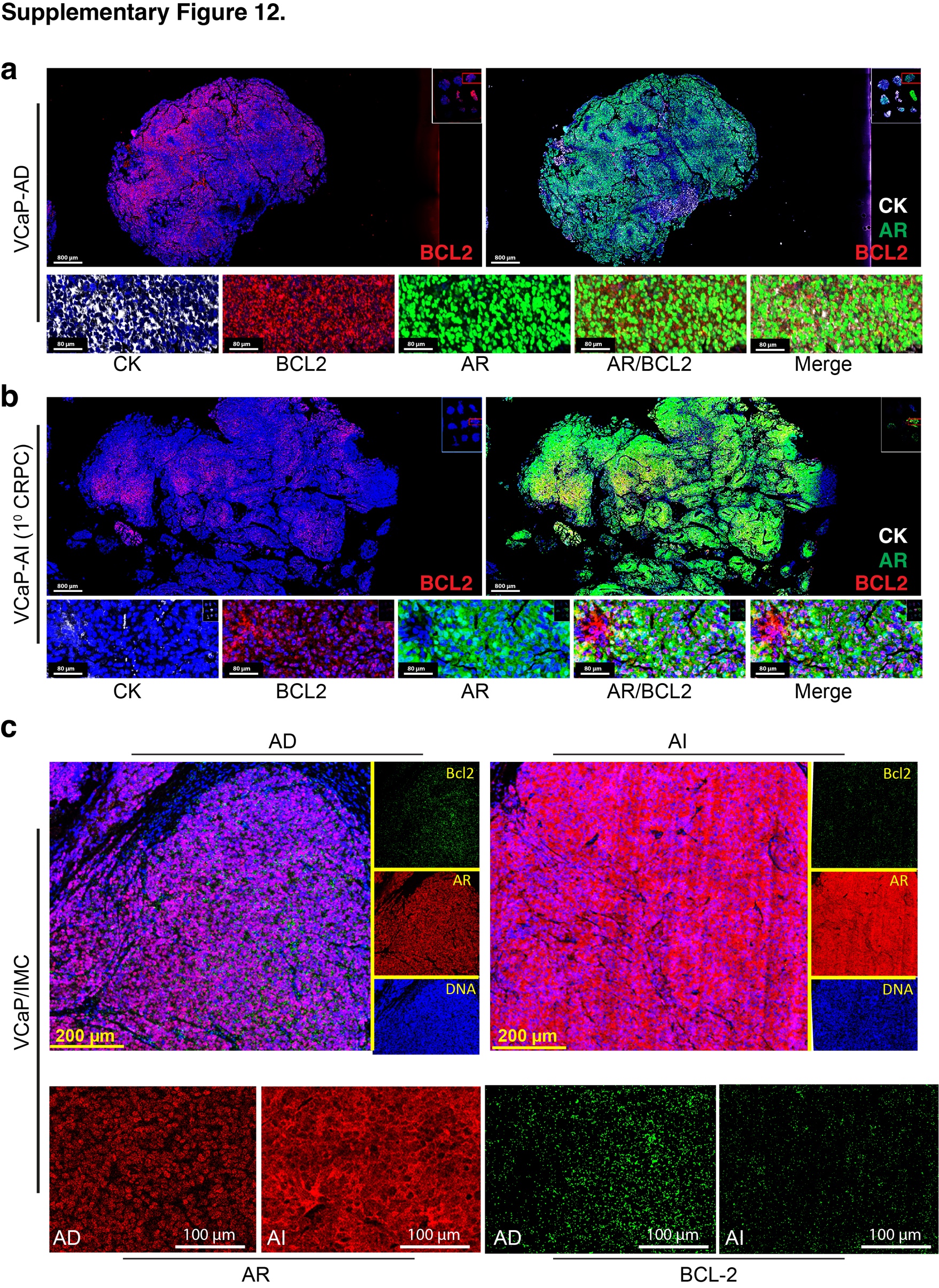


**Supplementary Figure 12. Dynamic changes in AR^+/-^BCL-2^+/-^ PCa cell types in VCaP-AD/AI xenograft models.**

1. qmIF images of VCaP-AD tumors stained for AR, BCL-2, and CK. Shown on top are WM images (1.5x) of BCL-2 staining alone (left) and compound AR, BCL-2 and CK staining (right). Shown below are 40× magnified ROIs highlighting the AR⁺BCL-2^+^ VCaP-AD cells.
2. V CaP-AI (1º CRPC) tumors are populated mostly by AR^cyto^BCL-2^-/lo^ PCa cells. Shown on top are WM images of BCL-2 staining alone (left) and compound AR, BCL-2 and CK staining (right). Shown below are 40× magnified ROIs highlighting the AR^cyto^BCL-2^-/lo^ phenotype of VCaP-AI cells.
3. Representative IMC images of VCaP-AD and VCaP-AI tumors stained for AR, BCL-2, and DNA (top). Shown below are images acquired at 100 µm resolution, illustrating changes in AR and BCL-2 protein expression and localization in response to androgen deprivation.


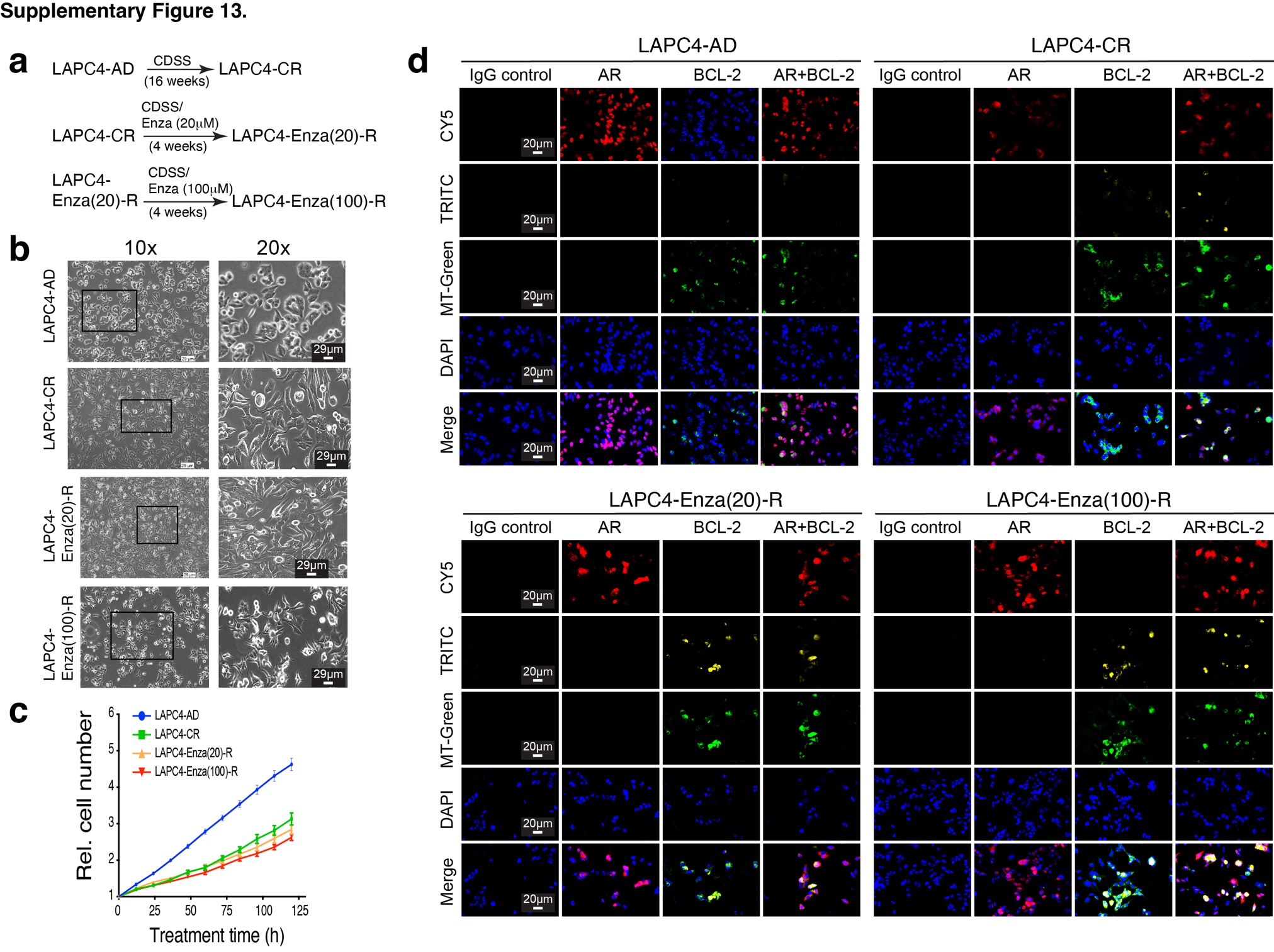


**Supplementary Figure 13. Generation and phenotypic characterization of castration-resistant LAPC4 sublines.**

1. Schematic of the treatment protocol to generate resistant sublines. LAPC4-AD cells were first cultured in CDSS to create LAPC4-CR cells. Chronic treatment of LAPC4-CR cells with 20 μM or 100 μM enzalutamide (Enza) for 4 weeks produced LAPC4-Enza(20)-R and LAPC4-Enza(100)-R sublines, respectively.
2. Phase-contrast images (10x and 20x magnifications; original scale bar for 20x images = 29 μm) showing the morphology of LAPC4-AD, LAPC4-CR, LAPC4-Enza(20)-R, and LAPC4-Enza(100)-R cells. All

castration-resistant LAPC4 cell sublines exhibited altered morphology compared to parental (LAPC4-AD) cells.

1. Cell proliferation assays showing relative cell numbers of each subline over 5 days. All castration-resistant LAPC4 sublines showed reduced growth kinetics compared to the parental LAPC4-AD cells.
2. Multiplex immunofluorescence images (40x) of LAPC4-AD, LAPC4-CR, and Enza-resistant sublines [LAPC4-Enza (20)-R and LAPC4-Enza(100)-R] stained for AR (Cy5, red), BCL-2 (TRITC, yellow), mitochondria (MitoTracker, green), and nuclei (DAPI, blue). Isotype (IgG) controls are shown for each condition. Merged panels display combined marker expression across cell lines. Scale bar, 20 μm for all panels (illustrated on the left-most panels).

**
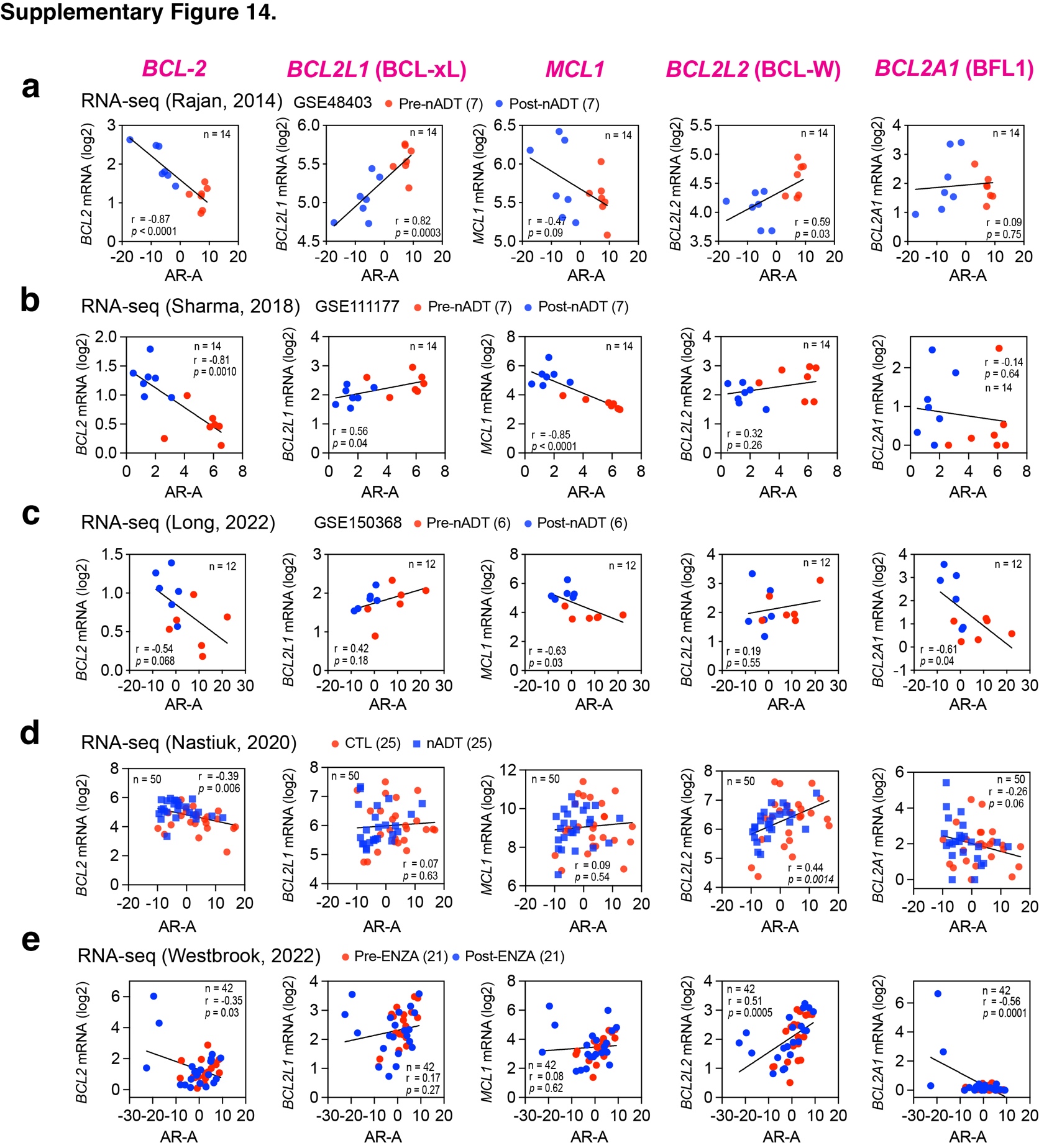
**

**Supplementary Figure 14. Consistent anti-correlation between AR activity and mRNA levels of *BCL-2* (but not other BCL-2 family members) across independent PCa cohorts treated with nADT or Enza.**

(**a–e**) Scatterplots showing correlations between AR activity (AR-A score, x-axis) and mRNA expression (log₂, y-axis) of BCL-2 family members (*BCL-2, BCL2L1* (encoding BCL-xL), *MCL1*, *BCL2L2* (encoding BCL-W), and *BCL2A1* (encoding A1/BFL-1)) in 4 nADT RNA-seq datasets, i.e., (**a**) Rajan 2014 (GSE48403; n = 14), (**b**) Sharma 2018 (GSE111177; n = 50), (**c**) Nastiuk 2020 (n = 42), and (**d**) Long 2022 (GSE150368; pre- and post-nADT paired samples, n = 12), and in (**e**) Westbrook 2022 (pre- and post-Enza paired samples, n = 21). Each panel displays Pearson’s correlation coefficient (r) and *p* value. Note that consistently, the *BCL-2* mRNA levels negatively correlated with AR-A across all 5 datasets whereas other BCL-2 family members showed variable, weak and/or inconsistent associations between the two.

**
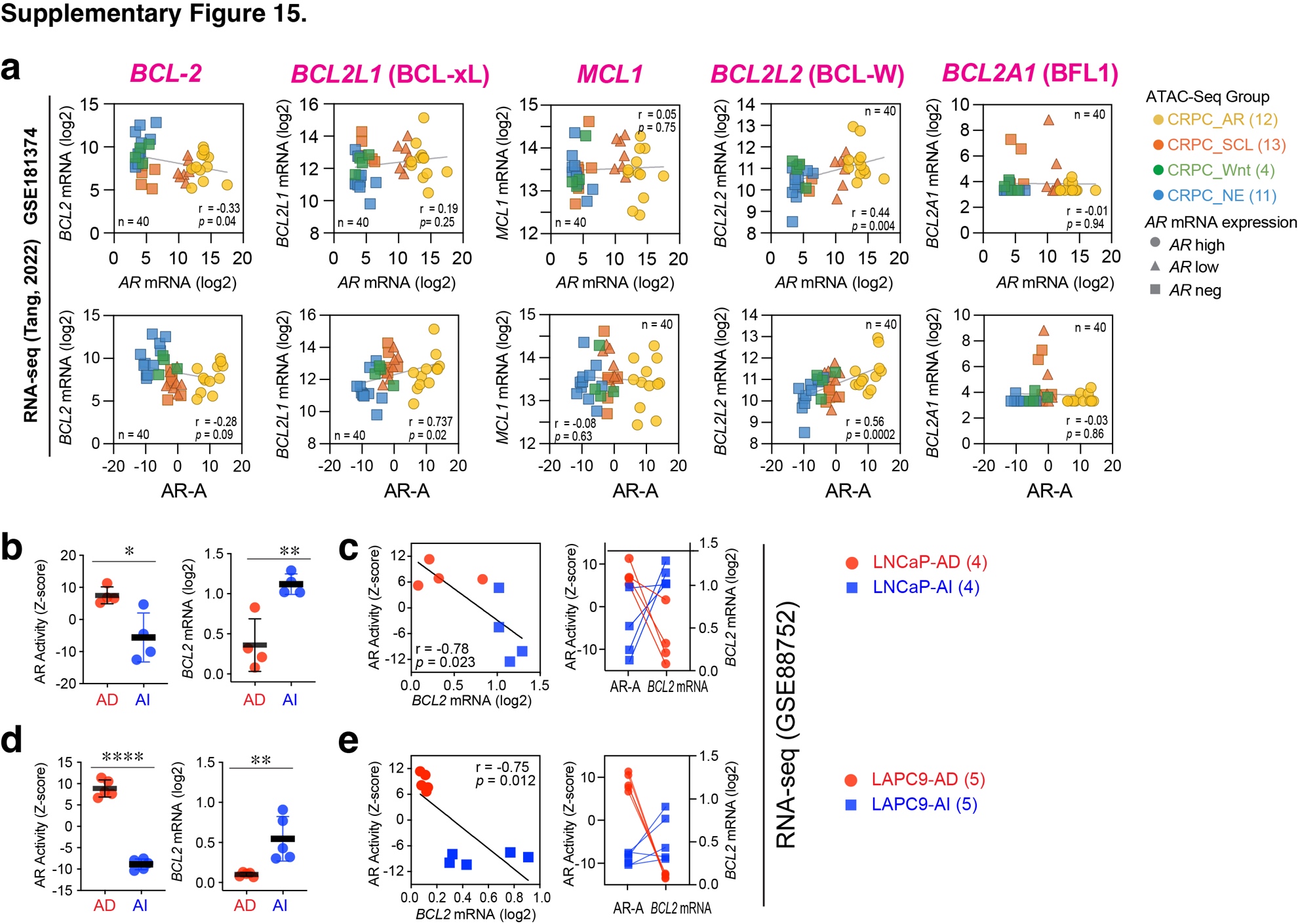
**

**Supplementary Figure 15. Inverse correlation between AR activity and *BCL-2* mRNA levels in the Tang et al dataset (a) and in our LNCaP and LAPC9 xenograft models (b-e).** (**a**) Scatterplots showing correlations between *AR* mRNA levels (top panels, x-axis) or AR-A (bottom panels, x-axis) and the mRNA levels (log₂, y-axis) of the indicated BCL-2 members in GSE181374 with 40 CRPC and organoids. Each panel displays Pearson’s correlation coefficient (r) and *p* value. (**b-e**) Inverse correlation between AR activity (AR-A) and *BCL-2* mRNA levels in LNCaP-AD/AI (**b, c**) and LAPC9-AD/AI (**d, e**) models. Both AI models exhibited decreased AR-A with elevated BCL-2 mRNA expression (**a, c**) and an inverse correlation between AR-A and BCL-2 levels (**b, d**). **p*<0.05, ***p*<0.01, *****p*<0.0001 (Student’s *t*-test).

**
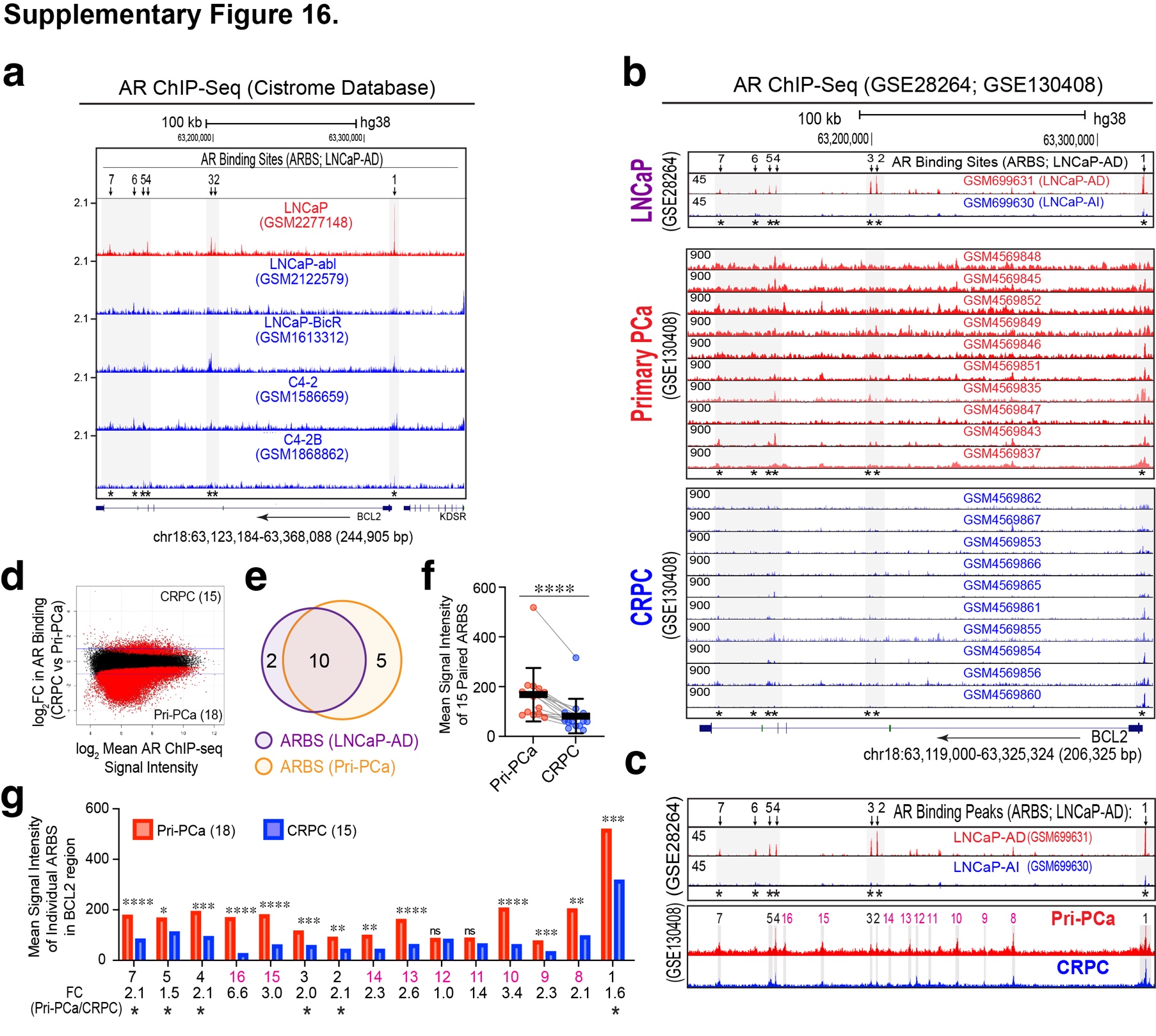
**

**Supplementary Figure 16. Androgen-dependent AR occupancy at the *BCL-2* genomic region Is lost in CRPC.**

(**a**) AR ChIP-seq profiles from Cistrome datasets demonstrate AR binding at the *BCL-2* locus in the indicated LNCaP cell sublines (datasets indicated in the parentheses below).

(**b–g**) Loss of AR Binding at the *BCL-2* locus in patient CRPC as compared to primary PCa (Pri-PCa; AR ChIP-seq data source: GSE130408). Shown in (**b**) is the genome browser view of AR ChIP-seq at

the *BCL-2* locus in Pri-PCa (10 representative samples out of the total of 18 shown) and mCRPC (total n=15 with 10 representative samples shown). The LNCaP-AD/AI data (adapted from Fig. 5h) was shown on top for alignment. Note that strong AR binding peaks detected in LNCaP-AD (ARBS1–7, shaded in grey) are evident in Pri-PCa but largely absent in mCRPC, mirroring the pattern in LNCaP-AI.

(**c**) Visualization of ARBS peaks at the *BCL-2* locus revealed 15 ARBSs in Pri-PCa, of which 13 exhibited significant AR binding loss in CRPC, while 2 showed no significant difference (**p*< 0.05, ***p*< 0.01, Student’s *t*-test).

(**d**) MA plot illustrating the global loss of AR peaks in CRPC relative to Pri-PCa, including those at the *BCL-2* locus, supporting a model of AR-mediated *BCL-2* repression.

(**e**) Venn diagram showing overlap of 10 ARBSs between Pri-PCa (GSE130408) and LNCaP-AD (GSE28264).

(**f**) Averaged AR ChIP-seq signals across the 15 ARBS showing significant reduction of AR binding in CRPC (paired Wilcoxon test, *****p*<0.0001).

(**g**) Quantification showing 13 of the 15 ARBS peaks at the *BCL-*2 region in Pri-PCa were lost in CRPC (**p*< 0.05, ***p*< 0.01, ****p*< 0.001, *****p*< 0.0001, Student’s *t*-test).

**
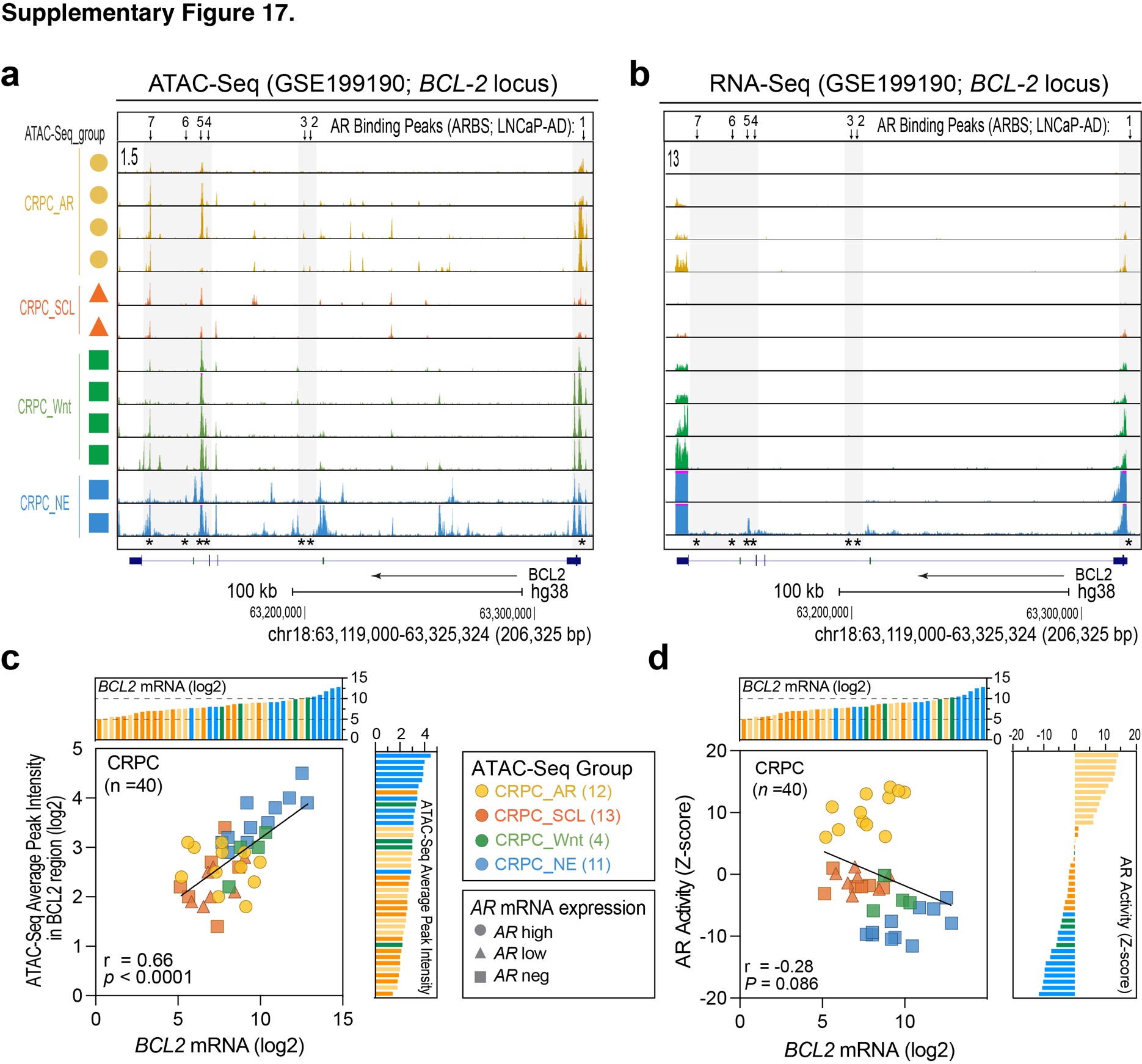
**

**Supplementary Figure 17. The AR^-/lo^ CRPC have increased chromatin accessibility surrounding the *BCL-2***

**genomic region. (a)** Genome browser view of ATAC-seq tracks (GSE199190) at the *BCL-2* locus across CRPC subtypes including CRPC_AR (n=4), CRPC_SCL (n=2), CRPC_Wnt (n=4), and CRPC_NE (n=2). Note that the AR^-/lo^ CRPC (CRPC_NE and CRPC_Wnt) show higher chromatin accessibility at the *BCL-2* genomic region compared to CRPC_AR and CRPC_SCL tumors. **(b)** RNA-seq tracks (GSE199190) from the same representative tumors as in (**a**) illustrate corresponding *BCL-2* mRNA levels, with higher expression in CRPC_NE and CRPC_Wnt. **(c)** Bar-coded correlation plot showing a positive relationship between *BCL-2* mRNA expression and average ATAC-seq peak intensity at the *BCL-2* locus (Pearson r = 0.66, *p*<0.0001). Color-coded distributions highlight that CRPC_NE tumors exhibit both highest chromatin accessibility and *BCL-2* expression, while CRPC_AR tumors show the lowest in both dimensions. Color and shape keys indicating CRPC subtype and AR expression status are shown adjacent to the correlation plots. **(d)** Bar-coded correlation plot demonstrating an inverse relationship between AR activity scores and *BCL-2* mRNA levels across CRPC tumors (Pearson r = –0.28, *p* = 0.086). CRPC_AR tumors exhibit the highest AR activity and lowest *BCL-2* expression, whereas CRPC_NE tumors display the lowest AR activity and highest *BCL-2* expression.


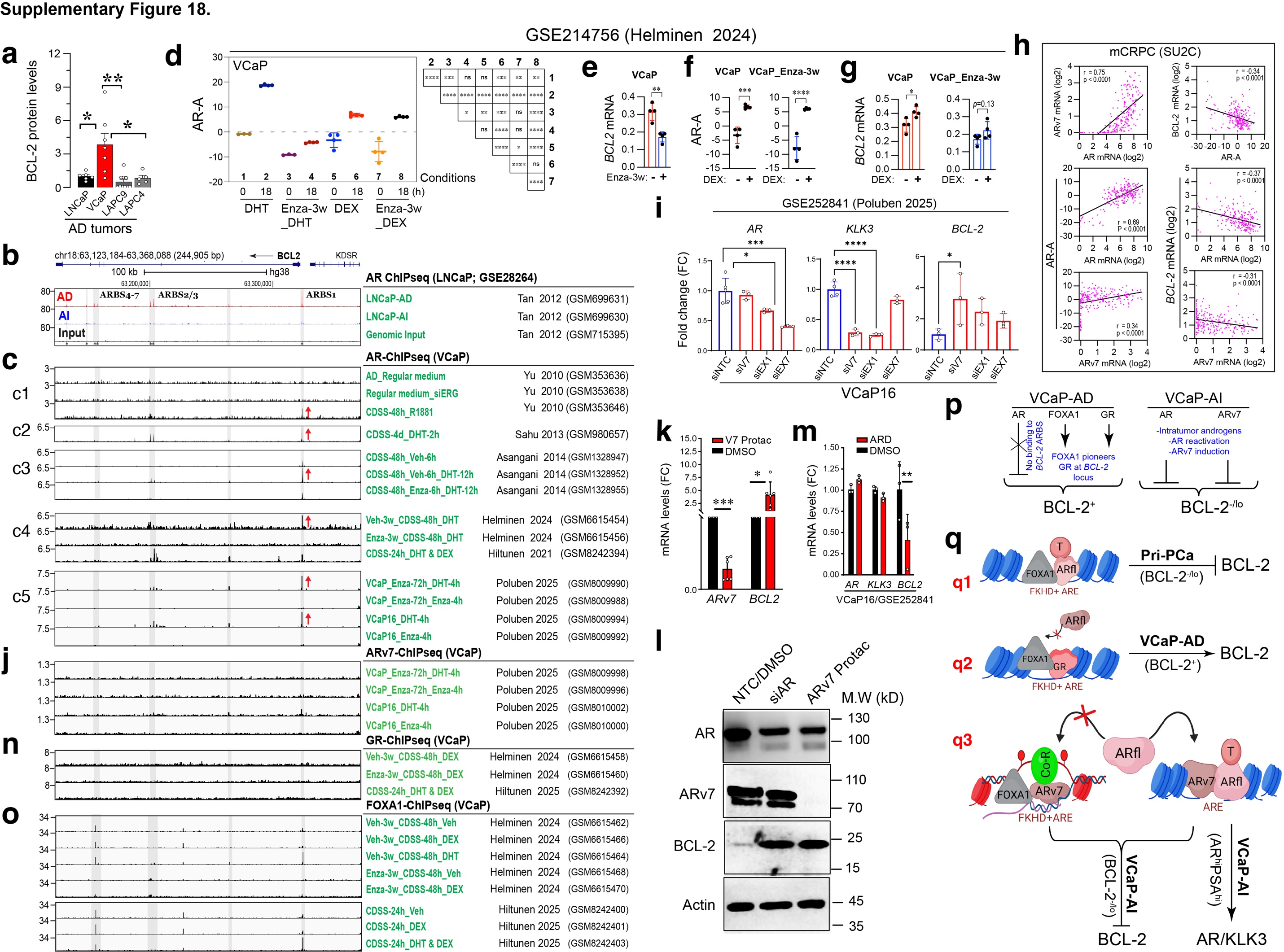


**Supplementary Figure 18. Involvement of AR, ARv7 and GR signaling in generating the BCL-2^-/lo^ phenotype in VCaP-AI.**

1. VCaP-AD tumors have the highest baseline BCL-2 protein levels. Shown is the quantitative presentation of the normalized (relative) BCL-2 protein levels in the 4 AD xenograft modes. Each circle (data point) represented one independent WB BCL-2 protein band, which was normalized to GAPDH. Then the relative BCL-2 levels were further normalized to those in the LNCaP-AD model (which was set at 1).
2. AR ChIP-seq IGV plots showing robust AR binding at the ARBS1 and several other ARBSs in LNCaP-AD cells, which are all lost in LNCaP-AI cells. Genomic input is shown for reference.
3. AR binding profiles in the ARBS of *BCL-2* genomic region in VCaP cells under various androgen conditions. AR ChIP-seq tracks in (c1-c4) VCaP cells cultured in regular medium (VCaP-AD), CDSS (VCaP_CDSS) for various intervals or Enza for 3 weeks (VCaP_Enza-3w) and in (c5) VCaP16 (Enza-resistant VCaP derivative selected in 16-μM enzalutamide for 8 weeks) cells showing increased AR binding to ARBS1 of the *BCL-2* region in these cells in response to DHT or R1881 (red arrows). Note increased ARBS2/ARBS3 binding as well in some conditions such as VCaP_CDSS-24h stimulated by DHT plus DEX (c4).

(**d-g**) AR activity (AR-A) and *BCL-2* mRNA levels in VCaP and VCaP_Enza-3w cells treated with DHT or DEX in GSE214756. (**d**) Dot plot showing AR-A score in VCaP and VCaP_Enza-3w cells under ±DHT and ±DEX conditions. One-way ANOVA with Tukey *post hoc* testing (right; **p*<0.05; ***p*<0.01, ****p*<0.001, *****p*<0.0001; ns, not significant) shows that the AR in regular VCaP (AD) cells is highly responsive to DHT stimulation (conditions 1 and 2). In addition, although DHT also induced AR-A in VCaP_Enza-3w cells, the induction was much lower than in VCaP-AD cells (conditions 4 vs. 2). Notably, the GR agonist DEX induced a more pronounced upregulation in AR-A than DHT in VCaP_Enza-3w cells (conditions 8 vs. 4), indicating the significance of GR-mediated signaling in castrated VCaP cells. (**e**) Bar plot showing reduced *BCL-2* mRNA in VCaP_Enza-3w cells (in comparison to VCaP-AD cells). (**f**) DEX upregulates AR-A in both VCaP and VCaP_Enza-3w cells. (**g**) DEX-induced *BCL-2* upregulation in VCaP but not VCaP_Enza-3w cells. In **e-g**, ***p*<0.01, ****p*<0.001, and *****p*<0.0001 (Student’s *t*-test).

(**h-m**) Involvement of ARv7 in repressing BCL-2 in VCaP-AI. (**h**) Dot plots showing correlations between and among the mRNA levels of *BCL-2*, *AR*, and *AR-v7* and with AR-A in the mCRPC SU2C 2019 cohort. *BCL-2* negatively correlated with *AR* (r = –0.37), *ARv7* (r = –0.31) and AR-A (r = –0.34), all *p* < 0.0001. *ARv7* positively correlated with *AR* mRNA (r = 0.75, *p* < 0.0001). These relationships indicate that higher AR and ARv7 signaling associates with lower *BCL-2* in advanced PCa. (**i**) Re-analysis of GSE252841 RNA-seq data^97^ showing *BCL-2* mRNA levels in VCaP16 cells after siRNA knockdown of AR isoforms. Cells were treated with siNTC (non-targeting control), siEX1 (targeting AR-FL and ARv7), siEX7 (targeting AR-FL only), or siARv7 (targeting ARv7 only). Data were normalized to siNTC and presented as FC (**p*<0.05; ***p*<0.01, ****p*<0.001, *****p*<0.0001; one-way ANOVA with multiple comparisons). (**j**) ARv7 ChIP-seq tracks in VCaP and Enza-resistant VCaP16 cells showing minimal ARv7 occupancy at the *BCL-2* genomic region in most conditions but with slightly enhanced ARBS1 binding in VCaP16_DHT-4h cells. (**k**) ARv7-specific PROTAC degrader downregulates *ARv7* but upregulates *BCL-2* mRNA levels. Regular VCaP cells were cultured in CDSS for one week to derive VCaP-AI cells which were then exposed to the V7 PROTAC (0.5 µM) or DMSO (vehicle control) for 24 h. Bar graphs show relative expression of *ARv7* and *BCL-2* plotted as fold change (FC) over the DMSO control (**p*<0.05 and ****p*<0.001 when compared to DMSO; one-sample Student’s *t*-test). (**l**) WB analysis of AR, ARv7, and BCL-2 in our VCaP-AI cells (above) after siRNA or PROTAC-mediated targeting of AR and/or ARv7. Cells in the

siAR group were treated with AR siRNA^20^ for a total of 48 h whereas the ARv7-directed PROTAC was applied for 24 h. For the first condition (NTC/DMSO), VCaP-AI cells were first transfected with siNTC for 24 h (control for siAR cells) and then treated with DMSO for an additional 24 h (control for ARv7 Protac). (**m**) Re-analyzing GSE252841 RNA-seq data^97^ (unpaired Student’s *t*-test) revealed that in Enza-resistant VCaP16 cells, a PROTAC AR-FL Degrader (ARD) surprisingly downregulated (rather than upregulated) *BCL-2* mRNA levels.

1. GR ChIP-seq tracks in the indicated VCaP cells showing no detectable GR binding to the *BCL-2* genomic region under DEX alone or combined DEX+DHT conditions.
2. FOXA1 ChIP-seq tracks around the ARBSs of the *BCL-2* genomic region in the indicated VCaP cells.

(**p-q**) Hypothesized mechanisms of *BCL-2* expression in VCaP-AD vs. VCaP-AI models. See Text for discussions. AD, androgen-dependent; AI, androgen-independent; ARE, androgen-responsive elements; ARfl, AR-FL; Co-R, co-repressor; FKHD, forkhead domain (motif); GR, glucocorticoid receptor; Pri-PCa, primary PCa; T, testosterone.

**
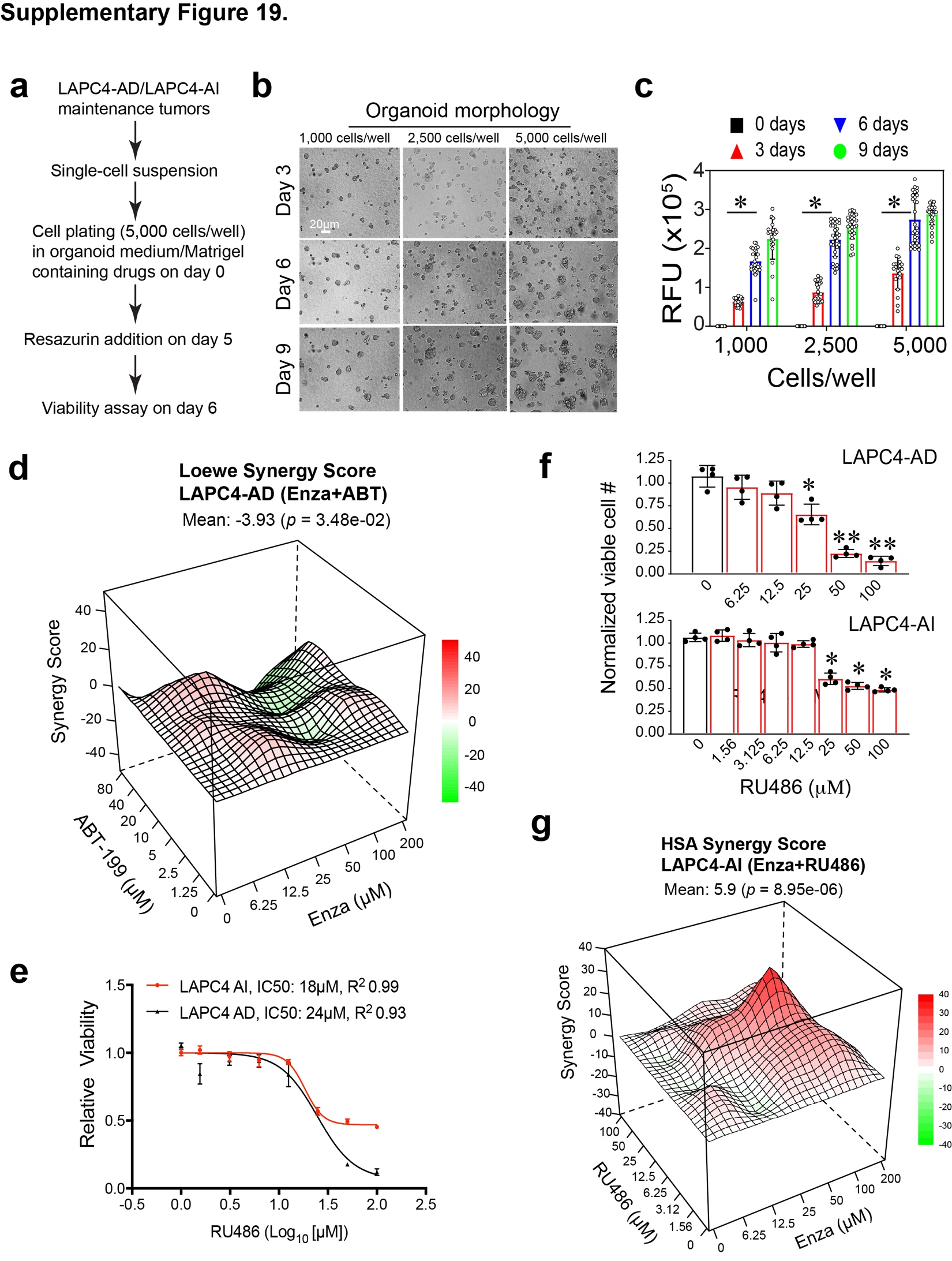
**

**Supplementary Figure 19. Effects of BCL2i ABT199, Enza and GR inhibitor RU486 on**

**LAPC4-AD/AI tumor derived organoids.**

(**a**) Schematic workflow for generation of organoids from LAPC4-AD and LAPC4-AI tumors.

(**b**) Representative brightfield images showing the LAPC4-AI organoid morphology at Days 3, 6, and 9 following plating at three different densities. Scale bar, 20 μm for all panels (illustrated in the left upper panel).

(**c**) Bar graphs presenting the cell growth (i.e., relative fluorescence units; RFU) for LAPC4-AI organoids seeded at different densities. Resazurin-based viability assay was used to assess organoid viability. A one-way analysis of variance (ANOVA) was performed to evaluate differences in viability across time points (0, 3, 6, and 9 days) and seeding densities (1000, 2500, and 5000 cells). Bonferroni-adjusted *post hoc* comparisons were applied to account for multiple testing. The error bars represent the standard deviation (SD) of multiple independent RFU measurements whereas the mean RFU was calculated as the arithmetic average of the replicate values, and the SD was determined using the standard sample standard deviation formula:

$$SD=\sqrt{\frac{\sum(X_{i}-\text{Mean})^{2}}{n-1}}$$

where $X_{i}$represents each individual RFU measurement and $n$ denotes the number of replicate wells.

(**d**) 3D dose-response matrix depicting percentage inhibition across Enza and ABT-199 concentration gradients in LAPC4-AD organoids.

(**e**) Dose-response curves of RU486 treatment in LAPC4-AD and LAPC4-AI organoids, showing relative viability as a function of RU486 concentration. IC50 values are indicated.

(**f**) Bar graphs show normalized viable cell numbers after treatment with increasing concentrations of RU486 in LAPC4-AD and LAPC4-AI organoids. Statistical significance was determined by unpaired two-tailed Student’s *t-*test; **p*< 0.05, ***p*< 0.01.

(**g**) Synergy score plots depicting the interaction between Enza and RU486 across different dose combinations in LAPC4-AI organoids. Positive synergy scores indicate synergistic effects.

**
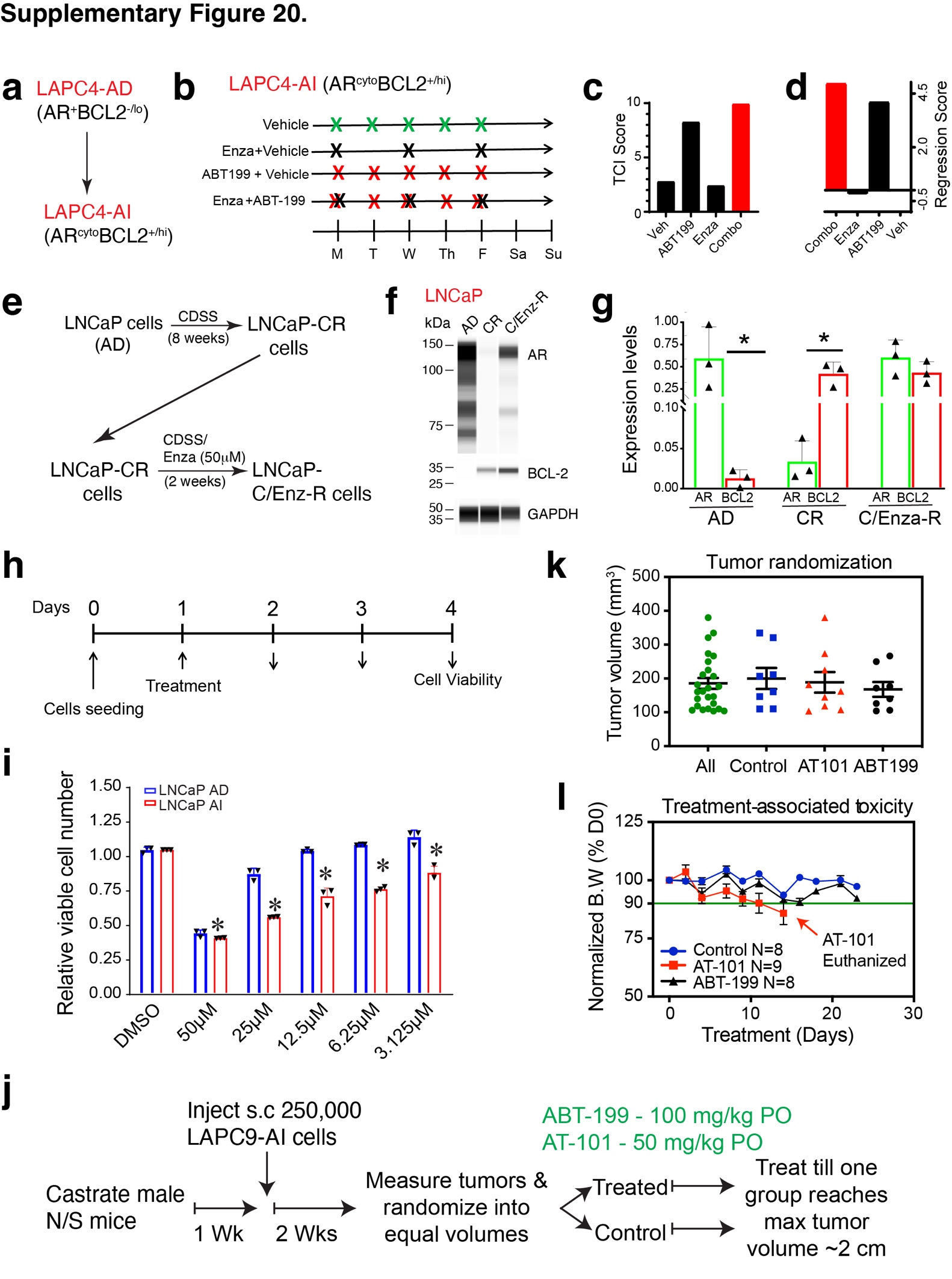
**

**Supplementary Figure 20. Therapeutic studies with the BCL-2i ABT-199 in 3 CRPC models.**

(**a**) Schematic of phenotypes of LAPC4-AD and LAPC4-AI CRPC models.

(**b**) Treatment schema for LAPC4-AI xenografts.

(**c**) Tumor control index (TCI) score showing superior inhibition of LAPC4-AI tumors by in Enza/ABT-199 combination.

(**d**) Tumor regression score showing superior response in the combination group.

(**e-i**) In vitro studies in the LNCaP-AD/AI progression models. (**e**) Schematic of generating LNCaP-AI progression models. (**f**) Immunoblot of AR and BCL-2 in parental LNCaP, LNCaP-CR, and LNCaP-C/Enza-R cells. GAPDH serves as loading control. (**g**) Densitometric quantification of AR and BCL-2 levels from (f), normalized to GAPDH. LNCaP-CR and LNCaP-C/Enza-R cells show reduced AR but increased BCL-2 (**p*<0.05). (**h**) Schematic of cell viability assays in LNCaP-AD/AI cell line models after ABT-199 treatment. (**i**) Cell viability assays showing LNCaP-AI cells were more sensitive to ABT-199 than LNCaP-AD cells (**p* < 0.05).

(**j-l**) Experiments in AR^-/lo^ LAPC9-AI model. (**j**) Workflow for treatment with BCL-2i in LAPC9-AI xenograft model. (**k**) Tumor volumes at the time of randomization across treatment arms. (**l**). Animal body weight over time showing minimal toxicity with ABT-199 but significant toxicity with AT-101.


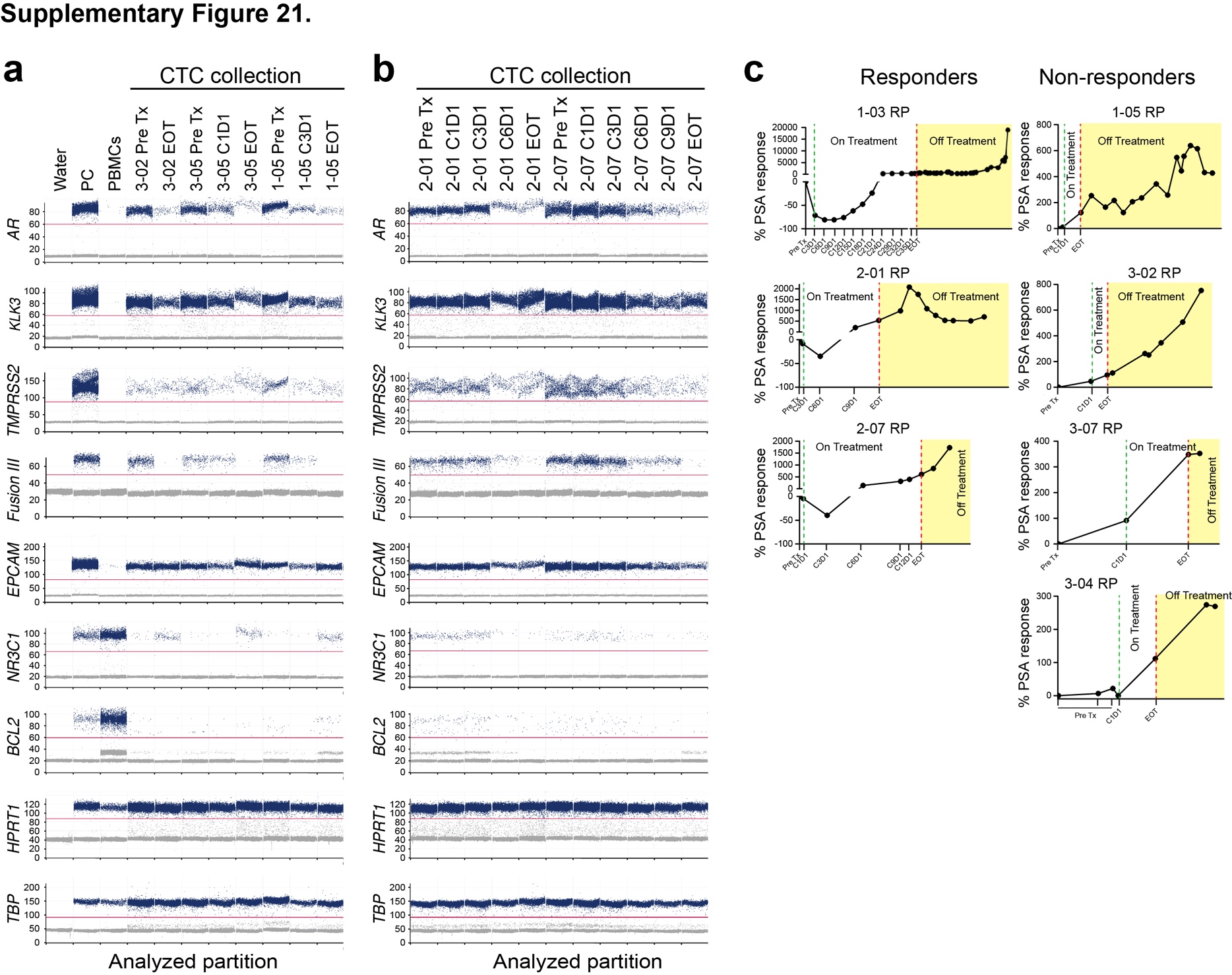


**Supplementary Figure 21. CTC gene expression dynamics in responders and non-responders.**

1. One-dimensional ddPCR fluorescence plots showing longitudinal expression patterns of target genes across sequential treatment timepoints in non-responder patients, highlighting minimal baseline BCL-2 expression and compensatory NR3C1 activation at the EOT samples.
2. One-dimensional ddPCR fluorescence plots showing longitudinal expression patterns of target genes across sequential treatment timepoints in the 2 responder patients, illustrating BCL-2 suppression and decreased CTC burden (i.e., TMPRSS2 and TMPRSS2-ERG fusion type III).
3. PSA responses in responders (left) and non-responders (right). PSA levels were measured over time and plotted as percentage change from baseline (baseline set to 0%) during treatment with enzalutamide plus venetoclax. Each panel represents an individual patient, grouped by clinical response. Black dots indicate PSA measurements at serial time points. Vertical green dashed lines mark the start of combination therapy and red dashed lines indicate treatment discontinuation. The yellow-shaded region represents the off-treatment phase.

**Supplementary Table 1: Summary of ChIP-seq, bulk and single-cell RNA-seq, and ATAC-seq datasets used to profile AR binding, transcriptional changes, and chromatin accessibility across prostate cancer models and treatment conditions.**

| **#** | **Figures** | **Item** | **Histology (Sample Type)** | **Data Type/Data Source** | **PMID** | **Publication** |
| --- | --- | --- | --- | --- | --- | --- |
| **1** | Fig.1a | BCL2 mRNA in TCGA PRAD | Primary PCa (Pri-PCa) | RNA-seq/Xena portal | 26544944 | Abeshouse A et al., Cell 2015;163(4):1011-1025 |
| **2** | Fig.1b | BCL2 family mRNAs in 7 matched pre-/post-nADT prostate cancer pairs from Rajan et al., 2014 | PCa (nADT) | RNA-seq/GSE48403 | 24054872 | Rajan P et al., Eur Urol. 2014;66(1):32-39 |
| **3** | Fig.1b | BCL2 family mRNAs in 20 matched pre-/post-nADT prostate cancer pairs from Sharma et al., 2018 | PCa (nADT) | RNA-seq/GSE111177 | 30314329 | Sharma NV et al., Cancers (Basel). 2018;10(10). |
| **4** | Fig.1b | BCL2 family mRNAs in post-nADT vs. matched untreated PCa (n=43 each), Roswell cohort (Nastiuk & Chatta) | PCa (nADT) | RNA-seq/Nastiuk | unpublished | Jamroze A et al. (Chatta G., Nastiuk KL) 2024 (Submitted) |
| **5** | Fig.1b | BCL2 family mRNAs in CRPC tumors post- vs. pre-enzalutamide treatment (n=21 pairs) from Alumkal et al., 2022 | mCRPC | RNA-seq/PMID: 36109521 (supplementary) | 36109521 | Westbrook TC et al., Nat commun. 2022,13(1): 5345. |
| **6** | Fig.1c & Fig. S1b-f | BCL2 family mRNAs (scRNA-seq) in major PCa-related cell populations from FACS-purified normal human prostate epithelial cell subpopulations (3: basal, luminal, proximal luminal), Pri-PCa (2), and CRPC (3) | Normal Prostate, Pri-PCa, CRPC-NE, mCRPC | scRNA-seq/PRJNA699369 | 35058087 | Cheng Q et al., Eur Urol. 2022, 81(5):446-455. |
| **7** | Fig.1d | BCL2 family mRNAs in PCa cell lines LNCaP-ARKO (6) vs. AR (4) (in vitro) | PCa cell lines | RNA-seq/Tang | unpublished | unpublished |
| **8** | Fig.1d | BCL2 family mRNAs in PCa cell lines LNCaP-ARKO (7) vs. AR (7) (in vivo, castr) | PCa cell lines | RNA-seq/Tang | unpublished | unpublished |
| **9** | Fig.1d | BCL2 family mRNAs in PCa Xenografts LNCaP Pri CRPC (4) vs. AD (4) | Xenografts | RNA-seq/GSE88752 | 30190514 | Li Q et al., Nat Commun. 2018;9(1):3600 |
| **10** | Fig.1d | BCL2 family mRNAs in PCa Xenografts LNCaP Sec CRPC (4) vs. AD (4) | Xenografts | RNA-seq/GSE88752 | 30190514 | Li Q et al., Nat Commun. 2018;9(1):3600 |
| **11** | Fig.1d | BCL2 family mRNAs in PCa Xenografts LNCaP Sec CRPC (4) vs. Pri CRPC (4) | Xenografts | RNA-seq/GSE88752 | 30190514 | Li Q et al., Nat Commun. 2018;9(1):3600 |
| **12** | Fig.1d | BCL2 family mRNAs in PCa Xenografts LAPC9 CRPC (5) vs. AD (5) | Xenografts | RNA-seq/GSE88752 | 30190514 | Li Q et al., Nat Commun. 2018;9(1):3600 |
| **13** | Fig. 5a | AR activity, BCL2 mRNA and their correlation in 7 matched pre-/post-nADT prostate cancer pairs from Rajan et  al., 2014 | PCa (nADT) | RNA-seq/GSE48403 | 24054872 | Rajan P et al., Eur Urol. 2014;66(1):32-39 |
| **14** | Fig. 5a | AR activity, BCL2 mRNA levels, and their correlation in 7 matched pre-/post-nADT prostate cancer samples  from the responder subgroup (referred to as the 'Low Impact Group') in Sharma et al., 2018 | PCa (nADT) | RNA-seq/GSE111177 | 30314329 | Sharma NV et al., Cancers (Basel). 2018;10(10). |
| **15** | Fig. 5a | AR activity, BCL2 mRNA, and their correlation in 6 matched pre-/post-nADT prostate cancer pairs from Long et  al., 2020 | PCa (nADT) | RNA-seq/GSE150368 | 32951005 | Long X et al., Cell Death Dis. 2020;11(9):779. |
| **16** | Fig. 5b | AR activity, BCL2 mRNA, and their correlation in 25 PCa patients treated for greater than 2 months with nADT vs. matched untreated PCa (n=25), Roswell cohort (Nastiuk & Chatta) | PCa (nADT) | RNA-seq/Nastiuk | unpublished | Jamroze A et al. (Chatta G., Nastiuk KL) 2024 (Submitted) |
| **17** | Fig. 5c | AR and BCL2 mRNA and their correlation in Patient CRPC (n=40) from Tang et al., 2022 | mCRPC | RNA-seq/GSE199190, GSE181374 | 35617398 | Tang F et al. Science, 2022, 376(6596): eabe1505. |
| **18** | Fig. 5h-i | AR ChIP-seq in PCa cells LNCaP-AD (GSM699631) and LNCaP-AI (GSM699630) | PCa cell lines | AR ChIP-seq/GSE28264 | 22083957 | Tan PY et al., Mol Cell Biol. 2012;32(2):399-414 |
| **19** | Fig. S1a | BCL2 mRNA in normal prostate | Normal Prostate | RNA-seq/PMID: 27926864 (supplementary) | 27926864 | Liu X et al., Cell Rep 2016 |
| **20** | Fig. S1a | BCL2 mRNA in normal prostate | Normal Prostate | Microarray/GSE89050 | 27926864 | Liu X et al., Cell Rep 2016 |
| **21** | Fig. S8a | BCL2 mRNA in Pri-PCa and tumor adjacent benign prostate (n=52) from TCGA_PRAD | Pri-PCa and adjacent benign prostate | RNA-seq/Xena portal | 26544944 | Abeshouse A et al., Cell 2015;163(4):1011-1025 |
| **22** | Fig. S8b | BCL2 mRNA in Pri-PCa (n=131) and tumor adjacent benign prostate (n=29) from Taylor et al., 2010 | Pri-PCa and adjacent benign prostate | Microarray/GSE21034; cBioPortal | 20579941 | Taylor BS et al., Cancer Cell 2010;18(1):11-22. |
| **23** | Fig. S14a | AR activity, BCL2 family mRNA levels, and their correlation in 7 matched pre-/post-nADT prostate cancer pairs from Rajan et al., 2014 | PCa (nADT) | RNA-seq/GSE48403 | 24054872 | Rajan P et al., Eur Urol. 2014;66(1):32-39 |
| **24** | Fig. S14b | AR activity, BCL2 family mRNA levels, and their correlation in 7 matched pre-/post-nADT prostate cancer samples from the responder subgroup (referred to as the 'Low Impact Group') in Sharma et al., 2018 | PCa (nADT) | RNA-seq/GSE111177 | 30314329 | Sharma NV et al., Cancers (Basel). 2018;10(10). |
| **25** | Fig. S14c | AR activity, BCL2 family mRNA levels, and their correlation in 6 matched pre-/post-nADT prostate cancer pairs from Long et al., 2020 | PCa (nADT) | RNA-seq/GSE150368 | 32951005 | Long X et al., Cell Death Dis. 2020;11(9):779. |
| **26** | Fig. S14d | AR activity, BCL2 mRNA, and their correlation in 25 PCa patients treated for greater than 2 months with nADT vs. matched untreated PCa (n=25), Roswell cohort (Nastiuk & Chatta) | PCa (nADT) | RNA-seq/Nastiuk | unpublished | Jamroze A et al. (Chatta G., Nastiuk KL) 2024 (Submitted) |
| **27** | Fig. S14e | AR activity, BCL2 family mRNA levels, and their correlations in 21 matched pre-/post-enzalutamide treatment CRPC tumor pairs from Westbrook et al., 2022 (Alumkal pre-/post-Enza cohort) | mCRPC | RNA-seq/PMID: 36109521 (supplementary) | 36109521 | Westbrook TC et al., Nat commun. 2022,13(1): 5345. |
| **28** | Fig. S15a | AR mRNA, AR activity, BCL2 family mRNA levels, and their correlations in Patient CRPC (n=40) from Tang et al., 2022 | mCRPC | RNA-seq/GSE199190, GSE181374 | 35617398 | Tang F et al. Science, 2022, 376(6596): eabe1505. |
| **29** | Fig. S15b, c | AR activity, BCL2 mRNA and their correlation in PCa Xenografts LNCaP Sec CRPC AI (4) vs. AD (4) | Xenografts | RNA-seq/GSE88752 | 30190514 | Li Q et al., Nat Commun 2018; 9(1):3600 |
| **30** | Fig. S15d, e | AR activity, BCL2 mRNA and their correlation in PCa Xenografts LAPC9 CRPC AI (5) vs. AD (5) | Xenografts | RNA-seq/GSE88752 | 30190514 | Li Q et al., Nat Commun 2018; 9(1):3600 |
| **31** | Fig. S16a | AR ChIP-seq in BCL2 region from PCa cell line LNCaP | PCa cell line | AR ChIP-seq/GSE85558 | 29153843 | Shukla S et al. Cancer Cell 2017; 32(6):792-806.e7. |
| **32** | Fig. S16a | AR ChIP-seq in BCL2 region from PCa cell line LNCaP-abl | PCa cell line | AR ChIP-seq/GSE80238 | 35031563 | Liao et al., PNAS 2022 |
| **33** | Fig. S16a | AR ChIP-seq in BCL2 region from PCa cell line LNCaP-BicR | PCa cell line | AR ChIP-seq/GSE66037 | 26404510 | Takayama K et al. Nat Commun 2015 |
| **34** | Fig. S16a | AR ChIP-seq in BCL2 region from PCa cell line C4-2 | PCa cell line | AR ChIP-seq/GSE65066 | 27068475 | Zhao Y et al. Cell Rep 2016 |
| **35** | Fig. S16a | AR ChIP-seq in BCL2 region from PCa cell line C4-2B | PCa cell line | AR ChIP-seq/GSE72714 | 27019329 | Wang J et al. Nat Med 2016; 22(5):488-96. |
| **36** | Fig. S16b-g | AR ChIP-seq from primary PCa tissue or PDX derived from CRPC Patients from Baca et al. 2022, with LNCaP-AD/AI data (adapted from Figure 5g) shown above for alignment. | Pri-PCa and CRPC | AR ChIP-seq/GSE130408 | 36071171 | Baca SC et al. Nat Genet 2022; 54(9):1364-1375. |
| **37** | Fig. S17a-d | BCL2 ATAC-seq, BCL2 RNA-seq and their correlation in Patient CRPC (ATAC_CRPC_AR (4);  ATAC_CRPC_SCL (2); ATAC_CRPC_Wnt (4); ATAC_CRPC_NE (2)) from Tang et al., 2022 | mCRPC | ATAC-seq, RNA-seq/GSE199190 | 35617398 | Tang F et al. Science, 2022, 376(6596): eabe1505. |
| **38** | Fig. S18b | AR ChIP-seq in PCa cells LNCaP-AD (GSM699631), LNCaP-AI (GSM699630), and LNCaP genomic input  (GSM715395), with LNCaP-AD/AI data (adapted from Figure 5g) shown above for alignment. | PCa cell lines | AR ChIP-seq/GSE28264 | 22083957 | Tan PY et al., Mol Cell Biol. 2012;32(2):399-414 |
| **39** | Fig. S18c1 | AR-ChIPseq in VCaP with different conditions: VCaP-AD-regular medium, knockdown of siERG in regular  medium, and CDSS-48h_R1881. | PCa cell lines | AR ChIP-seq/GSE14092 | 20478527 | Yu J et al., Cancer Cell 2010 |
| **40** | Fig. S18c2 | GR-ChIPseq in VCaP in DHT condition. | PCa cell lines | RN-seq/GSE39654; AR ChIP-  seq/GSE30624; GSE39879 | 21915096;  3269278 | Sahu B, et al., EMBO J. 2011; Cancer Res. 2013 |
| **41** | Fig. S18c3 | AR-ChIPseq in VCaP with different conditions: CDSS-48h_Veh-6h, CDSS-48h_Veh-6h_DHT-12h, and CDSS-  48h_Enza-6h_DHT-12h. | PCa cell lines | AR ChIP-seq/GSE55062 | 24759320 | Asangani IA et al., Nature 2014. |
| **42** | Fig. S18c4, S18n,  S18o | AR-ChIPseq, GR-ChIPseq, FOXA1-ChIPseq in VCaP with different conditions | PCa cell lines | AR-/GR-/FOXA1-  ChIPseq/GSE266213;GSE266217 | 38015476;  40456604 | Hiltunen J et al., Genome Res 2025; Helminen L et al.,  NAR 2024 |
| **43** | Fig. S18c5, S18i,  S18j | AR- & ARv7-ChIPseq in VCaP, VCaP16; RNAseq in VCaP16 with different conditions, including knockdown of  siAR-v7. | PCa cell lines | AR, ARv7-ChIP-seq/GSE252897; RNA-  seq/GSE252841 | 39709604 | Poluben et al., Cell Rep 2025. |
| **44** | Fig. S18d-g | RNA-seq in VCaP with different conditions | PCa cell lines | RNA-seq//GSE214756 | 38015476 | Helminen L et al., NAR 2024 |
| **45** | Fig. S18h | AR-v7, AR, AR activitiy, BCL2 mRNA, and their correlation in mCRPC (SU2C 2019) | mCRPC | RNA-seq/cBioPortal | 31061129 | Abida W et al., PNAS 2019;116(23):11428-11436. |

**Supplementary Table 2. Information on antibodies used in the current study.**

| **Antibody** | **Source** | **Reactivity** | **Company** | **Catalog #** | **Clone** | **Usage** | **Dilution** |
| --- | --- | --- | --- | --- | --- | --- | --- |
| AR | Rabbit mAb | Human | CST | 8938S | D6F11 | Vectra | 1/200 |
| BCL-2 | Mouse mAb | Human | abcam | ab692 | 100/D5 | Vectra | 1/100 |
| Cytokeratin | Mouse mAb | All species | Dako | #M3515 | AE1/AE3 | Vectra | 1/100 |
|  |  |  |  |  |  |  |  |
| AR-^156^Gd | Rabbit | Human | Cell Signaling | 5153 | D6F11 | IMC | 1/100 |
| BCL2-^146^Nd | Mouse | Human | Biolegend | 658701 | 100 | IMC | 1/100 |
| Pan-CK-^141^Pr | Mouse | Human, Rat | Biolegend | 914204 | AE1/AE3 | IMC | 1/100 |
|  |  |  |  |  |  |  |  |
| A1/Bfl-1 | Rabbit mAb | All species | Cell signaling | #14093 | D1A1C | WB | 1/1000 |
| Apoptosis cocktail | Rabbit | Human | Abcam | ab136812 | - | WB | 1/1000 |
| AR | Mouse mAb | Human | Santa Cruz | sc07305 | Clone 441 | WB | 1/1000 |
| AR | Rabbit mAb | Human | CST | 8938S | D6F11 | WB | 1/1000 |
| ARv7 | Mouse mAb | Human | Proteintech | AG10008 | AG10008 | WB | 1/1000 |
| BCL-2 | Mouse mAb | Human | Cell signaling | #15071 | 124 | WB | 1/1000 |
| BCL-W | Rabbit pAb | H, M, R | LS bio | LS-C382259 |  | WB | 1/1000 |
| BCL-xL | Rabbit mAb | H, M, R, Mk | Cell signaling | #2764 | 54H6 | WB | 1/1000 |
| Cleaved Caspase-3 | Rabbit | H M R Mk | Cell signaling | #9661 | - | WB | 1/1000 |
| Caspase-3 | Rabbit pAb | H M R Mk | Cell signaling | #9662 | Asp175 | WB | 1/1000 |
| β-Actin | Rabbit mAb | H,M,R,Mk,Pg | CST | 5125 | 13E5 | WB | 1/1000 |
| FKBP5 | Rabbit pAb | Human | CST | 8245 |  | WB | 1/1000 |
| GR | mouse mAb | Human | BD Transduction Labs | 611227 | Clone 41 | WB | 1/1000 |
|  |  |  |  |  |  |  |  |
| MCL-1 | Rabbit mAb | Human | CST | 5453 | D35A5 | WB | 1/1000 |
| BCL-2 | Mouse | Human | Biolegend | 658701 | 100 | IF | 1/150 |
| AR Alexa Fluor® 647 Conjugate | Rabbit mAb | Human | CST | #7397 | D6F11 | IF | 1/100 |
| AR | Rabbit mAb | Human | CST | 8938S | D6F11 | IHC | 1/500 |

*Abbreviations: mAb, monoclonal antibody; pAb, polyclonal antibody.

**Supplementary Table 3: Information available on patient samples in the TMAs and whole-mount (WM) sections.**

|  | **Source** | **Subject ID/# of Patients** | **Tissue type** | **Treatment** |
| --- | --- | --- | --- | --- |
| **Whole Mount** | **UCLA (for CRPC) and Tang Lab (for HPCa)** | U12-7606-CRPC | Prostate | LHRH agonist and bicalutamide |
|  |  | U13-01316-CRPC | Prostate | LHRH agonist |
|  |  | U13-6707-CRPC | Prostate | LHRH agonist |
|  |  | S13-13553-CRPC | Prostate | Abiraterone and enzalutimide |
|  |  | S13-19900-CRPC | Prostate | LHRH agonist |
|  |  | HPCa14N | Prostate | No Treatment |
|  |  | HPCa18N | Prostate | No Treatment |
|  |  | HPCa21N | Prostate | No Treatment |
|  |  | HPCa28N | Prostate | No Treatment |
|  |  | HPCa27T | Prostate | No Treatment |
|  |  | HPCa31T | Prostate | No Treatment |
|  |  | HPCa33T | Prostate | No Treatment |
| **CRPC TMA** | **UCLA CRPC TMA (20**  **CRPC samples /40 cores)** | CRPC-1 | Prostate | LHRH agonist |
|  |  | CRPC-2 | Prostate | LHRH agonist |
|  |  | CRPC-3 | Prostate | LHRH agonist |
|  |  | CRPC-4 | Prostate | LHRH agonist |
|  |  | CRPC-5 | Prostate | LHRH agonist |
|  |  | CRPC-6 | Prostate | LHRH agonist |
|  |  | CRPC-7 | Prostate | LHRH agonist |
|  |  | CRPC-8 | Prostate | LHRH agonist |
|  |  | CRPC-9 | Prostate | LHRH agonist |
|  |  | CRPC-10 | Prostate | LHRH agonist |
|  |  | CRPC-11 | Prostate | LHRH agonist |
|  |  | CRPC-12 | Prostate | LHRH agonist |
|  |  | CRPC-13 | Prostate | LHRH agonist |
|  |  | CRPC-14 | Prostate | Radiation, LHRH agonist, and bicalutamide |
|  |  | CRPC-15 | Prostate | Radiation, LHRH agonist, and bicalutamide |
|  |  | CRPC-16 | Prostate | Radiation, LHRH agonist, and bicalutamide |
|  |  | CRPC-17 | Prostate | Radiation, LHRH agonist, and bicalutamide |
|  |  | CRPC-18 | Prostate | Radiation and cryotherapy |
|  |  | CRPC-19 | Prostate | LHRH agonist (2 weeks) |
|  |  | CRPC-20 | Prostate | LHRH agonist (4 mo) + bicalutamide (2 mo) |
| **TMA- GL-115** | **Jiaoti Huang** | 115 (N+T) | Prostate | No Treatment |
| **Xenograft TMA 22** | **Tang Lab** | 4 AD-AI pairs | Xenografts | Intact+Castartion |
| **Xenograft TMA 23** | **Tang Lab** | 4 AD-AI pairs | Xenografts | Intact+Castartion |

*Presented is available patient/sample information for 2 TMA sets, and 12 whole mount (WM), N/T and CRPC samples. The CRPC TMA contained a total of 40 cores derived from duplicate sections of patient samples and the TMA-GL-115 contained triplcate cores, respectively, from the tumor (T) and corresponding adjacent benign (N) tissues of the 115 treatment-naive PCa. The UCLA whole-mount sections were from the prostate (radical prostatectomy or TURP). Note that virtually all samples were collected from patients treated decades ago (mostly with LHRH agonists) before the introduction of new generation antiandrogens; only one patient (whole mount S13-13553) was treated with abiraterone and enzalutamide. De-identified patient samples were used and detailed treatment information for most patients is unavailable. For more information on these specimens, please refer to Li Q et al., *Nat Commun* . 2018 and Li Q. et al., *Nat Commun* . 2019.

**Supplementary Table 4. List of major reagents utilized in this study.**

| **Chemical/Reagent/Kit Name** | **Company** | **Catalog #** |
| --- | --- | --- |
| Enzalutamide | Selleck Chemicals | S1250 |
| Enzalutamide | Apex Bio | A3003 |
| ABT-199 | Apex Bio | A8194 |
| PROTAC AR-V7 degrader-1 | MCE | HY-145479 |
| Trypan Blue Solution | Thermo | 15250061 |
| Cultrex Poly-L-Lysin | R&D | 3438-200-01 |
| IMDM media | Gibco™ | 12440053 |
| DMEM Media | Gibco™ | 11965092 |
| IMDM no phenol red | Gibco™ | 21056023 |
| 191/193Ir DNA Intercalator | Fluidigm | 201192B |
| Paraformaldehyde | VWR | PI28908 |
| Ethylenediaminetetraacetic acid (EDTA) | Fisher Scientific | BP118500 |
| Maxpar 10x Barcode Perm Buffer | Fluidigm | 201057 |
| Puromycin | Sigma-Aldrich | P7255 |
| Mito view Green | Biotium | 70054 |
| Prolong Gold antifade with DAPI | Thermo | P36941 |
| Western Lightning Plus-ECL | Perkin Elmer | NEL104001 |
| Wes EZ standard pack 12-230 kDa | Protien Simple | PS-ST01EZ-8 |
| Wes EZ standard pack 2-40 kDa | Protien Simple | PS-ST05EZ-8 |
| Wes antibody diluent 2 | Protien Simple | 042-205 |
| Wes Anti-mouse secondary | Protien Simple | 042-205 |
| Wes Anti-rabbit secondary | Protien Simple | 042-206 |
| Wes Anti-goat secondary HRP | Protien Simple | 043-522 |
| Wes peroxide | Protien Simple | 043-379 |
| Wes streptavidin HRP | Protein Simple | 042-414 |
| Wes Luminol-S | Protien Simple | 043-311 |
| Western Lightning ECL Pro | Perkin Elmer | NEL12001 |
| Trizol reagent | Thermo | 15596018 |
| BSA | Cell Signaling | 9998 |
| BD Pharmingen™ FITC Annexin V Apoptosis Detection Kit II | BD Pharmingen | 556570 |
| LIVE/DEAD Cell Imaging Kit (488/570) | Thermo Fisher Scientific | R37601 |
| Firefly & Renilla Luciferase Single Tube Assay Kit, 150 assays | OriGene | PR300008 |
| Resazurin Assay Kit (Cell Viability) | Abcam | ab129732 |
| ChIP-IT® qPCR Analysis Kit | Active Motif | 5302 |
| SsoAdvanced Universal Probes Supermix | Bio-Rad | 1725280 |
| SuperScript™ III Reverse Transcriptase | Thermo Fisher Scientific | 18080093 |
| ChIP-IT High Sensitivity® Kit | Active Motif | 53040 |
| High Sensitivity Chromatin Preparation | Active Motif | 53046 |
| Arcturus RiboAmp HS PLUS RNA Amplification Kit (24 samples) | Thermo Fisher Scientific | KIT0505 |
| PicoPure RNA Isolation Kit (40 samples) | Thermo Fisher Scientific | KIT0204 |

**Sequences and specifications of qPCR and ddPCR primers and probes used**

| **Probe ID** | **Catalog number** | **Assay ID** |
| --- | --- | --- |
| PrimePCR™ Probe Assay: MCL1, Human | 12001950 | qHsaCEP0052441 |
| PrimePCR™ Probe Assay: BCL2L1, Human | 12001950 | qHsaCEP0039517 |
| PrimePCR™ Probe Assay: BCL2, Human | 12001950 | qHsaCEP0058350 |
| PrimePCR™ Probe Assay: TBP, Human | 10031228 | qHsaCIP0036255 |
| PrimePCR™ Probe Assay: BCL2L2, Human | 12001950 | qHsaCEP0025467 |
| PrimePCR™ Probe Assay: HPRT1, Human | 10031228 | qHsaCIP0030549 |
| PrimePCR™ Probe Assay: AR human | 10025536 | qHsaCIP0026366 |
| PrimePCR™ Probe Assay: KLK3 human | 10025543 | qHsaCEP0051088 |
| PrimePCR™ Probe Assay:TMPRSS2 human | 10025544 | qHsaCEP0051087 |
| PrimePCR™ Probe Assay:BCL2L1, human | 10025545 | qHsaCEP0039517 |
| PrimePCR™ Probe Assay:NR3C1, human | 10025546 | qHsaCEP0050768 |
| PrimePCR™ Probe Assay:AMACR, human | 10025547 | qHsaCIP0029215 |
| PrimePCR™ Probe Assay:EPCAM, human | 10025548 | qHsaCEP0051089 |
| PrimePCR™ Probe Assay:TBP, human | 10025549 | qHsaCIP0036255 |
| PrimePCR™ Probe Assay:HPRT1, human | 10025550 | qHsaCIP0030549 |
| PrimePCR™ Probe Assay:PTPRC, human | 10025551 | qHsaCEP0041630 |
| PrimePCR™ Probe Assay: ARV7, human | Custom design | Probe Sequence: TGCTTGCAATTGCCAA CCCGG |

**ChIP-qPCR primers/probe sequences**

| **Binding site#** | **Part #** | **Sequences** |
| --- | --- | --- |
| ARBS1 | 10031276 | Forward primer: ACAAGTTGCACGTGTGTATT |
|  |  | Reverse primer: CCCAATAATCCAGTGTCCCT |
|  |  | Probe sequence: AGTAAGCCGCTGTGCTTCTAGAAG |
| ARBS2 | 10031276 | Forward primer: TGGACAAGACGGTTTGTAAGA |
|  |  | Reverse primer: AACAGAACGAGGTACAGATCA |
|  |  | Probe sequence: TGTCTCTGTGGCATCTAACAGCGTF |
| ARBS3 | 10031276 | Forward primer: CACACACACACACGAAGGAT |
|  |  | Reverse primer: CTAAGGTTACGAGCTGAGCC |
|  |  | Probe sequence: AGTTCATGAGAGACTGGCTTGCTTGAF |
| Neg control seq | 10031279 | Forward primer: GTGGCTACCTAGGACTGG |
|  |  | Reverse primer: TGACATCTGTTTCCAGAACTT |
|  |  | Probe sequence: TTCATCATTCCAAATGGAAACTCTACCCAF |

**Supplementary Table 5. Fold Change and 95% Confidence Intervals for BCL-2 Family Genes in Clinical and Preclinical Datasets (Figure 1 Panels)**

| **Associated Figure** | **Dataset** | **Gene** | **Aliases** | **Ensembl ID** | **FC (95% CI)** | **p-value** | **False Dicovery Rate (FDR)** | **Note** |
| --- | --- | --- | --- | --- | --- | --- | --- | --- |
| Figure 1b | Clinical Dataset #1 | *BCL2* | BCL-2 | ENSG00000171791 | 2.40 (1.61, 3.58) | 1.65E-05 | 7.87E-04 | post vs pre-nADT (n=7)/Rajan |
| Figure 1b | Clinical Dataset #1 | *BCL2L1* | BCL-XL | ENSG00000171552 | 0.68 (0.54, 0.86) | 1.02E-03 | 1.53E-02 | post vs pre-nADT (n=7)/Rajan |
| Figure 1b | Clinical Dataset #1 | *MCL1* | MCL-1 | ENSG00000143384 | 1.18 (0.86, 1.61) | 3.13E-01 | 6.04E-01 | post vs pre-nADT (n=7)/Rajan |
| Figure 1b | Clinical Dataset #1 | *BCL2L2* | BCL-W | ENSG00000129473 | 0.72 (0.56, 0.92) | 9.88E-03 | 7.28E-02 | post vs pre-nADT (n=7)/Rajan |
| Figure 1b | Clinical Dataset #1 | *BCL2A1* | A1/BFL1 | ENSG00000140379 | 1.26 (0.73, 2.20) | 4.08E-01 | 6.86E-01 | post vs pre-nADT (n=7)/Rajan |
| Figure 1b | Clinical Dataset #2 | *BCL2* | BCL-2 | ENSG00000171791 | 5.13 (12.71, 2.07) | 4.17E-04 | 2.26E-03 | post vs pre-nADT (n=20)/Sharma |
| Figure 1b | Clinical Dataset #2 | *BCL2L1* | BCL-XL | ENSG00000171552 | 1.56 (3.31, 0.73) | 2.51E-01 | 3.56E-01 | post vs pre-nADT (n=20)/Sharma |
| Figure 1b | Clinical Dataset #2 | *MCL1* | MCL-1 | ENSG00000143384 | 2.72 (4.72, 1.57) | 3.65E-04 | 2.09E-03 | post vs pre-nADT (n=20)/Sharma |
| Figure 1b | Clinical Dataset #2 | *BCL2L2* | BCL-W | ENSG00000129473 | 1.15 (3.11, 0.42) | 7.84E-01 | 8.43E-01 | post vs pre-nADT (n=20)/Sharma |
| Figure 1b | Clinical Dataset #2 | *BCL2A1* | A1/BFL1 | ENSG00000140379 | 3.55 (11.98, 1.06) | 4.06E-02 | 9.24E-02 | post vs pre-nADT (n=20)/Sharma |
| Figure 1b | Clinical Dataset #3 | *BCL2* | BCL-2 | ENSG00000171791 | 1.32 (1.13, 1.55) | 7.72E-04 | 9.83E-03 | nADT vs control (n=43)/Roswell |
| Figure 1b | Clinical Dataset #3 | *BCL2L1* | BCL-XL | ENSG00000171552 | 0.81 (0.69, 0.95) | 8.95E-03 | 4.95E-02 | nADT vs control (n=43)/Roswell |
| Figure 1b | Clinical Dataset #3 | *MCL1* | MCL-1 | ENSG00000143384 | 0.80 (0.70, 0.92) | 8.77E-01 | 9.34E-01 | nADT vs control (n=43)/Roswell |
| Figure 1b | Clinical Dataset #3 | *BCL2L2* | BCL-W | ENSG00000129473 | 1.02 (0.81, 1.28) | 2.39E-03 | 2.07E-02 | nADT vs control (n=43)/Roswell |
| Figure 1b | Clinical Dataset #4 | *BCL2* | BCL-2 | ENSG00000171791 | 5.86 (-2.51, 14.23) | 3.82E-01 | NA | post vs pre-Enza (n=21)/Alumkal |
| Figure 1b | Clinical Dataset #4 | *BCL2L1* | BCL-XL | ENSG00000171552 | 1.16 (0.70, 1.62) | 2.70E-01 | NA | post vs pre-Enza (n=21)/Alumkal |
| Figure 1b | Clinical Dataset #4 | *MCL1* | MCL-1 | ENSG00000143384 | 2.02 (0.45, 3.59) | 1.00E-04 | NA | post vs pre-Enza (n=21)/Alumkal |
| Figure 1b | Clinical Dataset #4 | *BCL2L2* | BCL-W | ENSG00000129473 | 1.35 (0.72, 1.99) | 3.70E-01 | NA | post vs pre-Enza (n=21)/Alumkal |
| Figure 1d | Pre-clinical Dataset #1 | *BCL2* | BCL-2 | ENSG00000171791 | 2.05 (1.71, 2.45) | 2.71E-15 | 4.37E-14 | LNCaP-ARKO (6) vs. AR (4) (in vitro) |
| Figure 1d | Pre-clinical Dataset #1 | *BCL2L1* | BCL-XL | ENSG00000171552 | 0.70 (0.61, 0.80) | 1.21E-07 | 7.65E-07 | LNCaP-ARKO (6) vs. AR (4) (in vitro) |
| Figure 1d | Pre-clinical Dataset #1 | *MCL1* | MCL-1 | ENSG00000143384 | 0.70 (0.64, 0.77) | 3.88E-15 | 6.18E-14 | LNCaP-ARKO (6) vs. AR (4) (in vitro) |
| Figure 1d | Pre-clinical Dataset #1 | *BCL2L2* | BCL-W | ENSG00000129473 | 0.91 (0.78, 1.05) | 2.05E-01 | 3.02E-01 | LNCaP-ARKO (6) vs. AR (4) (in vitro) |
| Figure 1d | Pre-clinical Dataset #2 | *BCL2* | BCL-2 | ENSG00000171791 | 3.17 (2.21, 4.56) | 3.70E-10 | 4.93E-09 | LNCaP-ARKO (7) vs. AR (7) (in vivo, castration) |
| Figure 1d | Pre-clinical Dataset #2 | *BCL2L1* | BCL-XL | ENSG00000171552 | 0.90 (0.75, 1.08) | 2.67E-01 | 3.78E-01 | LNCaP-ARKO (7) vs. AR (7) (in vivo, castration) |
| Figure 1d | Pre-clinical Dataset #2 | *MCL1* | MCL-1 | ENSG00000143384 | 0.57 (0.49, 0.66) | 7.72E-14 | 1.78E-12 | LNCaP-ARKO (7) vs. AR (7) (in vivo, castration) |
| Figure 1d | Pre-clinical Dataset #2 | *BCL2L2* | BCL-W | ENSG00000129473 | 1.21 (1.05, 1.41) | 1.08E-02 | 2.66E-02 | LNCaP-ARKO (7) vs. AR (7) (in vivo, castration) |
| Figure 1d | Pre-clinical Dataset #3 | *BCL2* | BCL-2 | ENSG00000171791 | 1.78 (1.16, 2.74) | 8.83E-03 | 4.87E-02 | LNCaP Pri CRPC (4) vs. AD (4) |
| Figure 1d | Pre-clinical Dataset #3 | *BCL2L1* | BCL-XL | ENSG00000171552 | 0.82 (0.65, 1.03) | 9.24E-02 | 2.68E-01 | LNCaP Pri CRPC (4) vs. AD (4) |
| Figure 1d | Pre-clinical Dataset #3 | *MCL1* | MCL-1 | ENSG00000143384 | 0.87 (0.69, 1.10) | 2.41E-01 | 4.89E-01 | LNCaP Pri CRPC (4) vs. AD (4) |
| Figure 1d | Pre-clinical Dataset #3 | *BCL2L2* | BCL-W | ENSG00000129473 | 1.13 (0.88, 1.46) | 3.39E-01 | 5.94E-01 | LNCaP Pri CRPC (4) vs. AD (4) |
| Figure 1d | Pre-clinical Dataset #4 | *BCL2* | BCL-2 | ENSG00000171791 | 3.83 (2.42, 6.05) | 9.49E-09 | 1.99E-07 | LNCaP Sec CRPC (4) vs. AD (4) |
| Figure 1d | Pre-clinical Dataset #4 | *BCL2L1* | BCL-XL | ENSG00000171552 | 0.83 (0.66, 1.05) | 1.21E-01 | 2.92E-01 | LNCaP Sec CRPC (4) vs. AD (4) |
| Figure 1d | Pre-clinical Dataset #4 | *MCL1* | MCL-1 | ENSG00000143384 | 0.77 (0.60, 0.98) | 3.08E-02 | 1.07E-01 | LNCaP Sec CRPC (4) vs. AD (4) |
| Figure 1d | Pre-clinical Dataset #4 | *BCL2L2* | BCL-W | ENSG00000129473 | 0.99 (0.76, 1.28) | 9.20E-01 | 9.77E-01 | LNCaP Sec CRPC (4) vs. AD (4) |
| Figure 1d | Pre-clinical Dataset #5 | *BCL2* | BCL-2 | ENSG00000171791 | 2.15 (1.36, 3.39) | 1.02E-03 | 4.22E-02 | LNCaP Sec CRPC (4) vs. Pri CRPC (4) |
| Figure 1d | Pre-clinical Dataset #5 | *BCL2L1* | BCL-XL | ENSG00000171552 | 1.02 (0.80, 1.28) | 8.99E-01 | 1.00E+00 | LNCaP Sec CRPC (4) vs. Pri CRPC (4) |
| Figure 1d | Pre-clinical Dataset #5 | *MCL1* | MCL-1 | ENSG00000143384 | 0.88 (0.69, 1.13) | 3.19E-01 | 8.57E-01 | LNCaP Sec CRPC (4) vs. Pri CRPC (4) |
| Figure 1d | Pre-clinical Dataset #5 | *BCL2L2* | BCL-W | ENSG00000129473 | 0.87 (0.67, 1.13) | 2.91E-01 | 8.38E-01 | LNCaP Sec CRPC (4) vs. Pri CRPC (4) |
| Figure 1d | Pre-clinical Dataset #6 | *BCL2* | BCL-2 | ENSG00000171791 | 6.38 (2.38, 17.08) | 2.27E-04 | 9.19E-04 | LAPC9 CRPC (5) vs. AD (5) |
| Figure 1d | Pre-clinical Dataset #6 | *BCL2L1* | BCL-XL | ENSG00000171552 | 0.80 (0.69, 0.93) | 4.34E-03 | 1.28E-02 | LAPC9 CRPC (5) vs. AD (5) |
| Figure 1d | Pre-clinical Dataset #6 | *MCL1* | MCL-1 | ENSG00000143384 | 0.88 (0.76, 1.03) | 1.16E-01 | 2.09E-01 | LAPC9 CRPC (5) vs. AD (5) |
| Figure 1d | Pre-clinical Dataset #6 | *BCL2L2* | BCL-W | ENSG00000129473 | 1.34 (1.15, 1.57) | 2.71E-04 | 1.07E-03 | LAPC9 CRPC (5) vs. AD (5) |

***Note:** RNA-seq expression values (TPM, FPKM, or DESeq2-normalized counts) were used as provided in the original sources. Where applicable, fold-change (FC), p-values, and false discovery rates (FDR) were extracted from the original datasets.

No additional normalization or batch correction was performed unless otherwise specified.

FC and 95% confidence intervals (CI) were calculated using DESeq2 when raw counts were available.

For the Alumkal cohort, where raw counts were unavailable, FC and statistical comparisons were calculated from TPM values using paired one-side Wilcoxon signed-rank tests. NA: DESeq2 analysis not performed due to lack of raw counts in the original study.
